# ARIH2 serves as a potential prognostic biomarker for hepatocellular carcinoma associated with immune infiltration and ferroptosis

**DOI:** 10.3389/fimmu.2025.1548691

**Published:** 2025-04-07

**Authors:** Qiang Shu, Qiang Wang, Xiaoli Yang, Bo Li

**Affiliations:** Department of Hepatobiliary Surgery, Affiliated Hospital of Southwest Medical University, Luzhou, Sichuan, China

**Keywords:** ARIH2, prognostic marker, HCC, therapeutic target, tumour immune microenvironment, ferroptosis

## Abstract

**Background:**

Ariadne homolog 2 (ARIH2) has been demonstrated to be upregulated in various human cancer tissues. Nevertheless, the underlying biological function of ARIH2 in the progression of hepatocellular carcinoma (HCC) remains ambiguous. Hence, we conducted a comprehensive bioinformatics analysis on the liver hepatocellular carcinoma (LIHC) dataset to explore the role of ARIH2 in tumorigenesis.

**Methods:**

The mRNA and protein expression of ARIH2 was analyzed by using data from public databases and verified through immunohistochemical staining and Western blot. Logistic regression, Cox regression, receiver operating characteristic curve (ROC), Kaplan-Meier analysis and nomogram model were employed to assess the association between ARIH2 and the clinicopathological characteristics of HCC. We utilized functional enrichment analysis to investigate the potential pathways of ARIH2 in the progression of HCC. The association of ARIH2 with immune infiltration, ferroptosis and immune checkpoint genes was further evaluated. Finally, the correlation between ARIH2 and the IC50 of chemotherapeutic drugs was analyzed in HCC.

**Results:**

Our study discovered that ARIH2 was up-regulated in HCC tumor tissues compared with the control group. ARIH2 expression could effectively distinguish tumor tissues from normal liver tissues. The genes related to ARIH2 showed differential expression in pathways involving immune system-related pathways and ion channels. We identified a significant association between the expression level of ARIH2 in HCC tissues and immune infiltration, immune checkpoint genes and ferroptosis. The expression level of ARIH2 was significantly correlated with the clinical stage, histological pathological grade and clinical characteristics of HCC, and could independently predict overall survival.

**Conclusions:**

The expression level of ARIH2 may serve as a promising biomarker for the diagnosis and prognosis of HCC, as well as a potential drug target, which holds great significance for the development of targeted therapy for HCC.

## Introduction

Liver cancer constitutes a significant global health concern, ranking as the sixth most prevalent cancer and the third leading cause of cancer-related deaths worldwide (1). Among these, HCC constitutes 75%-85% of liver cancer cases (1). Despite advancements in treatment approaches, the current treatment options for HCC, such as surgical resection, liver transplantation, local treatment, and systemic treatment, are often influenced by late diagnosis, distant metastasis, and a high recurrence rate, resulting in an unsatisfactory overall survival rate (2). With the advent of immunotherapy, immune checkpoint inhibitors (ICIs) have brought about a breakthrough in the management of HCC (3). However, due to tumor heterogeneity and alterations in the immune microenvironment, not all patients reap survival benefits (4). Hence, it is imperative to conduct further studies on the molecular characteristics of HCC occurrence and development to identify novel biomarkers, predict prognosis, and guide individualized clinical treatment (5).

ARIH2, a member belonging to the Ariadne subfamily of RBR E3 ligases, is engaged in the ubiquitination of target proteins and exerts a crucial role in post-translational modifications within diverse cellular processes (6). Presently, the regulatory function of ARIH2 in numerous malignant cancers has been probed. Evidence indicates that ARIH2 plays an essential part in the development and progression of gastric cancer, acute myeloid leukemia, human non-small cell lung cancer, and other malignancies (7–9). For instance, a recent study revealed that ARIH2 promotes the proliferation of gastric cancer cells by reducing p21 stability through ubiquitination (7). The down-regulation of ARIH2 expression can trigger DNA damage and apoptosis in GC cells, and heighten the drug sensitivity to 5-fluorouracil (7). It was also discovered that ARIH2 knockout could augment the resistance of NSCLC to EGFR tyrosine kinase inhibitor (TKI) (10). In recent years, a multitude of studies have determined that ARIH2 can function as a regulator and plays a significant role in immune response and inflammatory diseases (11, 12). Nevertheless, the connection between ARIH2 and the early diagnosis, prognosis, and immune infiltration of HCC remains indistinct.

The purpose of this study was to explore the transcriptional expression and prognostic significance of ARIH2 in HCC by employing an interactive tool. Through the utilization of bioinformatics techniques, we inspected the relationship between ARIH2 expression and clinicopathological features, prognostic significance, and immune cell infiltration, explored the biological mechanism of ARIH2 in the progression of HCC, and assessed the feasibility of ARIH2 as a potential therapeutic target.

## Collection of samples

During the period from June 2024 to September 2024, 12 pairs of HCC and paracancerous tissues that were confirmed through pathological examination were collected from patients who underwent liver cancer resection at the Department of Hepatobiliary Surgery of the Affiliated Hospital of Southwest Medical University. The entire experimental protocol adhered strictly to the ethical guidelines set forth by the Ethics Committee of the Affiliated Hospital of Southwest Medical University. Informed consent was obtained from all participants and/or their legal guardians prior to their involvement in the study.

### Cell culture and experiments

Normal hepatocyte THLE-2 and HCC cell lines, including HCCLM3, MHCC97-H, HepG2, Hep3B, and Huh-7, were acquired from the Science and Technology Experimental Center of Southwest Medical University.The cells were maintained in DMEM with 10% fetal bovine serum and 100 U/mL penicillin/streptomycin at 37**°**C and 5% CO_2_ with saturated humidity.

### Immunohistochemistry

Paraffin-embedded tumors were sliced into 5mm thick sections, and then the paraffin sections underwent deparaffinization and dehydration. Subsequently, the paraffin sections were heated in citrate buffer with a pH of 6.0 within a microwave oven to 95°C for 20 minutes to facilitate antigen retrieval. Thereafter, endogenous peroxidase activity was inhibited, followed by blocking with normal goat serum. ARIH2 antibodies were subsequently diluted with BSA in accordance with the manufacturer’s instructions, and the antibodies were added to the paraffin sections and incubated overnight at 4°C. Horseradish peroxidase-linked secondary antibodies were added and incubated with the sections prior to the addition of DBA reagent.The reaction products were observed under a microscope after counterstaining with 0.1% hematoxylin for 2 minutes at room temperature. Photographs of the relevant sections were captured at a microscopic magnification of ×200. The protein expression level were rated based on the staining intensity of malignant/epithelial cells and the proportion of immunoreactive cells. The IHC scores were as follows: unstained tissue = 0, 20% of cells with weakly or moderately to strongly stained =1, 20% to 40% of cells with moderately or heavily stained =2, more than 40% strongly stained cells =3.

### Western blotting

HCC tissues and cells were lysed with the aid of RIPA, and proteins were subsequently extracted. The protein concentration was ascertained by the BCA Protein Quantification Kit (P0010, Beyotime, Shanghai, China). Proteins of varying molecular weights were segregated through SDS-polyacrylamide gel electrophoresis and electrotransferred onto polyvinylidene difluoride membranes. The membranes are sealed with skimmed milk, and the membrane sections were successively incubated with primary and secondary antibodies. The membranes were exposed to an extremely hyper-sensitive ECL developing reagent (36208ES60, Yeasen, Guangzhou, China) and visualized via the Western blot analysis detection system (Thermo Fisher, Shanghai, China).

### Transfection with small interfering RNA

Huh7 and HCCLM3 cells(3×10^5/well)were seeded in 6-well plates and subsequently transfected with ARIH2-specific siRNA or a negative control siRNA according to the manufacturer’s protocol (JIan, Suzhou, China). Specifically, 4μL of siRNA at a concentration of 20 μmol/L was combined with 46 μL of GA-RNA BUFFER and thoroughly mixed with 7.5μL of GA-RNA Reagent. This mixture was then added to the 6-well plates containing cell culture medium supplemented with 10% FBS. The cells were incubated under standard conditions for 36 hours.The sequences used for ARIH2 knockdown were as follows:

 -siRNA1#:Sense,5’-GGAAGAAGCUGUUUGAAUATT-3’;Antisense,5’-UAUUCAAACAGCUUCUUCCTT-3’
 -siRNA2#:Sense,5’-GCAAUGCUCCAAAUGUAAATT-3’;Antisense,5’-UUUACAUUUGGAGCAUUGCTT-3’
 -siRNA3#:Sense,5’-GGGACUAUGUGGAGAGUCATT-3’;Antisense,5’-UGACUCUCCACAUAGUCCCTT-3’
 The sequence for the negative control siRNA was:
 -Sense,5’-UUCUCCGAACGUGUCACGUTT-3’;Antisense,5’-ACGUGACACGUCGGAGAATT-3’

### Cell viability assay

Transfected Huh7 and HCCLM3 cells (3,000/well) were seeded in 96-well plates. At 24, 48, and 72 hours post-seeding, 10 µL of CCK-8 solution (MedChemExpress, USA) was added to each well. After a 2-hour incubation period, the absorbance at 450nm was measured using a microplate reader (BioTek, Winooski, VT, USA).

### Wound healing assay

Transfected Huh7 and HCCLM3 cells were cultured in 6-well plates until confluent. Non-adherent cells were removed by rinsing with PBS, after which the monolayer was scratched using a 200µL pipette tip. Cells were then cultured in serum-free medium and imaged at 0 and 48 hours post-scratch. Data were analyzed using Image J 2.3.0.

### Data sources and processing

The mRNA expression profile of ARIH2 in pan-cancer and normal tissues was retrieved from the TCGA and GTEx databases (13). Subsequently, the mRNA expression profile of ARIH2 in HCC tissues and adjacent tissues were acquired from the TCGA database(https://www.cancer.gov/ccg/research/genome-sequencing/tcga) and the ICGC database (https://dcc.icgc.org/). The GSE45267 dataset was incorporated into this research by searching the GEO database. Xiantao tool (https://www.xiantao.love/ is a useful bioinformatics analysis web tool, and was used for visualization.

### Analysis on the differential expression of ARIH2

The Wilcoxon rank sum test was utilized to appraise the differential expression of ARIH2 in pan-cancer. Shapiro-Wilk normality analysis was implemented on the ARIH2 expression profile data in paired and unpaired samples, followed by the Wilcoxon rank-sum test. Logistic regression was applied to analyze the relationship between the expression level of ARIH2 and the clinicopathological characteristics of HCC patients. The protein expression of ARIH2 in HCC was evaluated via the CTPAC database. Based on the median expression level of ARIH2, the patients were classified as the high expression group and the low expression group. To further investigate the differences in gene expression between these two groups, the researchers utilized the R package DESeq2 in Xiantao tool. The threshold for differentially expressed genes (DEGs) was set as an adjusted p-value<0.05 and | log_2_-fold change (FC) |>1. Cytoscape and STRING database were employed to construct the PPI network, and MCODE was utilized to screen hub genes. Statistical significance was defined as p-value < 0.05 for all the aforementioned analyses.

### Functional enrichment analysis

The differentially expressed genes associated with ARIH2 were acquired for GO and KEGG enrichment analyses. GO terms are categorized into three aspects: biological process (BP), molecular function (MF), and cellular composition (CC). Through the analysis of the genes associated with ARIH2, researchers can obtain an understanding of the specific biological processes, molecular functions, and cellular components related to this gene. Furthermore, KEGG pathway enrichment analysis empowered the researchers to explore the potential pathways associated with ARIH2. The KEGG pathway database compiles various biological pathways and offers a comprehensive understanding of the molecular interactions and signalling events related to a specific gene or set of genes. GSEA, which represents Gene Set Enrichment Analysis, is a computational method utilized to normalize RNA Seq data obtained from TCGA. This analysis tool, accessible on the MSigDB website, facilitates researchers in studying the biological function of ARIH2. Select gene sets “h.all.v2022.1.Hs.symbols.gmt [Hallmarks]” GSEA analysis (14). We set the enrichment attention threshold as a false discovery rate (FDR) < 0.25, p.adjust < 0.05

### Immune infiltration

The CIBERSORT immune score was adopted to ascertain the proportion of ARIH2 in 22 immune cells (15). Based on the high and low expression groups of ARIH2, we further employed single-sample gene set enrichment analysis (ssGSEA) to evaluate the correlation between ARIH2 and the infiltration levels of 24 immune cells (16). The connections between ARIH2 and immune checkpoint genomic expression profiles were analyzed by means of Spearman and Wilcoxon rank-sum tests. Additionally, disparities in immune checkpoint expression and the Tumor Immune Dysfunction and Exclusion (TIDE) score were inspected between the two groups. The potential immunotherapy response was predicted via the TIDE algorithm (17). The aforementioned analytical modules and resultant displays have been implemented within the Xiantao Tool. The prognosis of patients with HCC influenced by the ARIH2 expression level and the level of immune cell infiltration was probed through the online database kmplot (http://kmplot.com/analysis/). And the “chemokine” module in the TISIDB (http://cis.hku.hk/TISIDB/) database was utilized for the analysis of chemokines and their receptors to investigate the link between the expression level of ARIH2 and immune cells.

### Relationships between expression of ARIH2 and ferroptosis-associated genes

The potential interrelationships between the expression of ARIH2 and that of ferroptosis-associated genes were assessed within the TCGA-LIHC datasets through the application of Xiantao tool. Additionally, Xiantao tool was also exploited to ascertain the proportions of ferroptosis-associated genes in samples presenting with high and low ARIH2 levels. The “box plot” package incorporated within Xiantao tool was utilized to visually exhibit the results.

### Sensitivity analysis of anticancer drugs

We downloaded the relevant data of 357 drugs from the Genomics of Drug Sensitivity in Cancer (GDSC) database. The link between ARIH2 expression and the half maximal inhibitory concentration (IC50) in each sample was predicted using the “pRRophetic” (18). Ridge regression was used to determine the half-maximal IC50 of the samples. The outcomes were visualized with Xiantao tool.

### Clinical statistical analysis, model construction and prognosis assessment

To appraise the influence of various clinical variables on the prognosis of HCC patients, univariate and multivariate Cox regression analyses were implemented. In the univariate Cox regression analysis, prognostic variables with a significance level of p < 0.05 were recognized. These significant variables were further evaluated in the multivariate Cox regression analysis. The Xiantao tool was utilized to generate forest plots, which displayed the results of univariate and multivariate Cox regression analyses. Kaplan-Meier methods were employed to analyze overall survival (OS), disease-specific survival (DSS), and progression-free interval (PFI) in HCC patients with high and low ARIH2 expression. The online databases GEPIA2 (http://gepia2.cancer-pku.cn/) and kmplot (http://kmplot.com/analysis/) were utilized to verify the ARIH2 expression level of the OS in patients with HCC. Based on the outcome measures from univariate Cox regression analysis, we constructed nomograms featuring independent prognostic measures and predicted the 1-year, 3-year, and 5-year survival. Furthermore, we utilized the concordance index (C-index) to assess the discrepancy between the predicted and observed survival probabilities. Calibration diagrams were adopted to determine the accuracy of the nomogram predictions.

## Results

### The expression and prognosis of ARIH2 in diverse cancerous tissues and adjacent normal tissues were assessed

**Figure 1:**
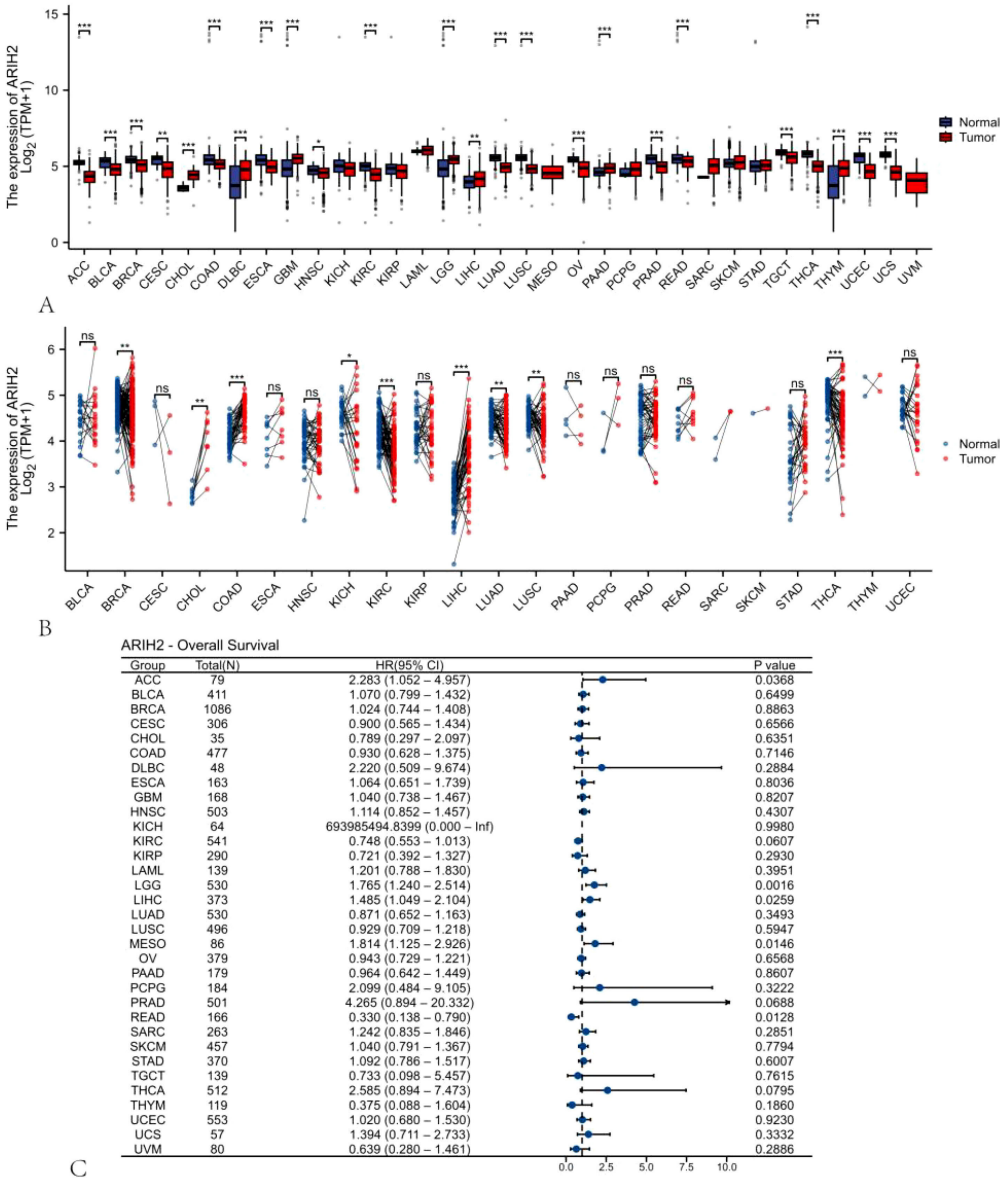
The expression pattern and prognostic value of ARIH2 from a pan-cancer perspective. **(A, B)** The expression levels of ARIH2 in diverse cancerous tissues and adjacent normal tissues are presented based on the TCGA and GTEx databases. **(C)** Cox regression analysis is conducted to examine the association between ARIH2 expression and diverse human cancers. *NS, p* > 0.05, *p < 0.05, **p < 0.01, ***p < 0.001.

We probed into the expression levels of ARIH2 in 33 cancerous tissues and adjacent tissues through the utilization of the TCGA dataset and GTEX database. A significant association between ARIH2 expression and normal tissue was noted in 24 of 33 cancers (**Figure 1A**). We also inspected the expression of ARIH2 in human tumor tissues and Paracancerous tissues by means of the TCGA dataset. We discerned that ARIH2 expression was significantly elevated in 9 of 23 cancers compared with Paracancerous tissue (**Figure 1B**). Furthermore, based on the expression level of ARIH2, Cox regression analysis of 33 cancers demonstrated that the ARIH2 expression level was related to the prognosis of ACC (adrenocortical carcinoma), LGG(brain lower grade glioma), LIHC (liver hepatocellular carcinoma), MESO (mesothelioma), and READ(Rectum adenocarcinoma) (**Figure 1C**).

### The expression of ARIH2 is up-regulated in HCC

**Figure 2:**
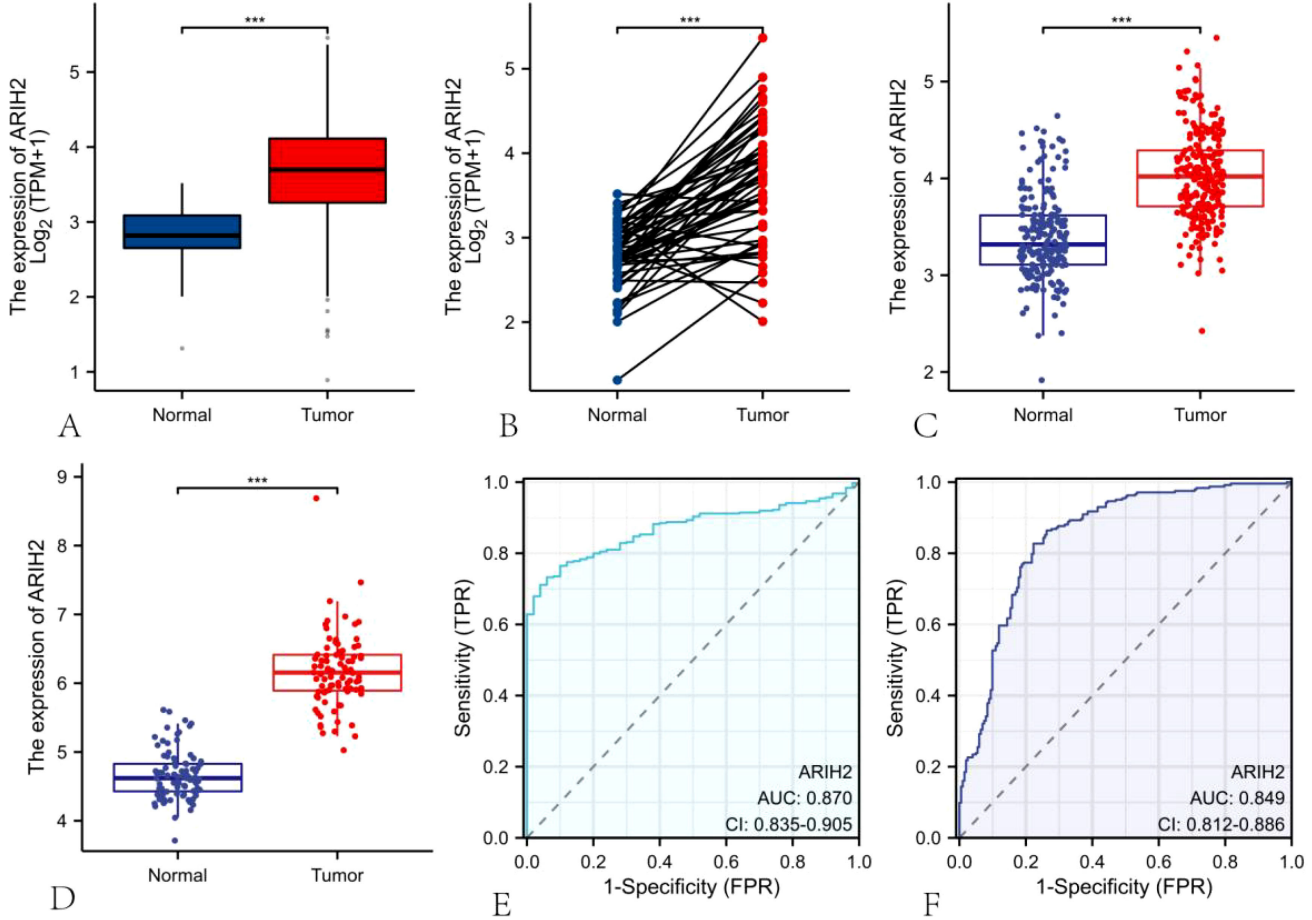
The mRNA expression of ARIH2 in LIHC. **(A-B)** The mRNA expression levels of ARIH2 in HCC tissues and Paracancerous tissue from the TCGA and GTEx databases are illustrated. **(C)** The mRNA expression levels of ARIH2 in HCC tissues from the ICGC database are provided. **(D)** The mRNA expression level of ARIH2 in HCC tissues of the GSE45267 dataset is displayed. **(E, F)** The ROC curve analysis of ARIH2 in HCC patients was conducted based on the TCGA database and the ICGC database. ***p < 0.001.

We discovered that ARIH2 expression was highly elevated in LIHC (**Figure 2A**). We also identified that ARIH2 expression was significantly higher in LIHC than in paired adjacent normal tissues (**Figure 2B**). To confirm this, we utilized the ICGC database and GEO dataset to evaluate the expression level of ARIH2 in HCC. The results affirmed that the expression levels of ARIH2 were significantly increased in HCC tissues compared with Paracancerous tissue (**Figures 2C-D**). In addition, the receiver operating characteristic (ROC) curve analysis was carried out on HCC patients in the TCGA and ICGC databases respectively, and the area under curve (AUC) values were determined to be 0.870 and 0.849 respectively, which demonstrated that the expression of ARIH2 exhibited a high level of accuracy in predicting the presence of the disease (**Figures 2E-F**). This indicates that ARIH2 may serve act as a reliable biomarker for the diagnosis of HCC and may contribute to the early diagnosis and effective management of the disease.

**Figure 3:**
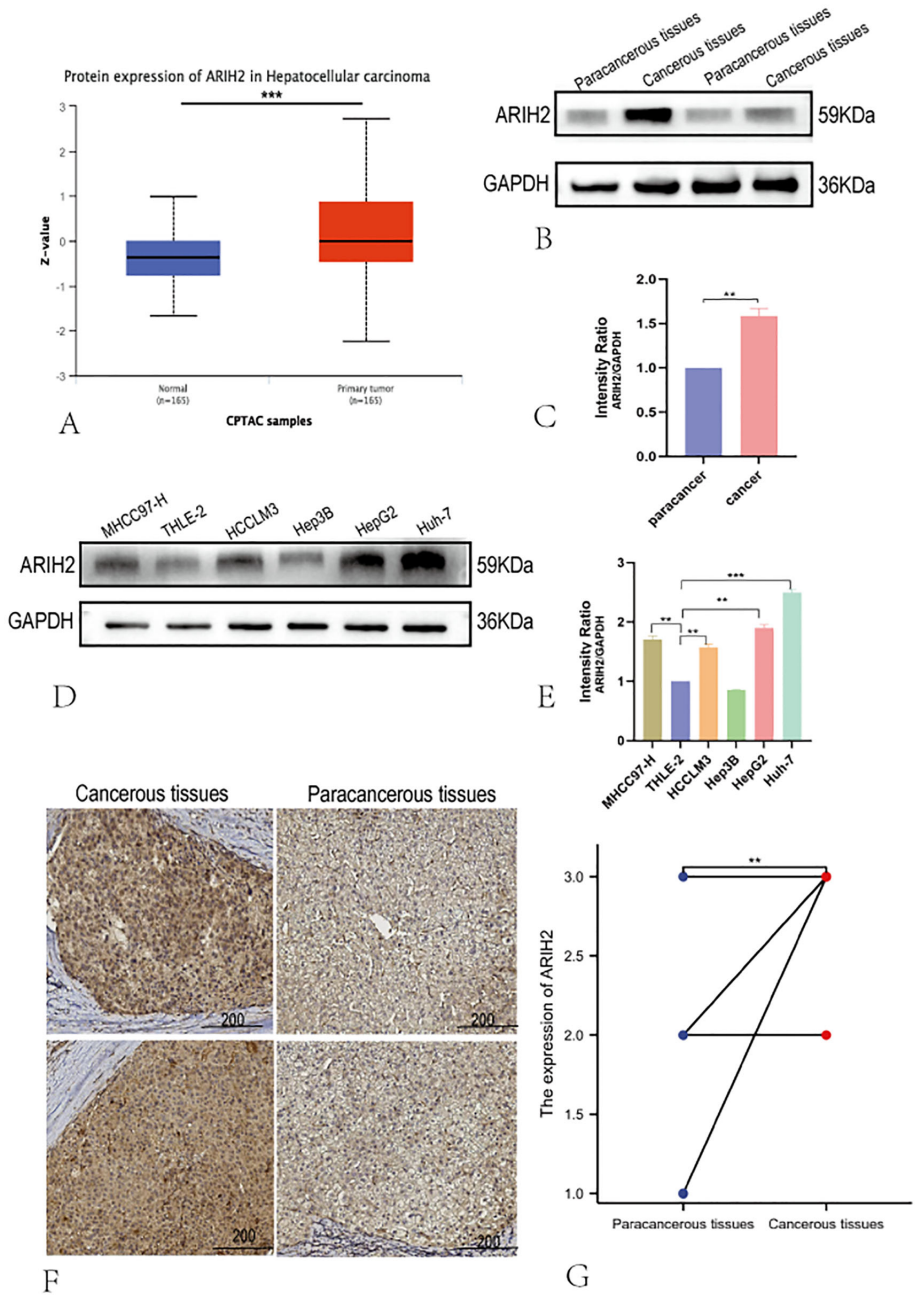
The Protein expression of ARIH2 in HCC tissues. **(A)** The expression of ARIH2 in HCC within the CTPAC database; **(B-C)** Western blot was employed to detect the expression of ARIH2 in HCC and Paracancerous tissues. **(D-E)** Western blot was utilized to detect the expression of ARIH2 in normal hepatocytes (THLE-2) and HCC cell lines (MHCC97-H, HCCLM3, Hep3B, HepG2, Huh-7). **(F)** Representative images of ARIH2 IHC staining in HCC tissues and Paracancerous tissues were presented. **(G)** Intensity IHC scores of cancer and paracancerous tissues in 12 HCC patients were analyzed (Wilcoxon signed-rank test). **p < 0.05.

We conducted a thorough analysis by applying diverse experimental techniques to further assess the expression level of ARIH2 in HCC tissues and determine its significance. Firstly, the expression level of ARIH2 protein in HCC tissues was evaluated via the CTPAC database, and it was revealed that ARIH2 protein expression was higher in HCC tissues than in adjacent normal tissues (**Figure 3A**). Then, we randomly selected 2 pairs of HCC tissues and Paracancerous tissues for WB. The WB results affirmed that ARIH2 protein expression was significantly augmented in HCC tissues compared with Paracancerous tissues (**Figures 3B-C**). To further substantiate these findings, WB was employed to detect the expression level of ARIH2 protein in HCC cell lines and normal hepatocytes. The WB results manifested that the expression levels of ARIH2 protein were significantly heightened in HCC cell lines compared with normal hepatocytes (**Figures 3D-E**). IHC staining scores also evinced that the expression levels of ARIH2 protein were significantly increased in HCC tissues (**Figures 3F-G**). Taken collectively, ARIH2 is up-regulated in HCC tissues and may play a crucial role in HCC carcinogenesis.

### The overexpression of ARIH2 is correlated with adverse clinical parameters in HCC

**Figure 4:**
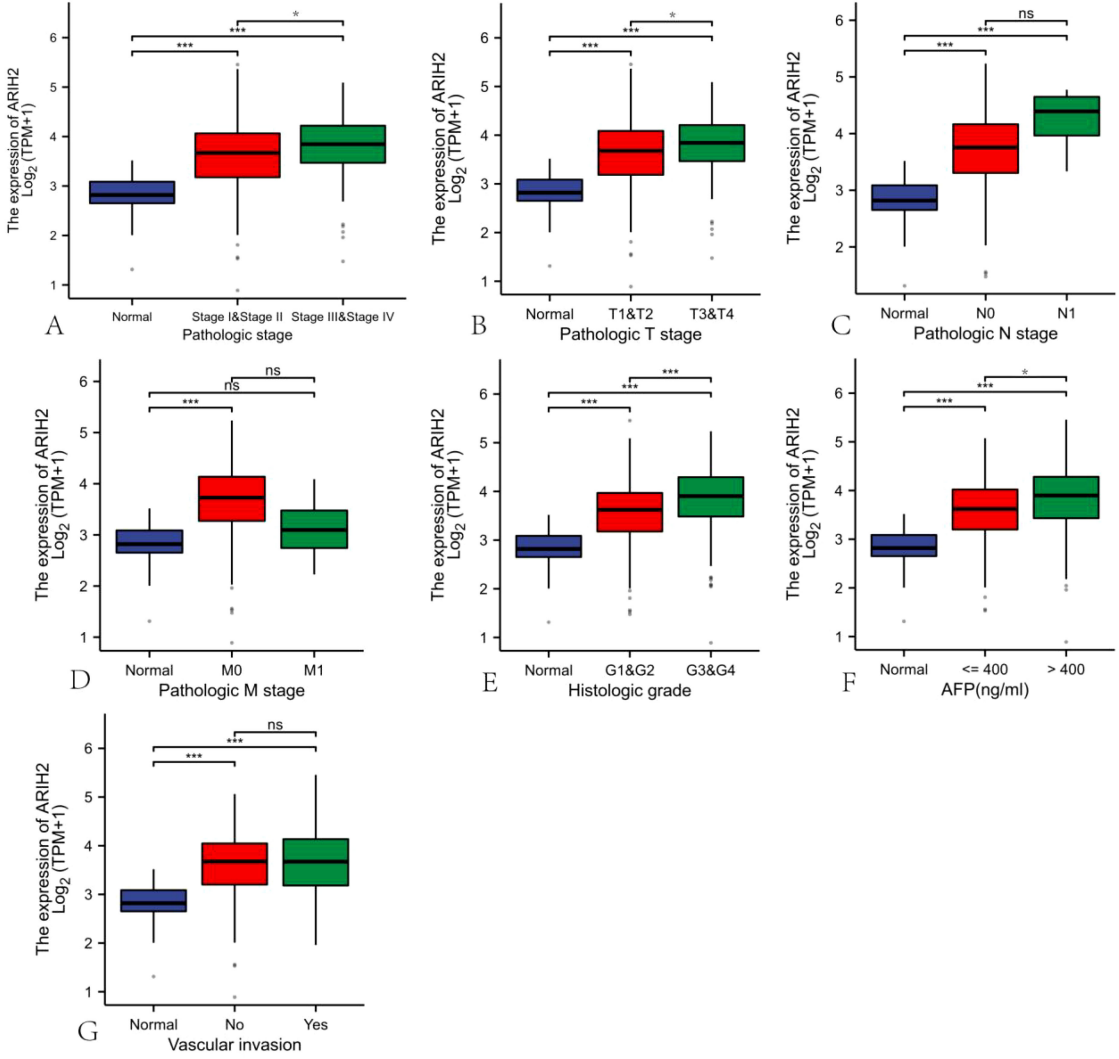
Correlation of ARIH2 expression with clinical parameters of HCC.**(A)** Pathologic stage.**(B)** Pathologic T stage. **(C)** Pathologic N stage. **(D)** Pathologic M stage.**(E)** Histologic grade.**(F)** AFP.**(G)** Vascular invasion. *NS, p* > 0.05, *p < 0.05, **p < 0.01, ***p < 0.001.

The data of 374 HCC patients were retrieved from TCGA. Patients were dichotomized into two groups based on the median ARIH2 expression (N-low = 187 and N-high = 187, respectively), as presented in **Supplementary Table 1**. We conducted a further analysis of the relationship between ARIH2 expression levels and the clinicopathological features of HCC. As illustrated in **Figures 4A-G**, the overexpression of ARIH2 was significantly related to Pathologic stage, TNM stage, Histologic grade, alpha-fetoprotein (AFP), and Vascular invasion. These findings suggest that the overexpression of ARIH2 may be positively associated with the malignant phenotype of HCC.

### The overexpression level of ARIH2 is associated with the unfavourable prognosis of HCC

**Figure 5:**
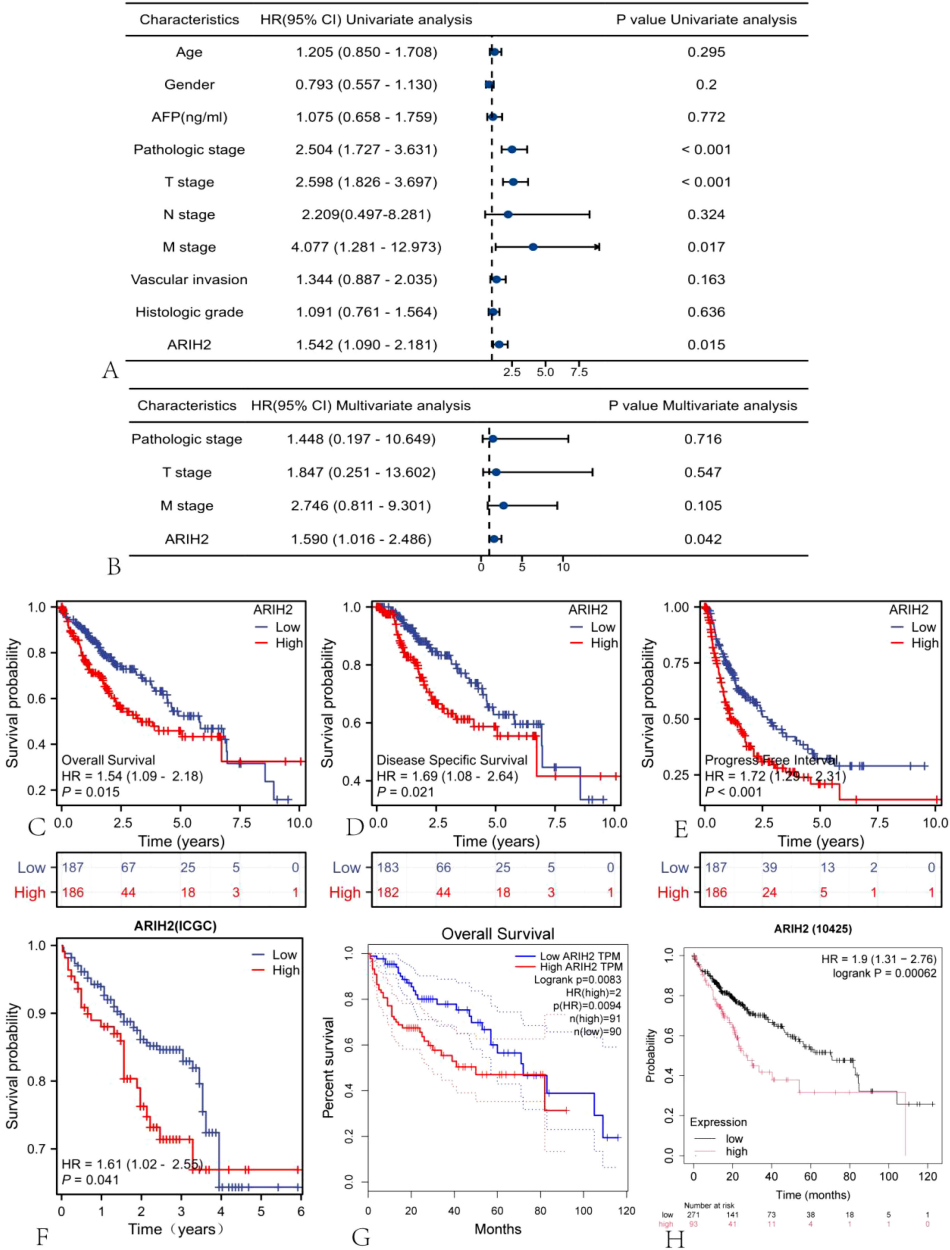
The expression level of ARIH2 is correlated with the prognosis of HCC. Forest plots of OS in HCC patients based on **(A)** univariate and **(B)** multivariate Cox analyses were presented. The Kaplan-Meier method was adopted to compare the relationship between OS **(C)**, DSS **(D)**, and PFI **(E)** and the expression level of ARIH2. The Kaplan-Meier method was exploited to compare the relationship between OS and the expression level of ARIH2 in ICGC **(F)**, GEPIA2 **(G)**, and KM Plotter **(H)** databases.

Univariate and multivariate Cox regression analyses were implemented to explore the relationship between relevant clinical parameters and the overall survival of HCC. Univariate regression analysis revealed that ARIH2 (p = 0.015), Pathologic stage (p < 0.001), Pathologic T stage (p < 0.001), and Pathologic M stage (p = 0.017) were conspicuously correlated with overall survival (**Figure 5A**). Based on multivariate regression analysis, ARIH2 was the sole statistical parameter associated with overall survival (p = 0.042, **Figure 5B**), suggesting that ARIH2 is an independent predictor for the survival prognosis of HCC. The KM method was utilized to analyze the link between the expression level ARIH2 and the prognosis (OS, DSS, and PFS) of HCC patients in the TCGA database. The results indicated that higher ARIH2 expression level was significantly associated with poorer OS (p = 0.015), DSS (p = 0.021), and PFS (p < 0.001) in HCC (**Figures 5C-E**). To confirm the aforesaid results, we analyzed the ICGC database and found that the upregulation of ARIH2 expression was significantly associated with poorer OS (p = 0.041) in HCC patients (**Figure 5F**). By analyzing the online databases GEPIA2 and KM Plotter, the aforementioned results were replicated once again (**Figures 5G-H**).

### The established and validation of ARIH2-related nomograms

**Figure 6:**
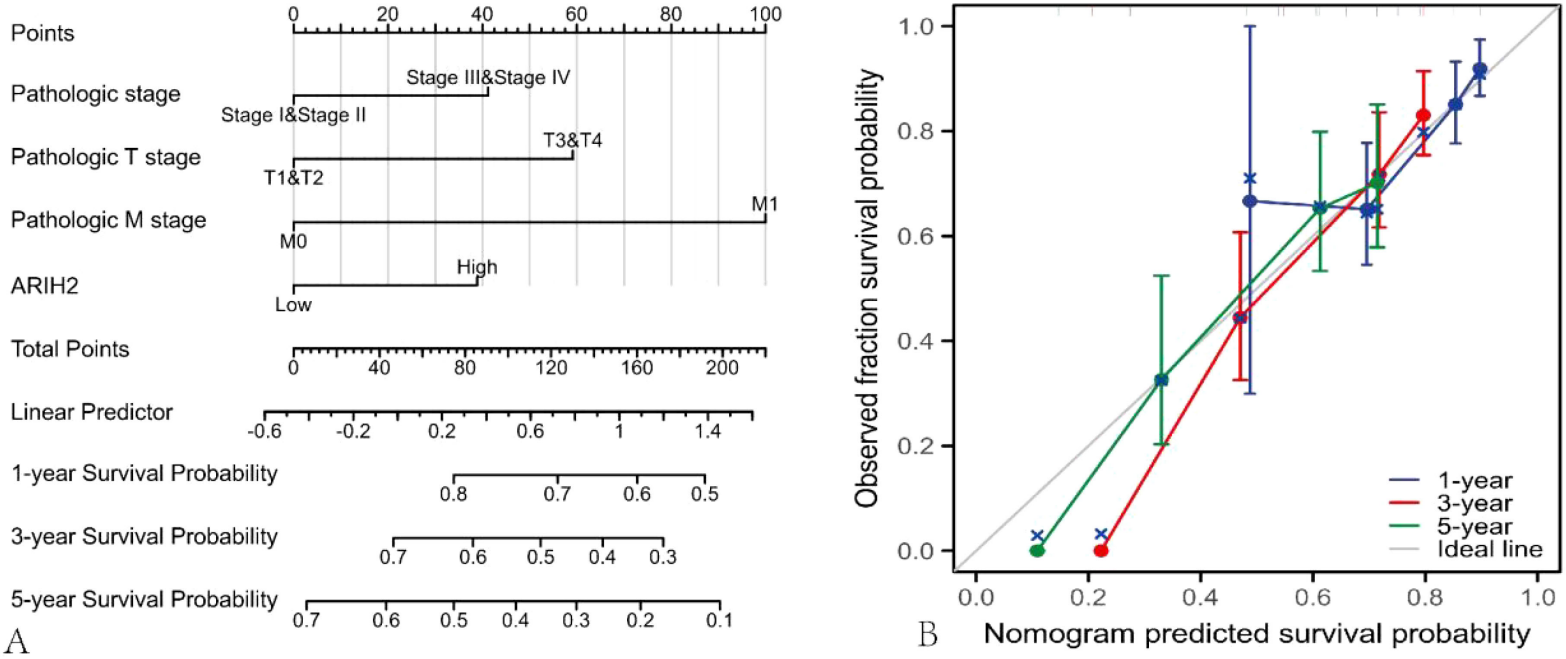
Nomograms and calibration plot were employed to predict the OS rate of HCC patients. **(A)** Nomogram for predicting the 1-, 3-, and 5-year OS of HCC. **(B)** Calibration chart of the Nomogram.

The nomogram was utilized to evaluate the relationship among the main treatment outcomes of HCC patients, such as ARIH2, Pathologic stage, Pathologic T stage, Pathologic M stage, and the 1-, 3-, and 5-year survival prognosis [C-index: 0.706 (0.667-0.744), **Figure 6A**]. The calibration plot (**Figure 6B**) projected the nomogram of 1-, 3-, and 5-year survival prognosis, suggesting that the bias correction line was proximate to the ideal curve, and the predicted value of the nomogram was in good agreement with the actual results.

### Correlation between ARIH2 and prognosis in subgroups of HCC patients

**Figure 7:**
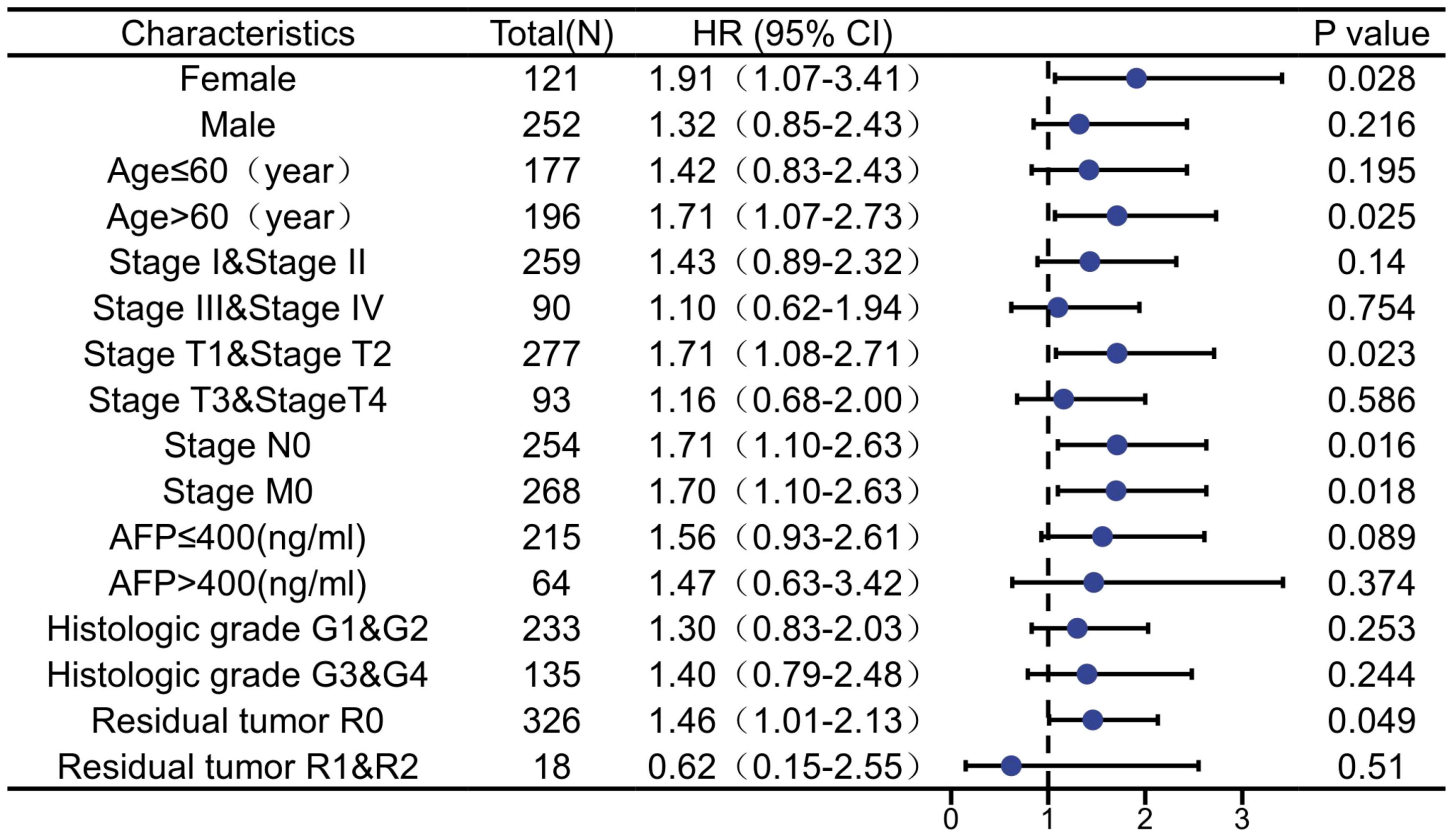
The expression level of ARIH2 was prognostic predictors in subgroups of HCC patients.

The Predictive ability of ARIH2 expression in relation to the clinicopathological characteristics of subgroups of HCC patients was assessed using Cox regression analysis (**Figure 7**). Elevated the expression level of ARIH2 was significantly associated with reduced OS in female patients (HR=1.91, P=0.028), particularly in those of advanced age (HR=1.71, P=0.025), early tumor stages (T1&T2, HR=1.71, P=0.023), absence of lymph node metastasis (N0, HR=1.71, P=0.023), and no distant metastasis (M0, HR=1.70, P=0.018). Additionally, residual tumor (R0, HR=1.46, P=0.049) was also associated with a higher ARIH2 expression level. These findings suggest a significant correlation between increased ARIH2 expression and poorer survival outcomes in HCC patients.

### DEGs between high and low ARIH2 expression groups

**Figure 8:**
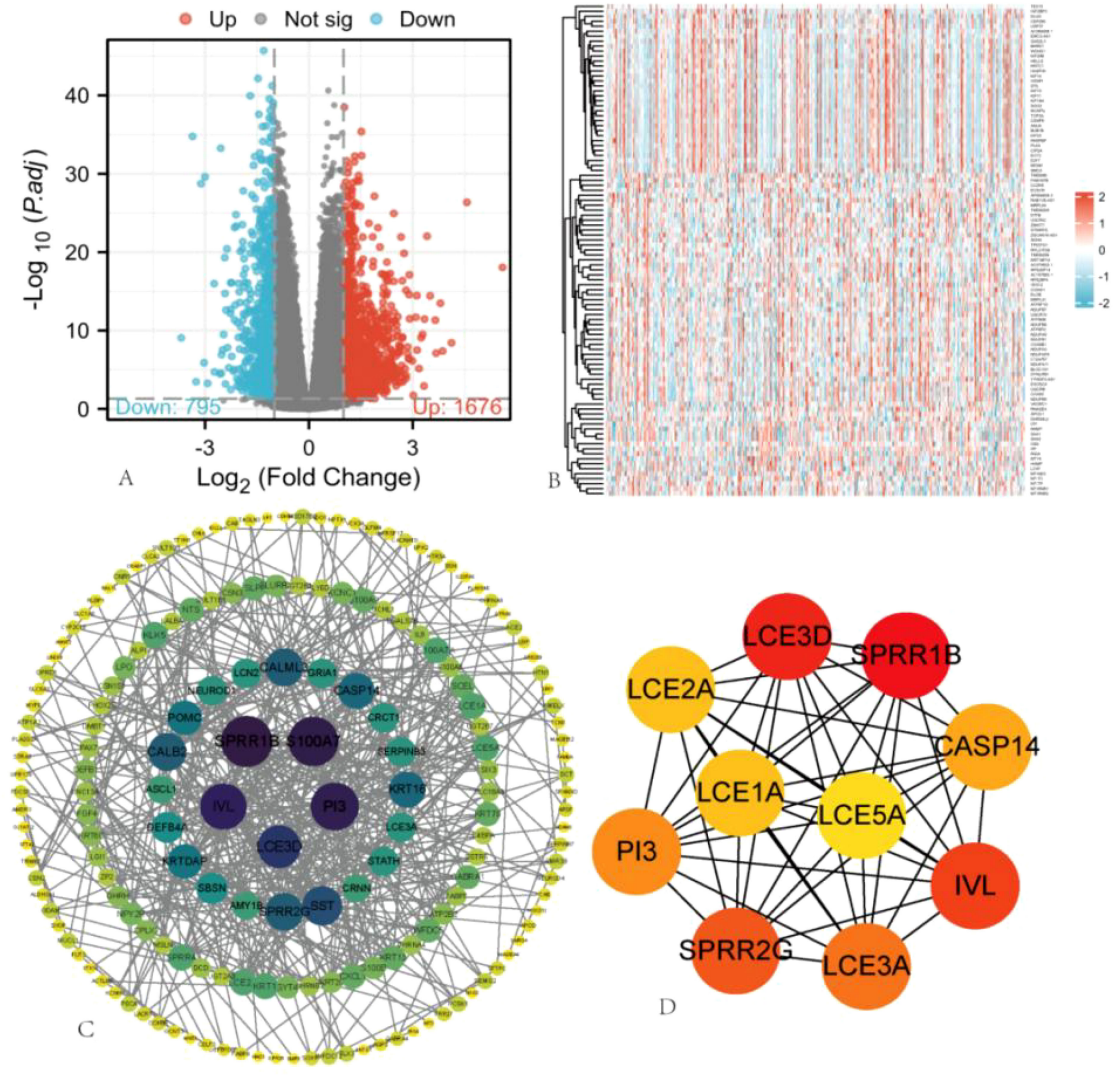
Single Gene Differential Expression Analysis of ARIH2. **(A)** In the volcano plot, blue and red dots represent downregulated and upregulated DEGs, respectively. **(B)** Heatmap illustrating the top 100 gene sets with differential expression related to ARIH2. **(C)** Protein-protein interaction (PPI) network of hub genes. **(D)** The top 10 hub genes were identified.

Based on the median expression value of ARIH2 in HCC from the TCGA database, samples were categorized into high-expression and low-expression groups. The differential expression analysis results between these two groups are illustrated in the volcano plot (**Figure 8A**), which identified 2471 DEGs, comprising 1676 upregulated genes and 795 downregulated genes. The top 100 gene sets exhibiting differential expression relationships with ARIH2 were subsequently visualized as a heatmap (**Figure 8B**). The Cytoscape (v3.10.1) software was utilized to construct the protein-protein interaction network of all DEGs using the STRING database(**Figure 8C**). Furthermore, the CytoHubba plugin was employed to screen for core genes among the top 10, which included LCE5A, LCE1A, LCE2A, LCE3D, SPRR1B, CASP14, IVL, LCE3A, SPRR2G, and PI3(**Figure 8D**). The hub genes identified above were stratified into high and low expression groups based on the median value. The KM method was employed to analyze the overall survival rates, revealing that the expression levels of these genes significantly influenced the overall survival of patients with HCC (**Supplementary Figure 1**).

### Gene function annotation and pathway analysis

**Figure 9:**
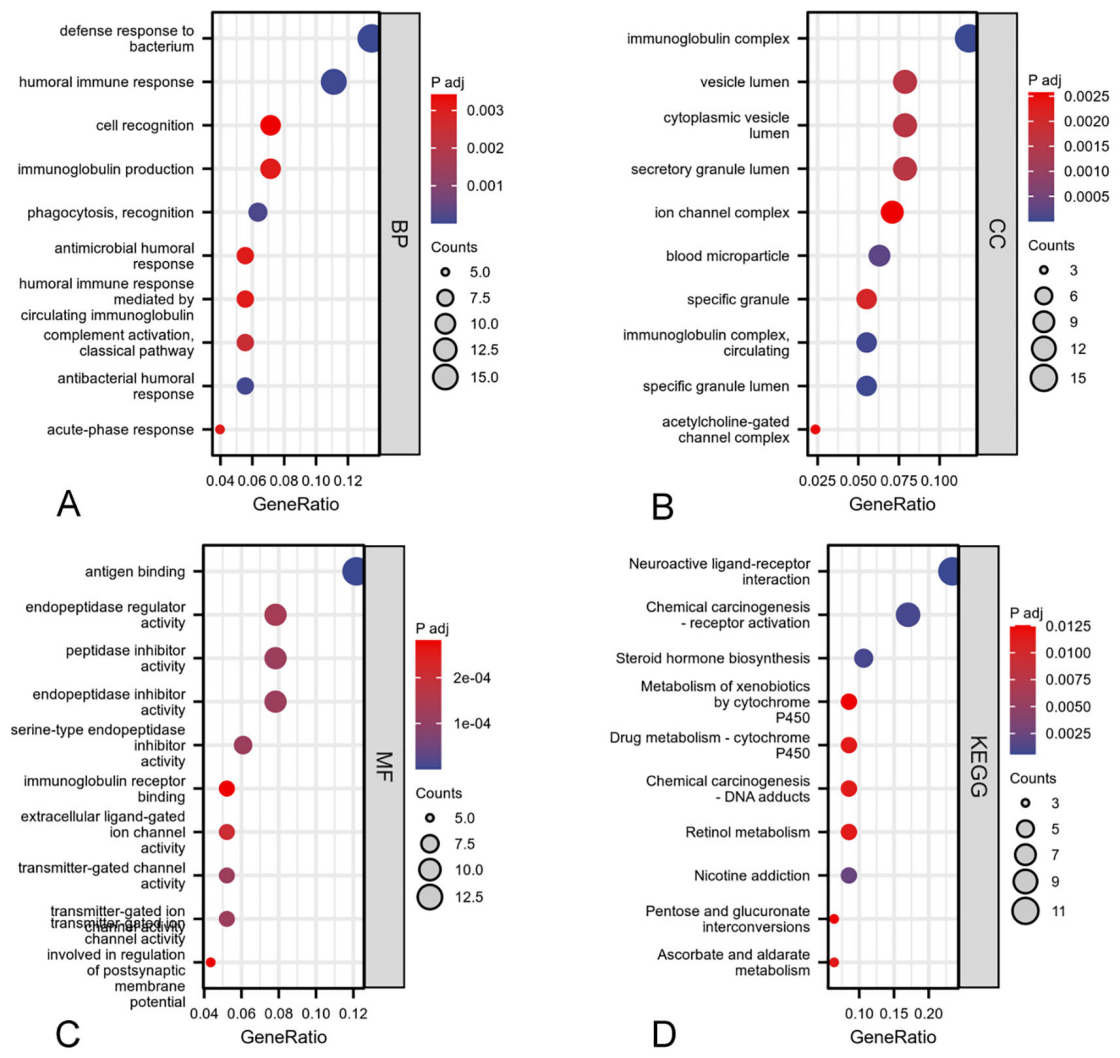
GO and KEGG enrichment analyses of DEGs associated with ARIH2: biological process **(A)**, cellular component **(B)**, molecular function **(C)**, KEGG pathway enrichment analysis **(D)**.

GO and KEGG enrichment analyses were conducted on the 2471 DEGs identified. The GO enrichment analysis revealed alterations in the biological processes (BP), cellular components (CC), and molecular functions (MF) associated with ARIH2, including humoral immune response, phagocytosis and recognition, complement activation and classical pathway, immunoglobulin production、acute-phase response, immunoglobulin complex, blood microparticle, cytoplasmic vesicle lumen, acetylcholine-gated channel complex, ion channel complex, humoral immune response mediated by circulating immunoglobulin, cell recognition, immunoglobulin complex, immunoglobulin complex and circulating correlations (**Figure 9A-C**). The KEGG molecular pathways encompassed neuroactive ligand-receptor interactions, steroid hormone biosynthesis, retinol metabolism, ascorbate and aldarate metabolism, pentose and glucuronate interconversions (**Figure 9 D**).

### Gene set enrichment analysis was employed to investigate the potential associated signaling pathways

**Figure 10:**
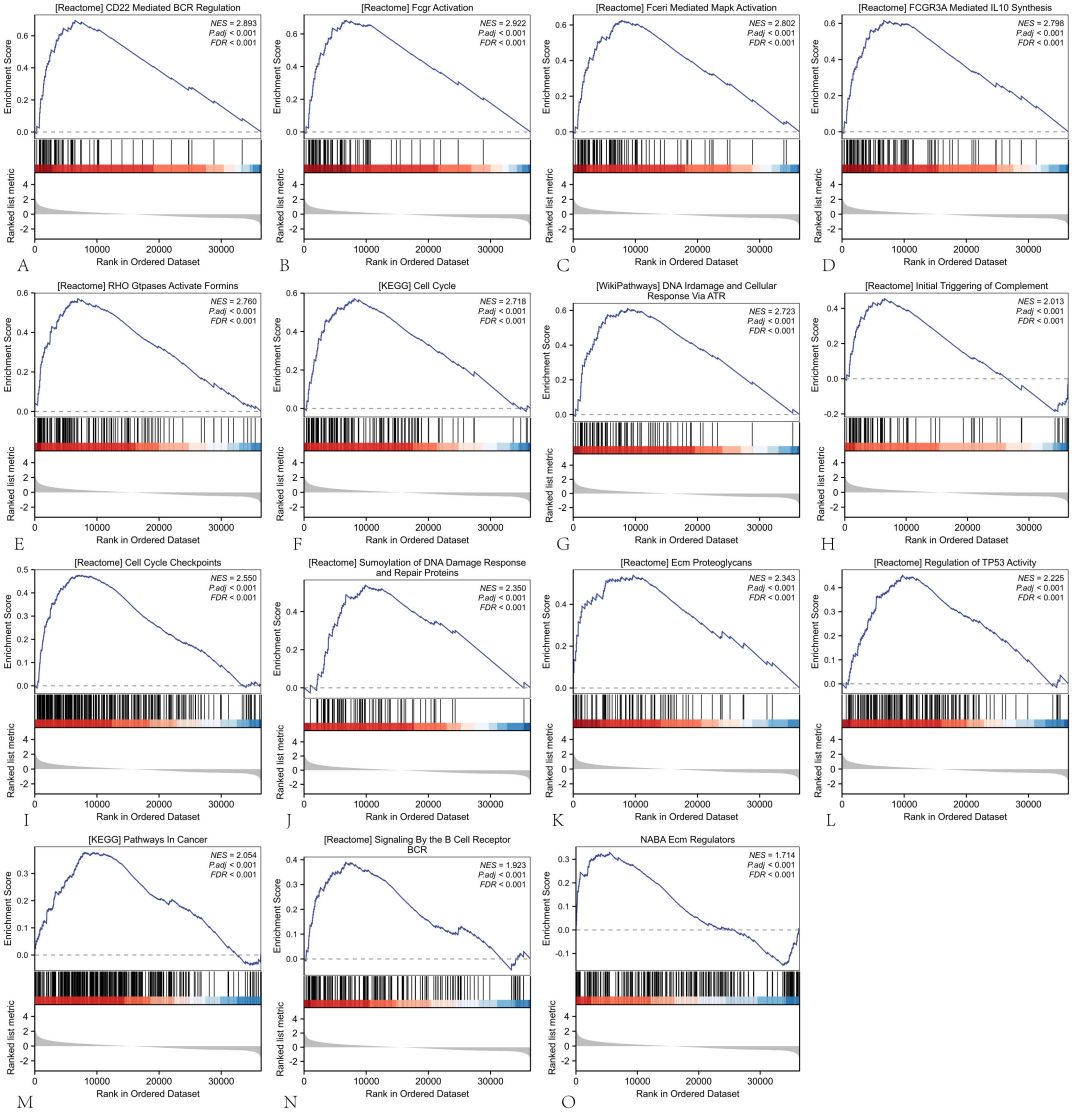
The GSEA software was employed to identify and analyze the potential signaling pathways associated with ARIH2 in HCC.

To elucidate the biological role of ARIH2 in HCC, GSEA was conducted between the ARIH2 low-expression group and the ARIH2 high-expression group. GSEA pathway analysis revealed that ARIH2 is primarily involved in CD22-mediated BCR regulation, FcγR activation, FcγRI-mediated MAPK activation, FcγRIIIA-mediated IL-10 synthesis, initial triggering of complement, DNA damage and cellular response via ATR, cell cycle regulation, ECM regulation, pathways in cancer, and regulation of TP53 activity and other relevant pathways(**Figure 10**). These findings suggest that ARIH2 may play a significant role in immune responses and tumor progression.

### ARIH2 expression is associated with immune cells and immune checkpoints

**Figure 11:**
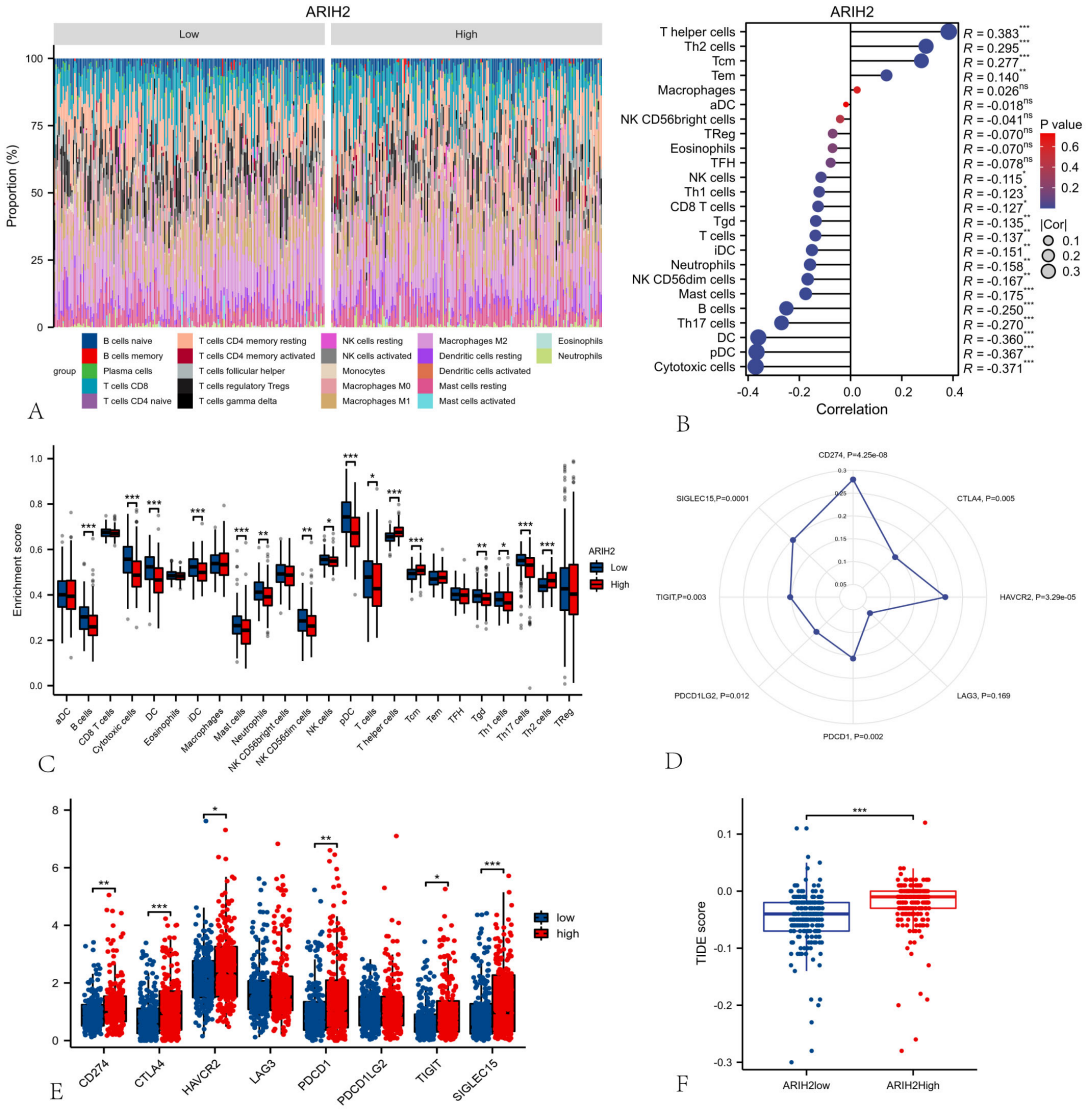
Relationships among ARIH2, Immune Cells and Immune Checkpoints. **(A)** The proportion of 22 immune cell types in HCC patients. **(B)** Correlation analysis between the expression levels of ARIH2 and 24 immune cell types using ssGSEA. **(C)** Comparative analysis of 24 immune cell enrichment scores between high and low ARIH2 expression groups. **(D)** Association analysis between the expression levels ARIH2 and eight immune checkpoint molecules. **(E)** Differential expression analysis of eight immune checkpoint molecules in high and low ARIH2 expression groups. **(F)** Comparative analysis of tumor immune dysfunction and exclusion (TIDE) scores in high and low ARIH2 expression groups.(*NS, p* > 0.05, *p < 0.05, **p < 0.01, ***p < 0.001).

To assess the composition of 22 immune cell types in HCC patients, we initially employed CIBERSORT analysis (**Figure 11A**). Subsequently, ssGSEA analysis were conducted to investigate the correlation between ARIH2 and 24 immune cell types(**Figure 11B**), as well as the differences in immune cell enrichment scores between high and low ARIH2 expression groups(**Figure 11C**). The results demonstrated that ARIH2 exhibited a positive correlation with T helper cells, TH2 cells, and central memory T cells (TCM), whereas it displayed a negative correlation with cytotoxic cells, dendritic cells (DC), TH17 cells, and B cells. These findings suggest that ARIH2 expression is linked to the degree of immune cell infiltration.

Immune checkpoints, which act as regulatory molecules that modulate the immune system, can inhibit T cell activation and promote T cell exhaustion, thereby facilitating tumor immune evasion. Immunotherapies targeting these checkpoints have demonstrated significant efficacy in various cancers (4, 19). We further evaluated the relationship between the expression levels of ARIH2 and the checkpoint levels of eight immune-related genes in HCC patients. Pearson’s correlation analysis (**Figure 11D**) revealed that six of these genes—CD274(PD-L1)、HAVCR2、PDCD1(PD-1) 、TIGIT、PDCD1LG2(PD-L2)and SIGLEC15—were positively correlated with the expression levels of ARIH2. We also compared the expression levels of immune gene checkpoints between the ARIH2 low-expression and high-expression groups. The results indicated that the expression levels of six immune gene checkpoints were significantly elevated in the ARIH2 high-expression group compared to the ARIH2 low-expression group (all p < 0.05, **Figure 11E**). Furthermore, we examined whether the response to immune checkpoint blockade (ICB) varied between the two groups. Our findings revealed that ICB scores were significantly higher in the ARIH2 high-expression group than in the ARIH2 low-expression group (p<0.001), suggesting that individuals with elevated ARIH2 expression exhibit a poorer response to immune checkpoint blockade (**Figure 11F**). Collectively, these results imply that ARIH2 may play a crucial role in tumor immune evasion and antitumor immunity in HCC pathogenesis.

**Figure 12:**
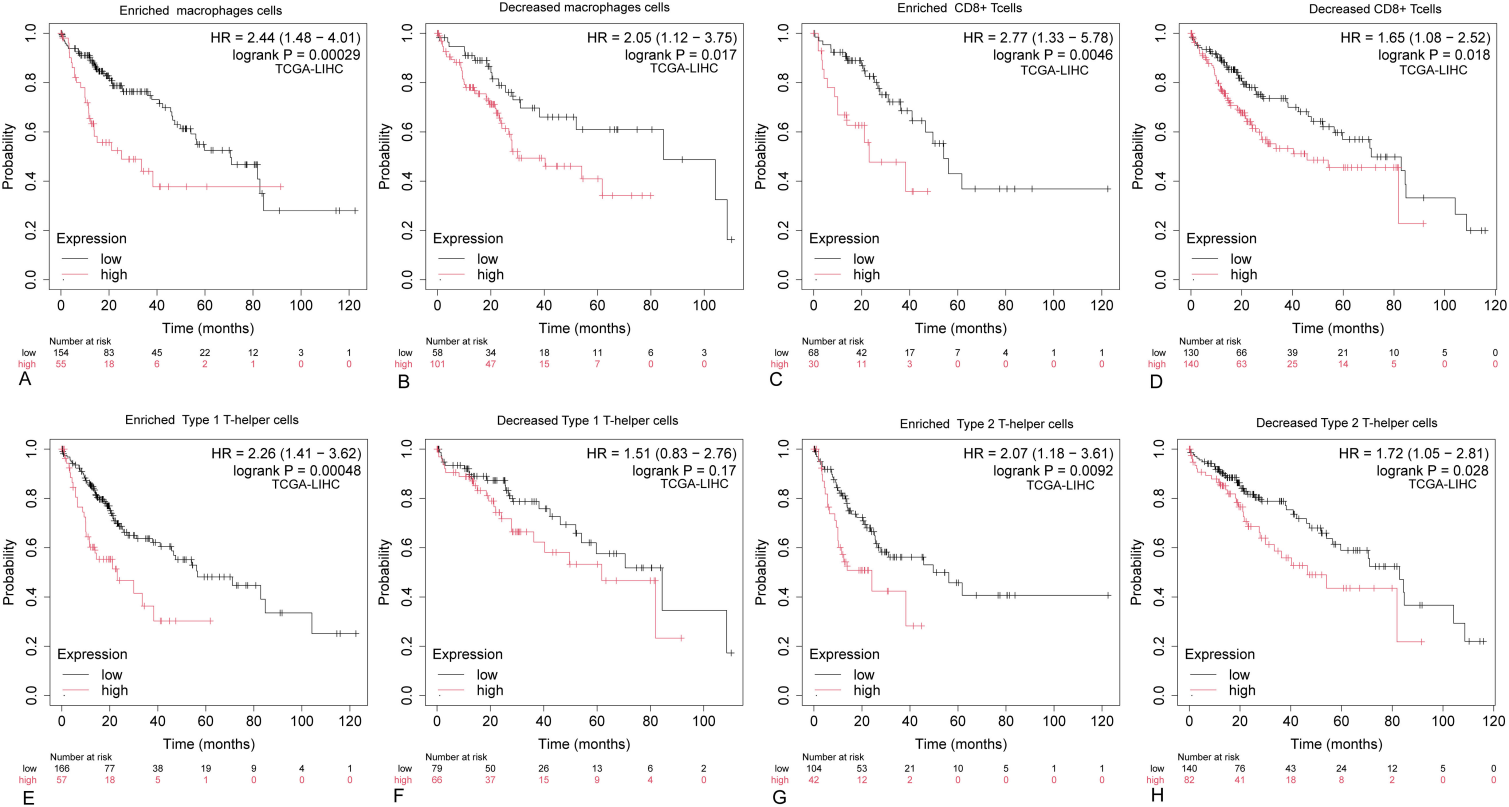
Kaplan-Meier survival curves for HCC immune cell subsets with high and low ARIH2 expression. **(A-H)** The Kaplan-Meier mapper was utilized to assess the relationship between the expression levels of ARIH2 in various immune cell subsets and overall survival in HCC patients.

The aforementioned findings indicate a significant correlation between ARIH2 expression and immune infiltration in HCC. Furthermore, elevated ARIH2 expression is linked to an unfavorable prognosis in HCC patients. Consequently, we hypothesize that ARIH2 may influence the prognosis of HCC patients, at least in part, by modulating immune cell infiltration. To further investigate this hypothesis, we conducted Kaplan-Meier (KM) plotter analyses of ARIH2 expression levels in macrophages, CD8+ T cells, TH1 cells, and TH2 cells. Our results demonstrate that HCC patients with high ARIH2 expression levels in these immune cell populations exhibit a poorer prognosis (**Figures 12A-H**). These observations support the notion that immune infiltration plays a role in the prognosis of HCC patients with elevated ARIH2 expression.

**Figure 13:**
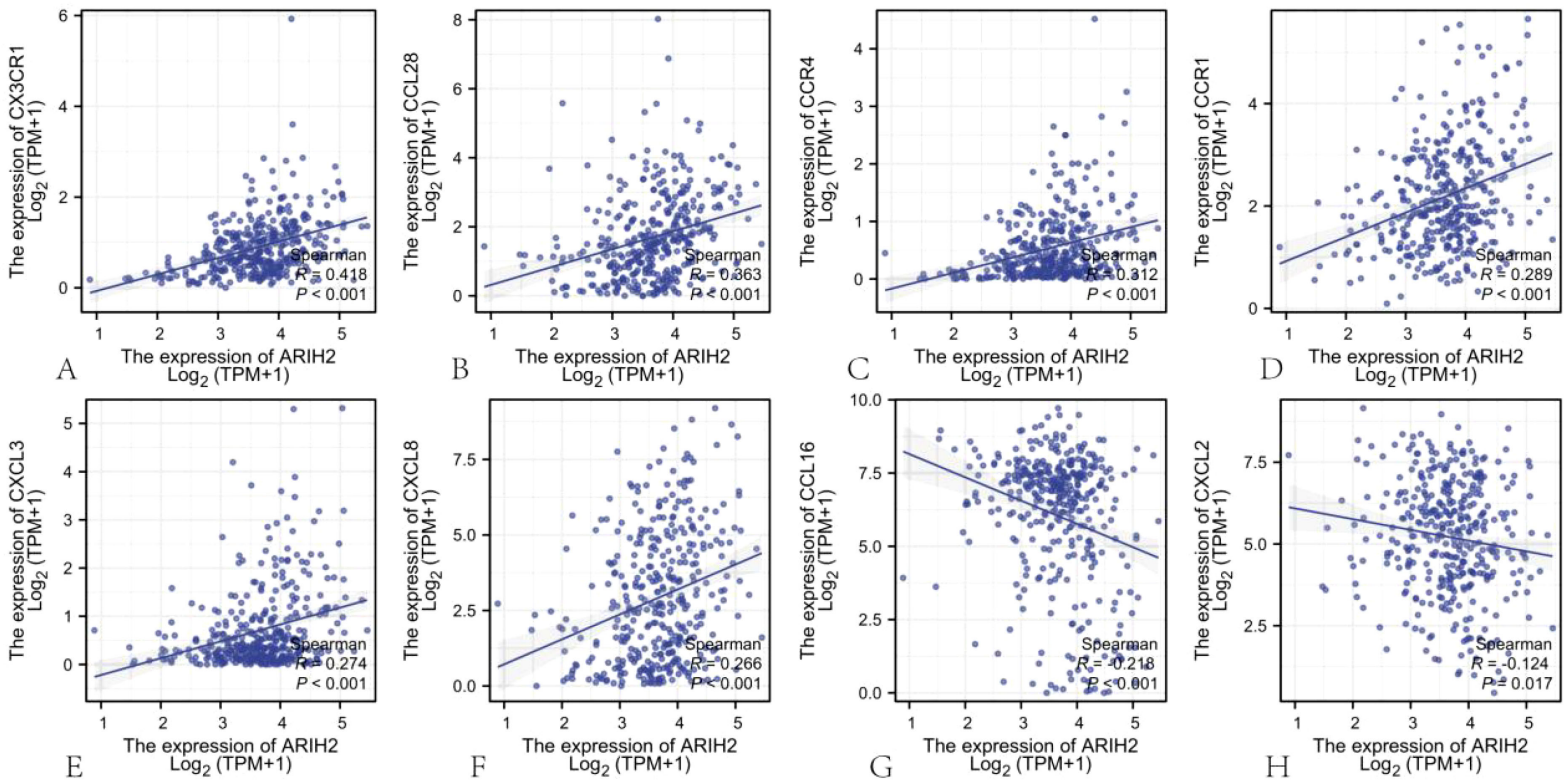
The correlation between ARIH2 expression levels and the levels of chemokines and their receptors.

Chemokines and chemokine receptors are crucial for the infiltration of immune cells into tumors. To further elucidate the role of ARIH2 in immune cell migration, this study investigated the correlation between ARIH2 expression levels and various chemokines and their receptors (20). The results demonstrated significant positive correlations between the expression levels of ARIH2 and CX3CR1 (r=0.418, P<0.001), CCL28 (r=0.363, P<0.001), CCR4 (r=0.312, P<0.001), CCR1 (r=0.289, P<0.001), CXCL3 (r=0.274, P<0.001) and CXCL8 (r=0.266, P<0.001) in HCC. Additionally, the expression levels of ARIH2 exhibited a significant negative correlations with CCL16 (r=-0.218, P<0.001), CXCL2 (r=-0.124, P=0.017)(**Figures13A-H**). These findings indicate that the expression levels of ARIH2 may influence the infiltration of specific immune cell types within the tumor microenvironment.

### The correlation between ARIH2 and ferroptosis in HCC

**Figure 14:**
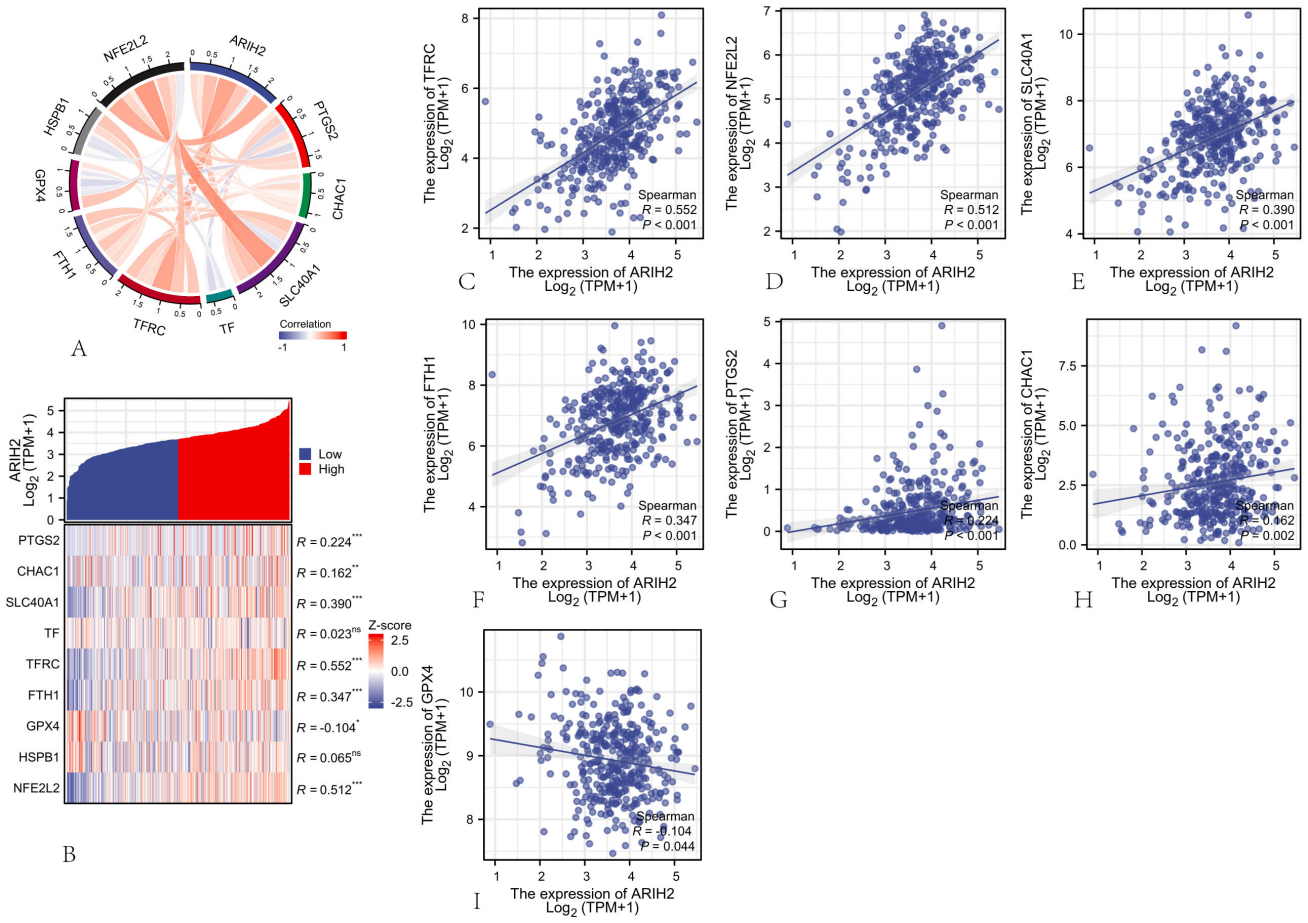
The Association between ARIH2 Expression and Ferroptosis-Related Gene Expression in HCC **(A)** Correlation Analysis of ARIH2 and Ferroptosis Markers Utilizing TCGA Data. **(B)** Differential Expression of Ferroptosis Markers in HCC Samples with High and Low ARIH2 Expression. **(C-I)** Correlation Analysis of ARIH2 and Ferroptosis Markers Utilizing GEPIA2 Data.(*NS, p* > 0.05, *p < 0.05, **p < 0.01, ***p < 0.001).

Ferroptosis, initially characterized in 2012 (21), represents an iron-dependent form of regulated cell death distinct from apoptosis and autophagy, primarily driven by the accumulation of reactive oxygen species (ROS). This process is marked by specific morphological alterations in mitochondria, such as cristae reduction, outer membrane rupture, and membrane density increase (22). Ferroptosis is governed by multiple genetic and signaling pathways implicated in carcinogenesis, indicating that inducing ferroptosis might serve as a therapeutic strategy to impede cancer progression. In this paper, Spearman correlation analysis was employed to evaluate the relationship between ARIH2 expression levels and ferroptosis-associated genes.The findings revealed a statistically significant correlation between ARIH2 expression and markers of ferroptosis, specifically PTGS2, CHAC1, SLC40A1, TFRC, FTH1, GPX4, and NFE2L2 (**Figure14A-B**). Patients were categorized into high and low ARIH2 expression groups based on the median value, and differentially expressed ferroptosis-related genes were identified(**Figure14C-I**). Notably, the expression levels of PTGS2, CHAC1, SLC40A1, TFRC, FTH1, and NFE2L2 were significantly elevated (P<0.05), whereas GPX4 expression was markedly reduced (P<0.05) in the high ARIH2 expression group. These results suggest that ARIH2 may play a crucial role in modulating ferroptosis, thereby influencing the progression and prognosis of HCC.

### Sensitivity analysis of anticancer drugs

**Figure 15:**
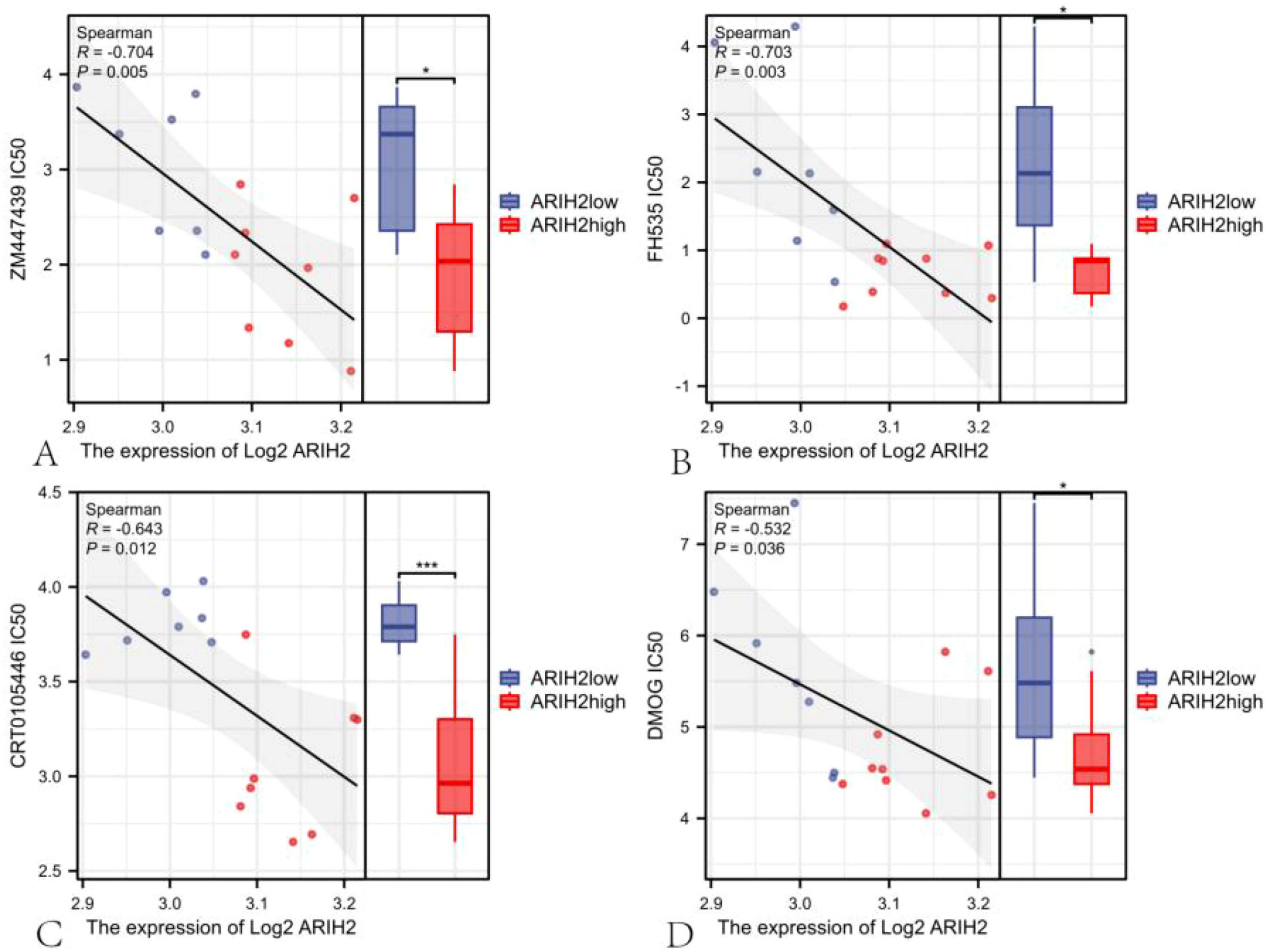
The scatter plot of the correlation between the expression of ARIH2 and the IC50 of drug.

To further investigate the relationship between ARIH2 expression and drug sensitivity, we conducted an analysis of the response data for 357 anticancer drugs. Our results demonstrated a negative correlation between the expression of ARIH2 and the IC50 of anticancer drugs, including ZM447439 (R = -0.704; P = 0.005), Methotrexate (R = -0.706; P = 0.013), FH535 (R = -0.703; P = 0.003), Fulvestrant (R = -0.671; P = 0.008), CRT0105446 (R = -0.643; P = 0.012), Palbociclib (R = -0.574; P = 0.034), DMOG (R = -0.532; P = 0.036), SB590885 (R = -0.557; P = 0.034), Olaparib (R = -0.507; P = 0.004), and Cisplatin (R = -0.448; P = 0.006) (**Supplementary Figure 2**). As illustrated in **Figure 15**, there were significant differences in the IC50 of ZM447439, FH535, CRT0150446, and DMOG between the ARIH2 high-expression and low-expression groups. In samples with high ARIH2 expression, the therapeutic effects of these anticancer drugs were more pronounced. These findings suggest that for HCC patients with elevated ARIH2 expression levels, the identified anticancer drugs may offer clinical benefits to some extent.

### ARIH2 regulates the proliferation and migration of HCC

**Figure 16:**
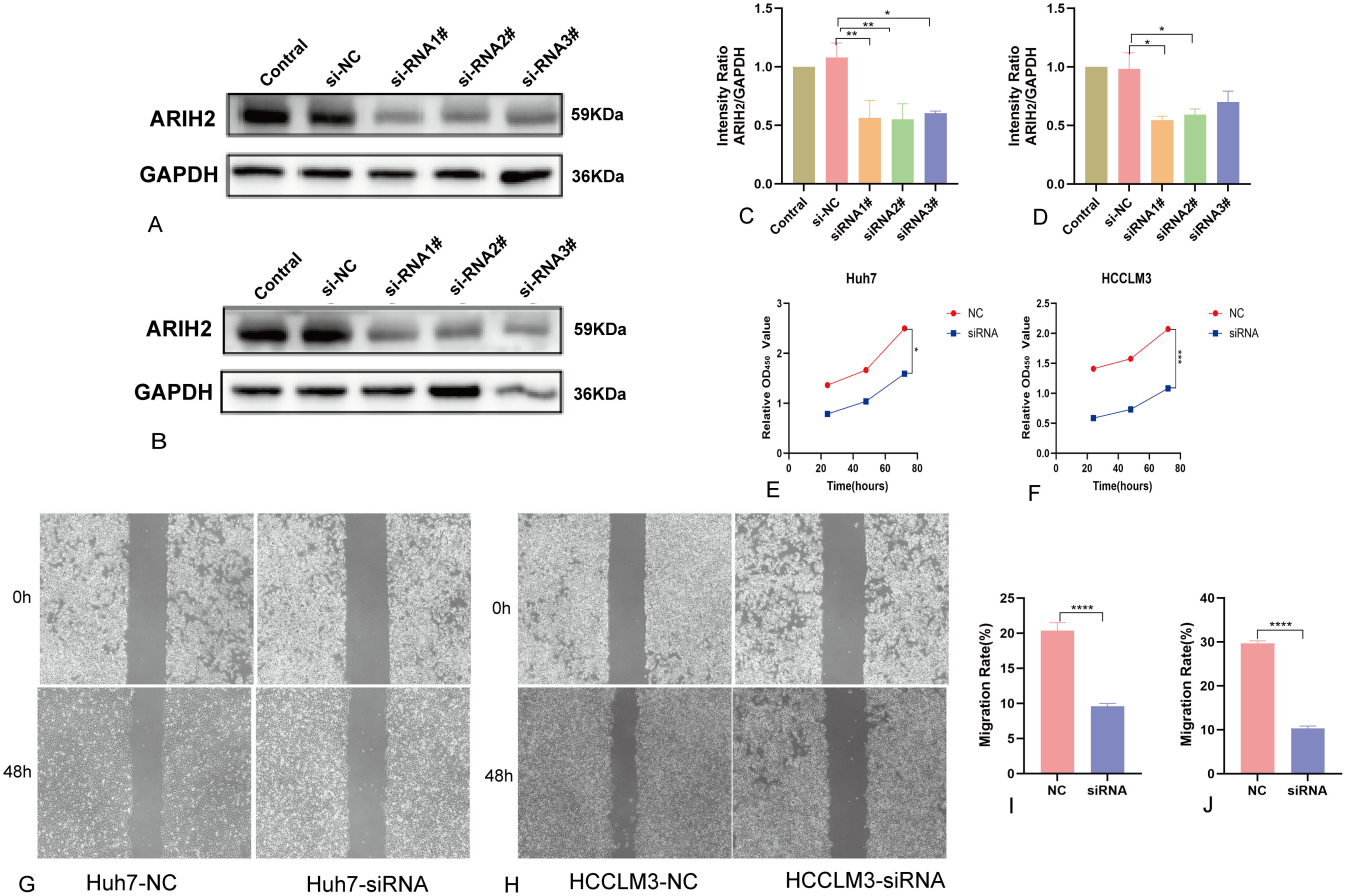
ARIH2 inhibits significantly the proliferation, migration and invasion of HCC cells. **(A,C)** Western blot confirmed the downexpression of ARIH2 in Huh7 cells. **(B,D)** Western blot confirmed the downexpression of ARIH2 in HCCLM3 cells. **(E)** The CCK-8 assay was employed to assess the proliferative activity of Huh7 cells. **(F)** The CCK-8 assay was employed to assess the proliferative activity of HCCLM3 cells. **(G,I)** The wound healing assay was utilized to measure the migratory capacity of Huh7 cells. **(H,J)** The wound healing assay was utilized to measure the migratory capacity of HCCLM3 cells. (*p < 0.05, **p < 0.01, ***p < 0.001, ****p < 0.0001).

To further assess the role of ARIH2, we conducted knockdown experiments in Huh7 and HCCLM3 cell and confirmed the efficiency of knockdown using Western blot (**Figures 16A–D**). The CCK-8 assay revealed a significant reduction in cell viability following ARIH2 knockdown (**Figures 16E, F**). Cell migration was assessed via wound healing assays, which demonstrated that the migratory capacity of both Huh7 and HCCLM3 cells was markedly diminished after ARIH2 knockdown (**Figures 16G-J**). Collectively, these findings suggest that ARIH2 plays a pivotal role in regulating the proliferation and migration of HCC.

## Discussion

ARIH2, a member of the Ariadne subfamily, is a gene that encodes the Triad1 protein, characterized by two RING finger domains and possessing E3 ubiquitin ligase activity. This protein plays a crucial role in post-translational modifications involved in various cellular processes. Recent studies have investigated the regulatory function of ARIH2 in multiple malignant cancers. Evidence indicates that ARIH2 significantly influences the development and progression of gastric cancer, acute myeloid leukemia, human non-small cell lung cancer, and other malignancies (7–9). Nevertheless, there is limited information regarding ARIH2 roles within HCC. Therefore, this investigation conducted a comprehensive bioinformatics-based analysis to explore its possible functional and diagnostic roles in this context.

In this study, we investigated the mRNA expression levels of ARIH2 across various tumor types and their corresponding adjacent normal tissues using the TCGA and GTEx databases. The findings indicate that ARIH2 is upregulated in a variety of human cancers, particularly in LIHC. Additionally, our data reveal that both mRNA and protein expressions of ARIH2 are elevated in HCC tissues and are associated with adverse clinicopathological characteristics, such as pathologic stage, TNM stage, histologic grade, AFP levels, and vascular invasion. ROC curve analysis suggests that ARIH2 has the potential to serve as a diagnostic biomarker for distinguishing HCC tissues from normal tissues. Our *in vitro* experiments further confirmed the overexpression of ARIH2 in HCC tissues and cell lines, supporting its role in HCC progression. KM curve analysis revealed a significant association between ARIH2 expression and OS, DSS and PFS of HCC patients in the TCGA dataset. Consistent results were obtained by analyzing the correlation between ARIH2 expression and OS in HCC patients utilizing the ICGC, GEPIA2 and KM Plotter databases. Multivariate Cox regression analysis identified ARIH2 as the sole statistically significant parameter associated with overall survival of HCC, indicating its status as an independent predictor of HCC survival. A nomogram was established for clinical prognosis prediction based on the results of multivariate Cox regression, and the accuracy of this model was validated. Calibration plots demonstrated a favorable alignment between actual and predicted OS values at 1, 3, and 5 years. Consequently, this nomogram may emerge as a novel and valuable tool for prognostic prediction, suggesting its potential utility as a biomarker for providing critical information for early diagnosis and treatment decision-making.

Previous studies have reported that ARIH2 interacts with NLRP3 through the NACHT domain, thereby mediating NLRP3 ubiquitination (9). Overexpression of ARIH2 has been shown to inhibit p53 degradation mediated by the E3 ligase MDM2 (23). Additionally, Arih2 regulates Hedgehog signaling by facilitating Smo ubiquitination and endoplasmic reticulum-related degradation (24). ARIH2 also decreases p21 stability via ubiquitination, influencing DNA damage and apoptosis in gastric cancer (GC) cells (7). To elucidate the molecular mechanisms associated with ARIH2 in HCC progression, functional enrichment analysis was conducted on 2471 differentially expressed genes. Further investigation into the potential role of ARIH2 in HCC through GO) and KEGG enrichment analyses indicates its involvement in immune system pathways and ion channel activity. GSEA confirmed that ARIH2 is significantly associated with pathways such as CD22-mediated BCR regulation, FcγR activation, FcγRI-mediated MAPK activation, FcγRIIIA-mediated IL-10 synthesis, initial triggering of complement, DNA damage and cellular response via ATR, cell cycle regulation, ECM regulation, pathways in cancer, and regulation of TP53 activity and other relevant pathways, suggesting that ARIH2 may play a significant role in immune and tumor progression pathways.

The tumor microenvironment (TME) comprises not only tumor cells but also a diverse array of immune and stromal cells (25). The type and degree of immune cell infiltration can serve as a predictive marker for patient response to immunotherapy. In this study, we observed an inverse relationship between the expression levels of ARIH2 and the abundance of cytotoxic cells and TH17 cells in HCC samples. Cytotoxic cells play a crucial role in activating the immune response against tumors, resulting in tumor cell lysis (26), whereas TH17 cells facilitate the recruitment of TH1 cells and enhance immune activation (27). Conversely, the expression levels of ARIH2 exhibited a positive correlation with T helper cells and TH2 cells. Modulation of T helper cells within the TME has been linked to the expression of the chemokine receptor CCR8 (28). Furthermore, cytokines secreted by TH2 cells, including IL-4 and IL-13, stimulate lung mesenchymal stromal cells to upregulate C3 expression, which promotes neutrophil recruitment and the formation of extracellular traps, ultimately contributing to tumor metastasis (29). Our findings indicate that the expression levels of ARIH2 is associated with the concentrations of multiple chemokines and chemokine receptors in HCC tissues, suggesting its potential influence on the TME composition via diverse mechanisms. As regulatory molecules that modulate the immune system, immune checkpoints suppress T cell activation and facilitate T cell exhaustion, resulting in tumor immune evasion, and immunotherapies targeting these checkpoints have demonstrated promising outcomes in various cancers (4, 19). Additionally, our study has validated that the expression level of ARIH2 exhibits a significant positive correlation with the expression levels of immune checkpoint genes, including CD274 (PD-L1), HAVCR2, PDCD1 (PD-1), TIGIT, PDCD1LG2 (PD-L2), and SIGLEC15 in HCC. These findings indicate that elevated ARIH2 levels are intricately linked to mechanisms that enhance immune evasion by HCC tumor cells, thus contributing to tumor growth and progression.

Ferroptosis targeting has been proposed as a therapeutic strategy for cancer, particularly for refractory tumors (21). Numerous tumor suppressor gene proteins, including p53, fumarase and BAP1, have been demonstrated to sensitize tumor cells to ferroptosis (22). Recent investigations have demonstrated that ferroptosis plays a pivotal role in tumor progression, particularly HCC (30). Specifically, inducing ferroptosis not only inhibits the proliferation of liver cancer cells, thereby preventing tumor development, but also enhances the efficacy of immunotherapy and augments the anti-tumor immune response (30). In this study, we examined the relationship between ARIH2 expression levels and genes associated with ferroptosis, revealing a positive correlation between them. These findings suggest that ARIH2 may promote tumorigenesis by modulating ferroptosis, potentially providing a novel approach for targeting ferroptosis in HCC therapy.

This study enhances our comprehension of the relationship between the expression levels of ARIH2 and HCC progression. However, several limitations exist. Firstly, while we have investigated the association between ARIH2 and immune infiltration as well as ferroptosis in HCC patients, functional experiments to confirm the role of ARIH2 in modulating the HCC tumor microenvironment are absent. Secondly, our findings indicate that the knockdown of ARIH2 suppresses cell proliferation and migration in HCC. However, further studies are necessary to elucidate the underlying molecular mechanisms by which ARIH2 influences cancer progression.

### Conclusion

Research has demonstrated that ARIH2 expression is elevated in HCC tissue samples, exhibiting significant correlations with the clinical stage, histopathological grade, and tumor characteristics of HCC. Additionally, increased ARIH2 levels are associated with a reduced survival rate among HCC patients. These findings indicate that ARIH2 may serve as a biomarker for the diagnosis and prognosis of HCC, as well as a potential therapeutic target.

## Data Availability

The original contributions presented in the study are included in the article/**Supplementary Material**. Further inquiries can be directed to the corresponding author.

## References

[1] Bray F Laversanne M Sung H Ferlay J Siegel RL Soerjomataram I. Global cancer statistics 2022: GLOBOCAN estimates of incidence and mortality worldwide for 36 cancers in 185 countries. CA Cancer J Clin. (2024) 74:229–63. doi: 10.3322/caac.21834 38572751

[2] Vogel A Meyer T Sapisochin G Salem R Saborowski A. Hepatocellular carcinoma. Lancet. (2022) 400:1345–62. doi: 10.1016/S0140-6736(22)01200-4 36084663

[3] Sangro B Sarobe P Hervás-Stubbs S Melero I. Advances in immunotherapy for hepatocellular carcinoma. Nat Rev Gastroenterol Hepatol. (2021) 18:525–43. doi: 10.1038/s41575-021-00438-0 PMC8042636 33850328

[4] Shen KY Zhu Y Sz X Qin LX. Immunosuppressive tumor microenvironment and immunotherapy of hepatocellular carcinoma: current status and prospectives. J Hematol Oncol. (2024) 17:25. doi: 10.1186/s13045-024-01549-2 38679698 PMC11057182

[5] Yu B Ma W. Biomarker discovery in hepatocellular carcinoma (HCC) for personalized treatment and enhanced prognosis. Cytokine Growth Factor Rev. (2024) 79:29–38. doi: 10.1016/j.cytogfr.2024.08.006 39191624

[6] Wang P Dai X Jiang W Li Y Wei W. RBR E3 ubiquitin ligases in tumorigenesis. Semin Cancer Biol. (2020) 67:131–44. doi: 10.1016/j.semcancer.2020.05.002 32442483

[7] Geng S Peng W Wang X Hu X Liang H Hou J. ARIH2 regulates the proliferation, DNA damage and chemosensitivity of gastric cancer cells by reducing the stability of p21 via ubiquitination. Cell Death Dis. (2022) 13:564. doi: 10.1038/s41419-022-04965-9 35732617 PMC9218151

[8] Wang H Bei L Shah CA Huang W Platanias LC Eklund EA. The E3 ubiquitin ligase Triad1 influences development of Mll-Ell-induced acute myeloid leukemia. Oncogene. (2018) 37:2532–44. doi: 10.1038/s41388-018-0131-5 PMC5945580 29459712

[9] Kawashima A Karasawa T Tago K Kimura H Kamata R Usui-Kawanishi F. ARIH2 ubiquitinates NLRP3 and negatively regulates NLRP3 inflammasome activation in macrophages. J Immunol. (2017) 199:3614–22. doi: 10.4049/jimmunol.1700184 29021376

[10] Zeng H Castillo-Cabrera J Manser M Lu B Yang Z Strande V. Genome-wide CRISPR screening reveals genetic modifiers of mutant EGFR dependence in human NSCLC. Elife. (2019) 8:e50223. doi: 10.7554/eLife.50223 31741433 PMC6927754

[11] Hüttenhain R Xu J Burton LA Gordon DE Hultquist JF Johnson JR. ARIH2 is a vif-dependent regulator of CUL5-mediated APOBEC3G degradation in HIV infection. Cell Host Microbe. (2019) 26:86–99.e7. doi: 10.1016/j.chom.2019.05.008 31253590 PMC7153695

[12] Lin AE Ebert G Ow Y Preston SP Toe JG Cooney JP. ARIH2 is essential for embryogenesis, and its hematopoietic deficiency causes lethal activation of the immune system. Nat Immunol. (2013) 14:27–33. doi: 10.1038/ni.2478 23179078

[13] Vivian J Rao AA Nothaft FA Ketchum C Armstrong J Novak A. Toil enables reproducible, open source, big biomedical data analyses. Nat Biotechnol. (2017) 35:314–6. doi: 10.1038/nbt.3772 PMC5546205 28398314

[14] Subramanian A Tamayo P Mootha VK Mukherjee S Ebert BL Gillette MA. Gene set enrichment analysis: a knowledge-based approach for interpreting genome-wide expression profiles. Proc Natl Acad Sci U S A. (2005) 102:15545–50. doi: 10.1073/pnas.0506580102 PMC1239896 16199517

[15] Newman AM Liu CL Green MR Gentles AJ Feng W Xu Y. Robust enumeration of cell subsets from tissue expression profiles. Nat Methods. (2015) 12:453–7. doi: 10.1038/nmeth.3337 PMC4739640 25822800

[16] Bindea G Mlecnik B Tosolini M Kirilovsky A Waldner M Obenauf AC. Spatiotemporal dynamics of intratumoral immune cells reveal the immune landscape in human cancer. Immunity. (2013) 39:782–95. doi: 10.1016/j.immuni.2013.10.003 24138885

[17] Jiang P Gu S Pan D Fu J Sahu A Hu X. Signatures of T cell dysfunction and exclusion predict cancer immunotherapy response. Nat Med. (2018) 24:1550–8. doi: 10.1038/s41591-018-0136-1 PMC6487502 30127393

[18] Geeleher P Cox NJ Huang RS. Clinical drug response can be predicted using baseline gene expression levels and *in vitro* drug sensitivity in cell lines. Genome Biol. (2014) 15:R47. doi: 10.1186/gb-2014-15-3-r47 24580837 PMC4054092

[19] Oura K Morishita A Tani J Masaki T. Tumor immune microenvironment and immunosuppressive therapy in hepatocellular carcinoma: A review. Int J Mol Sci. (2021) 22:5801. doi: 10.3390/ijms22115801 34071550 PMC8198390

[20] He C He L Lu Q Xiao J Dong W. The functions and prognostic values of chemokine and chemokine receptors in gastric cancer. Am J Cancer Res. (2022) 12:3034–50. PMC9360243 35968351

[21] Dixon SJ Lemberg KM Lamprecht MR Xiao J Dong W. Ferroptosis: an iron-dependent form of nonapoptotic cell death. Cell. (2012) 149:1060–72. doi: 10.1016/j.cell.2012.03.042 PMC3367386 22632970

[22] Mou Y Wang J Wu J Skouta R Zaitsev EM Gleason CE. Ferroptosis, a new form of cell death: opportunities and challenges in cancer. J Hematol Oncol. (2019) 12:34. doi: 10.1186/s13045-019-0720-y 30925886 PMC6441206

[23] Bae S Jung JH Kim K He D Zhang C Duan C. TRIAD1 inhibits MDM2-mediated p53 ubiquitination and degradation. FEBS Lett. (2012) 586:3057–63. doi: 10.1016/j.febslet.2012.07.022 22819825

[24] Lv B Zhang XO Pazour GJ. Arih2 regulates Hedgehog signaling through smoothened ubiquitylation and ER-associated degradation. J Cell Sci. (2022) 135:jcs260299. doi: 10.1242/jcs.260299 35899529 PMC9481925

[25] Hinshaw DC Shevde LA. The tumor microenvironment innately modulates cancer progression. Cancer Res. (2019) 79:4557–66. doi: 10.1158/0008-5472.CAN-18-3962 PMC6744958 31350295

[26] Banerjee A Chabria Y Kanna NRR Gopi J Rowlo P Sun XF. Role of tumor specific niche in colon cancer progression and emerging therapies by targeting tumor microenvironment. Adv Exp Med Biol. (2021) 1341:177–92. doi: 10.1007/5584_2019_355 30969400

[27] Cachot A Bilous M Liu YC Li X Saillard M Cenerenti M. Tumor-specific cytolytic CD4 T cells mediate immunity against human cancer. Sci Adv. (2021). doi: 10.1126/sciadv.abe3348 PMC7909889 33637530

[28] Fraga M Yáñez M Sherman M Llerena F Hernandez M Nourdin G. Immunomodulation of T helper cells by tumor microenvironment in oral cancer is associated with CCR8 expression and rapid membrane vitamin D signaling pathway. Front Immunol. (2021) 12:643298. doi: 10.3389/fimmu.2021.643298 34025655 PMC8137990

[29] Zheng Z Li YN Jia S Zhu M Cao L Tao M. Lung mesenchymal stromal cells influenced by Th2 cytokines mobilize neutrophils and facilitate metastasis by producing complement C3. Nat Commun. (2021) 12:6202. doi: 10.1038/s41467-021-26460-z 34707103 PMC8551331

[30] Zhu X Sha X Zang Y Ren Q Zhang S Ma D. Current progress of ferroptosis study in hepatocellular carcinoma. Int J Biol Sci. (2024) 20:3621–37. doi: 10.7150/ijbs.96014 PMC11234204 38993573

