# Supplementary material for: ARIH2 serves as a potential prognostic biomarker for hepatocellular carcinoma associated with immune infiltration and ferroptosis
Source: Front Immunol. 2025 Apr 7;16:1548691. doi: 10.3389/fimmu.2025.1548691 (PMC12009847; doi:10.3389/fimmu.2025.1548691)
Supplement: Supplementary file 1 [file DataSheet1.zip › Raw data file/IHC scores.docx]

**Supplemental table 1. IHC scores of Cancerous tissues and paracancerous tissues in 12 HCC patients**

| Patient ID | IHC scores | |
| --- | --- | --- |
|  | Cancerous tissues | Paracancerous tissues |
| 1 | 3 | 1 |
| 2 | 3 | 2 |
| 3 | 3 | 1 |
| 4 | 3 | 3 |
| 5 | 2 | 2 |
| 6 | 3 | 2 |
| 7 | 3 | 1 |
| 8 | 3 | 2 |
| 9 | 3 | 2 |
| 10 | 3 | 1 |
| 11 | 3 | 2 |
| 12 | 3 | 2 |
