# Supplementary material for: ARIH2 serves as a potential prognostic biomarker for hepatocellular carcinoma associated with immune infiltration and ferroptosis
Source: Front Immunol. 2025 Apr 7;16:1548691. doi: 10.3389/fimmu.2025.1548691 (PMC12009847; doi:10.3389/fimmu.2025.1548691)
Supplement: Supplementary file 1 [file DataSheet1.zip › Raw data file/Supplementary Table 1.docx]

Clinical Baseline Analysis of the Association Between ARIH2 Expression and Diverse Clinicopathological Characteristics of HCC Patients in the TCGA Database

| Characteristics | Low expression of ARIH2 | High expression of ARIH2 | P value |
| --- | --- | --- | --- |
| n | 187 | 187 |  |
| Age, n (%) |  |  | 0.234 |
| <= 60 | 83 (22.3%) | 94 (25.2%) |  |
| > 60 | 104 (27.9%) | 92 (24.7%) |  |
| Gender, n (%) |  |  | 0.912 |
| Female | 60 (16%) | 61 (16.3%) |  |
| Male | 127 (34%) | 126 (33.7%) |  |
| Race, n (%) |  |  | 0.728 |
| Asian | 78 (21.5%) | 82 (22.7%) |  |
| Black or African American | 7 (1.9%) | 10 (2.8%) |  |
| White | 94 (26%) | 91 (25.1%) |  |
| BMI, n (%) |  |  | 0.466 |
| <= 25 | 87 (25.8%) | 90 (26.7%) |  |
| > 25 | 85 (25.2%) | 75 (22.3%) |  |
| AFP(ng/ml), n (%) |  |  | 0.003 |
| <= 400 | 124 (44.3%) | 91 (32.5%) |  |
| > 400 | 24 (8.6%) | 41 (14.6%) |  |
| Albumin(g/dl), n (%) |  |  | 0.451 |
| < 3.5 | 40 (13.3%) | 29 (9.7%) |  |
| >= 3.5 | 122 (40.7%) | 109 (36.3%) |  |
| Adjacent hepatic tissue inflammation, n (%) |  |  | 0.841 |
| None | 64 (27%) | 54 (22.8%) |  |
| Mild&Severe | 63 (26.6%) | 56 (23.6%) |  |
| Child-Pugh grade, n (%) |  |  | 0.551 |
| A | 124 (51.5%) | 95 (39.4%) |  |
| B&C | 11 (4.6%) | 11 (4.6%) |  |
| Pathologic stage, n (%) |  |  | 0.004 |
| Stage I&Stage II | 144 (41.1%) | 116 (33.1%) |  |
| Stage III&Stage IV | 34 (9.7%) | 56 (16%) |  |
| Pathologic T stage, n (%) |  |  | 0.008 |
| T1&T2 | 149 (40.2%) | 129 (34.8%) |  |
| T3&T4 | 35 (9.4%) | 58 (15.6%) |  |
| Pathologic N stage, n (%) |  |  | 0.716 |
| N0 | 119 (46.1%) | 135 (52.3%) |  |
| N1 | 1 (0.4%) | 3 (1.2%) |  |
| Pathologic M stage, n (%) |  |  | 0.604 |
| M0 | 132 (48.5%) | 136 (50%) |  |
| M1 | 3 (1.1%) | 1 (0.4%) |  |
| Tumor status, n (%) |  |  | 0.098 |
| Tumor free | 109 (30.7%) | 93 (26.2%) |  |
| With tumor | 69 (19.4%) | 84 (23.7%) |  |
| Vascular invasion, n (%) |  |  | 0.778 |
| No | 110 (34.6%) | 98 (30.8%) |  |
| Yes | 60 (18.9%) | 50 (15.7%) |  |
| Residual tumor, n (%) |  |  | 0.889 |
| R0 | 169 (49%) | 158 (45.8%) |  |
| R1&R2 | 9 (2.6%) | 9 (2.6%) |  |
| Histologic grade, n (%) |  |  | 0.006 |
| G1&G2 | 129 (35%) | 104 (28.2%) |  |
| G3&G4 | 55 (14.9%) | 81 (22%) |  |
