# Supplementary figures and images for: ARIH2 serves as a potential prognostic biomarker for hepatocellular carcinoma associated with immune infiltration and ferroptosis
Source: Front Immunol. 2025 Apr 7;16:1548691. doi: 10.3389/fimmu.2025.1548691 (PMC12009847; doi:10.3389/fimmu.2025.1548691)

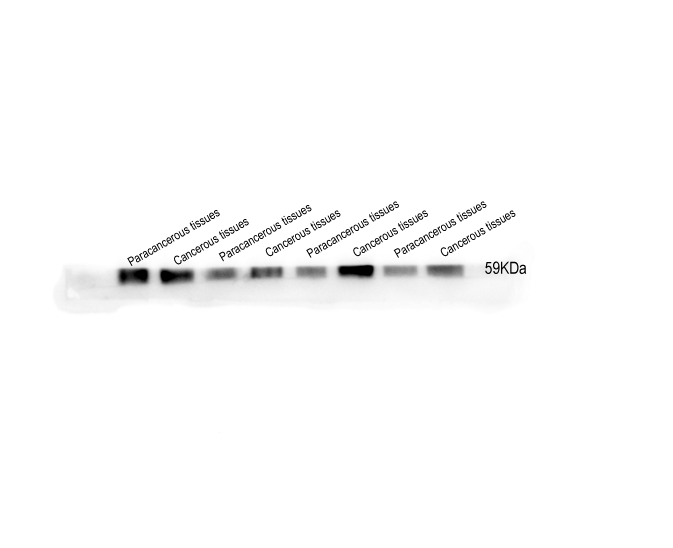

Supplement: Supplementary file 1 [file DataSheet1.zip › Raw data file/ARIH2 1.tif]

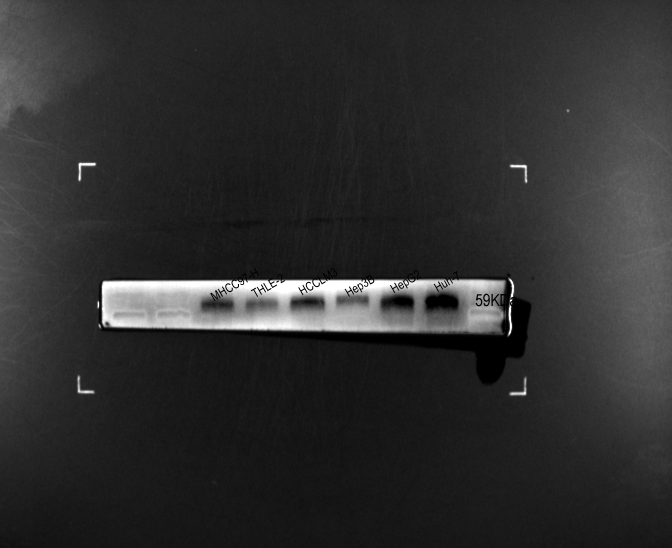

Supplement: Supplementary file 1 [file DataSheet1.zip › Raw data file/ARIH2.tif]

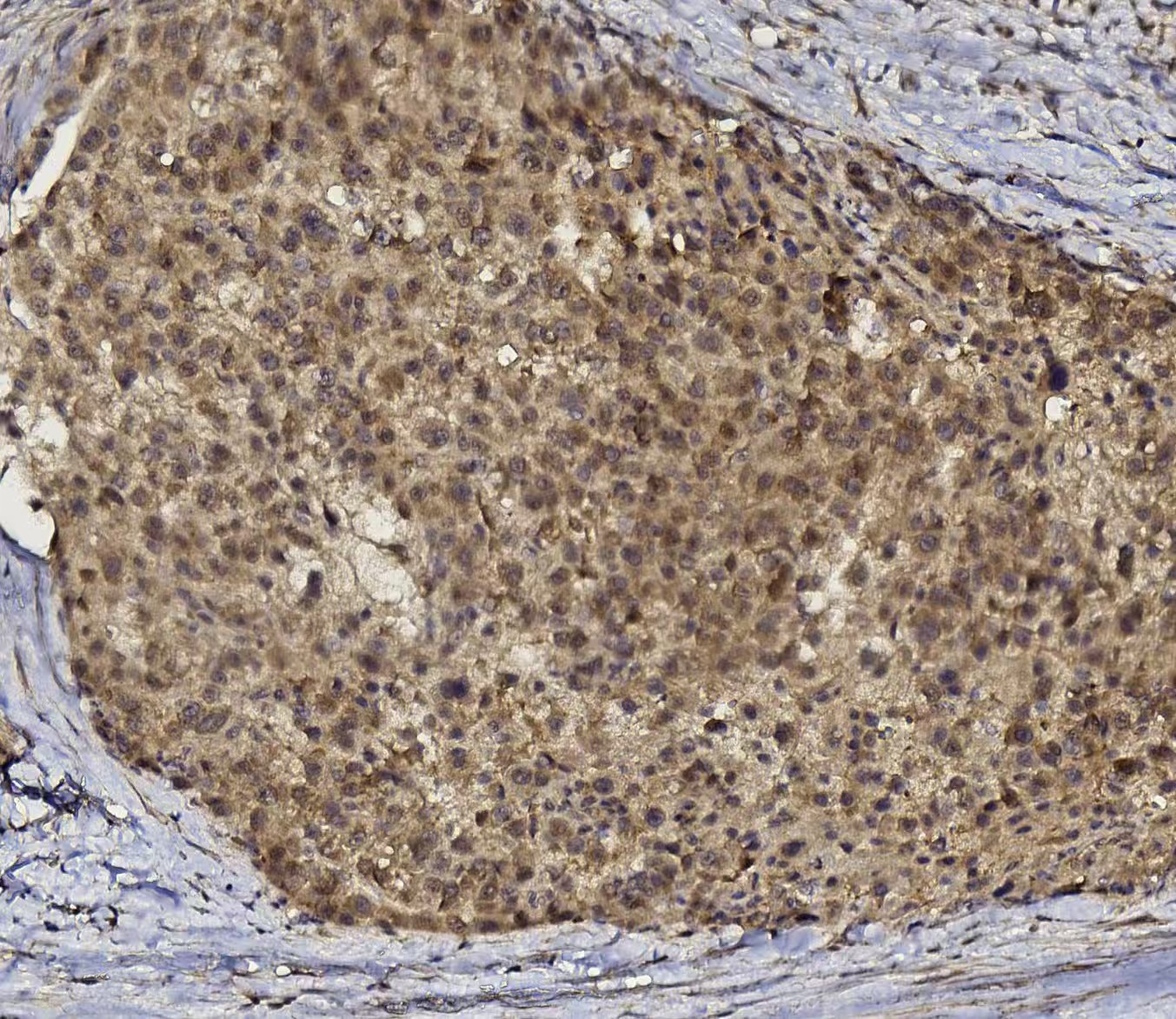

Supplement: Supplementary file 1 [file DataSheet1.zip › Raw data file/Cancerous tissues 1.jpg]

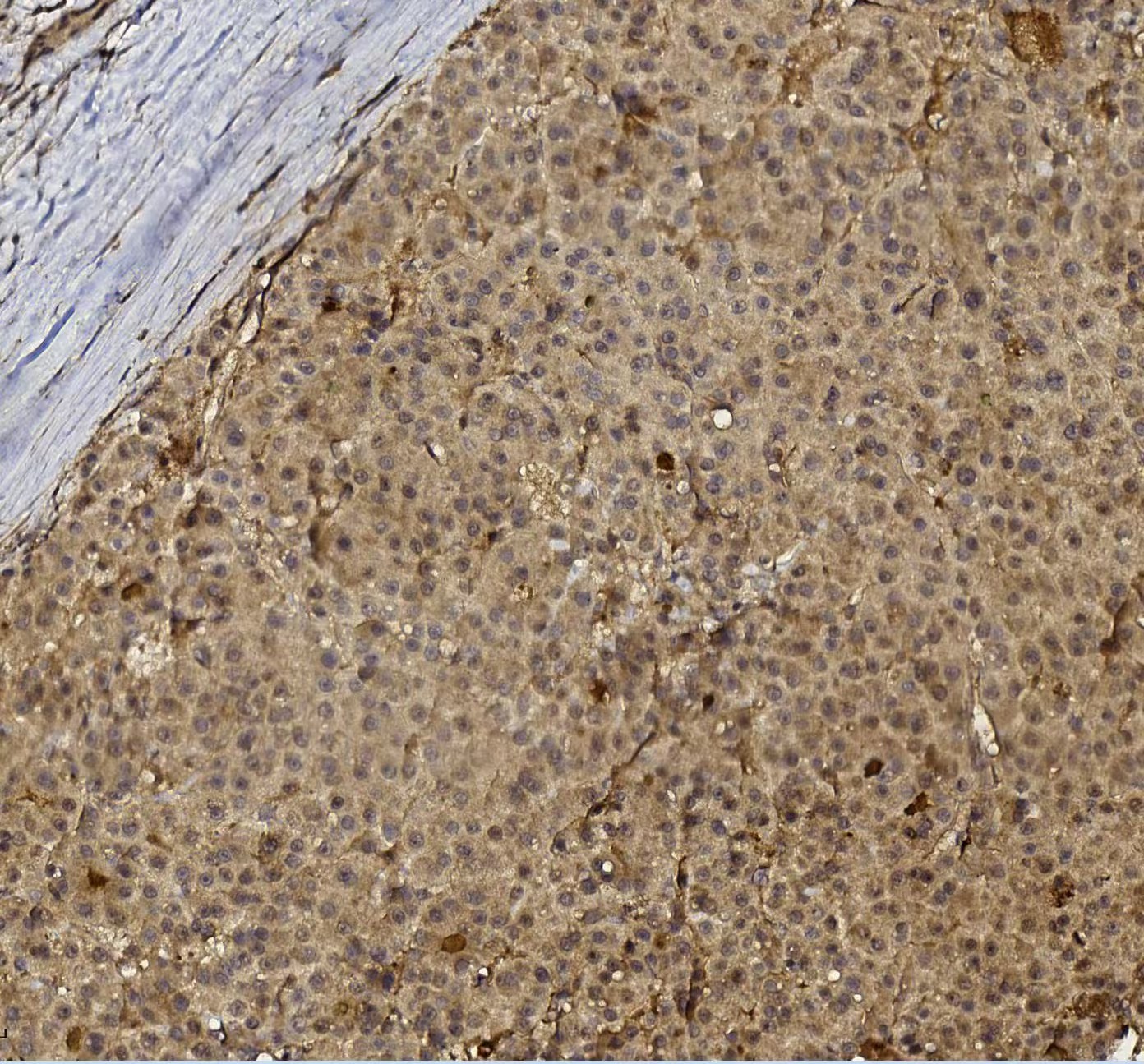

Supplement: Supplementary file 1 [file DataSheet1.zip › Raw data file/Cancerous tissues 2.jpg]

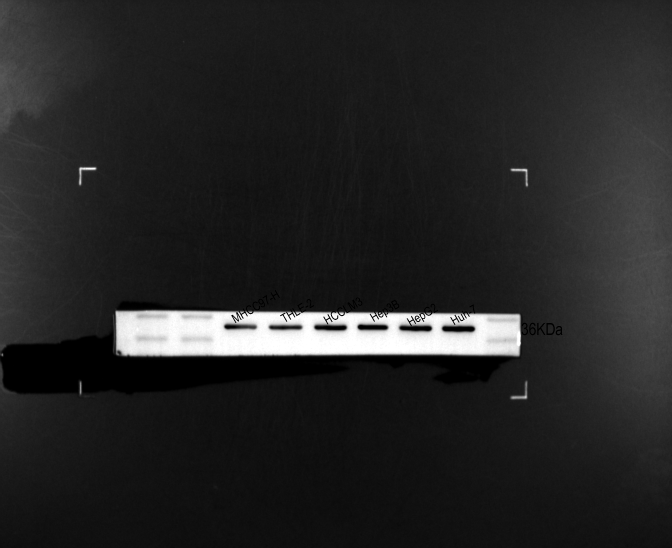

Supplement: Supplementary file 1 [file DataSheet1.zip › Raw data file/GADPH.tif]

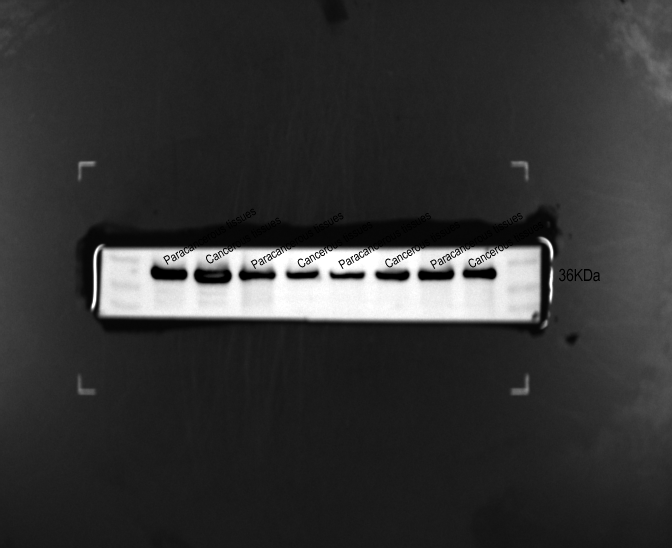

Supplement: Supplementary file 1 [file DataSheet1.zip › Raw data file/GADPH4 1.tif]

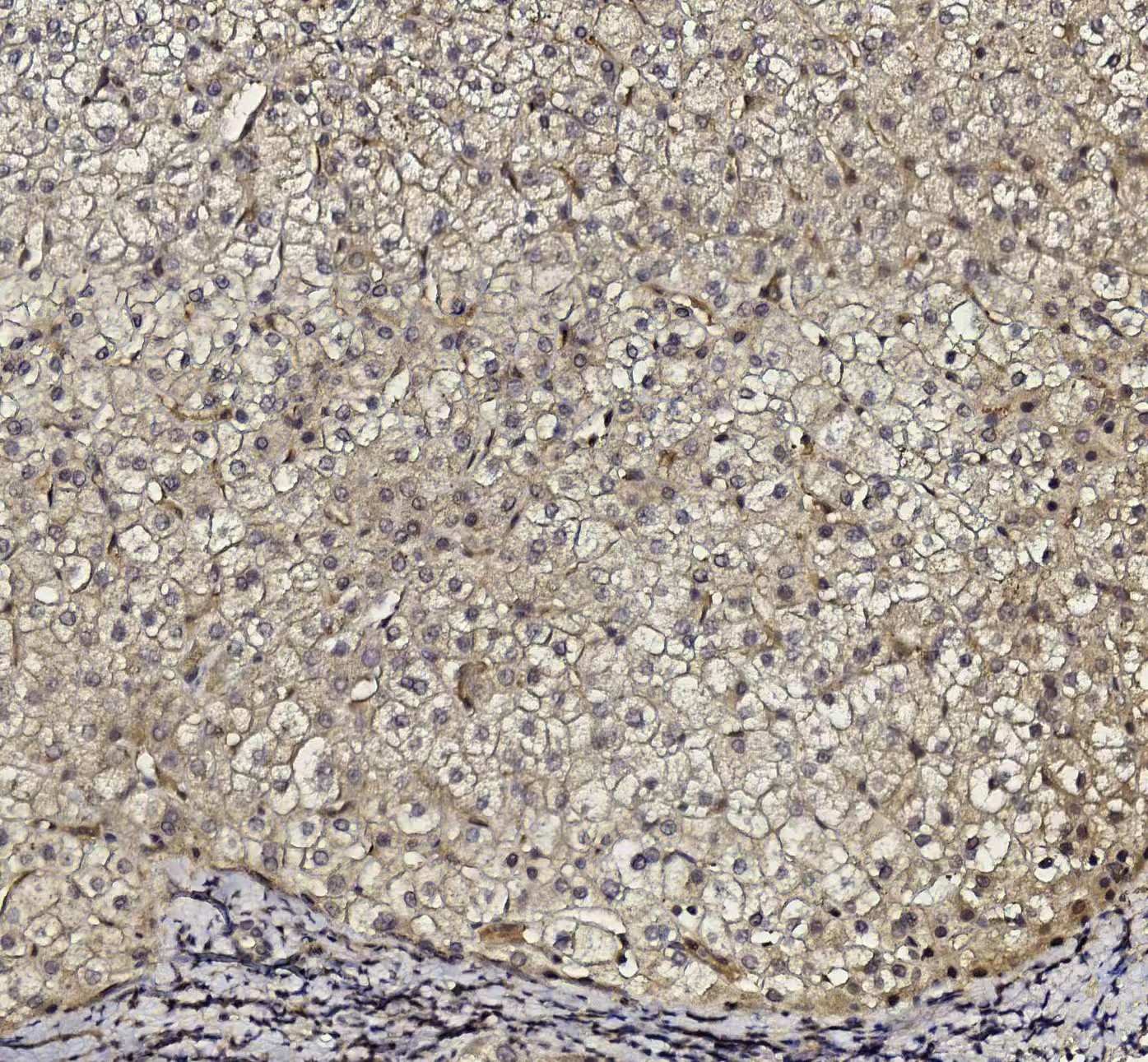

Supplement: Supplementary file 1 [file DataSheet1.zip › Raw data file/Paracancerous tissues 1.jpg]

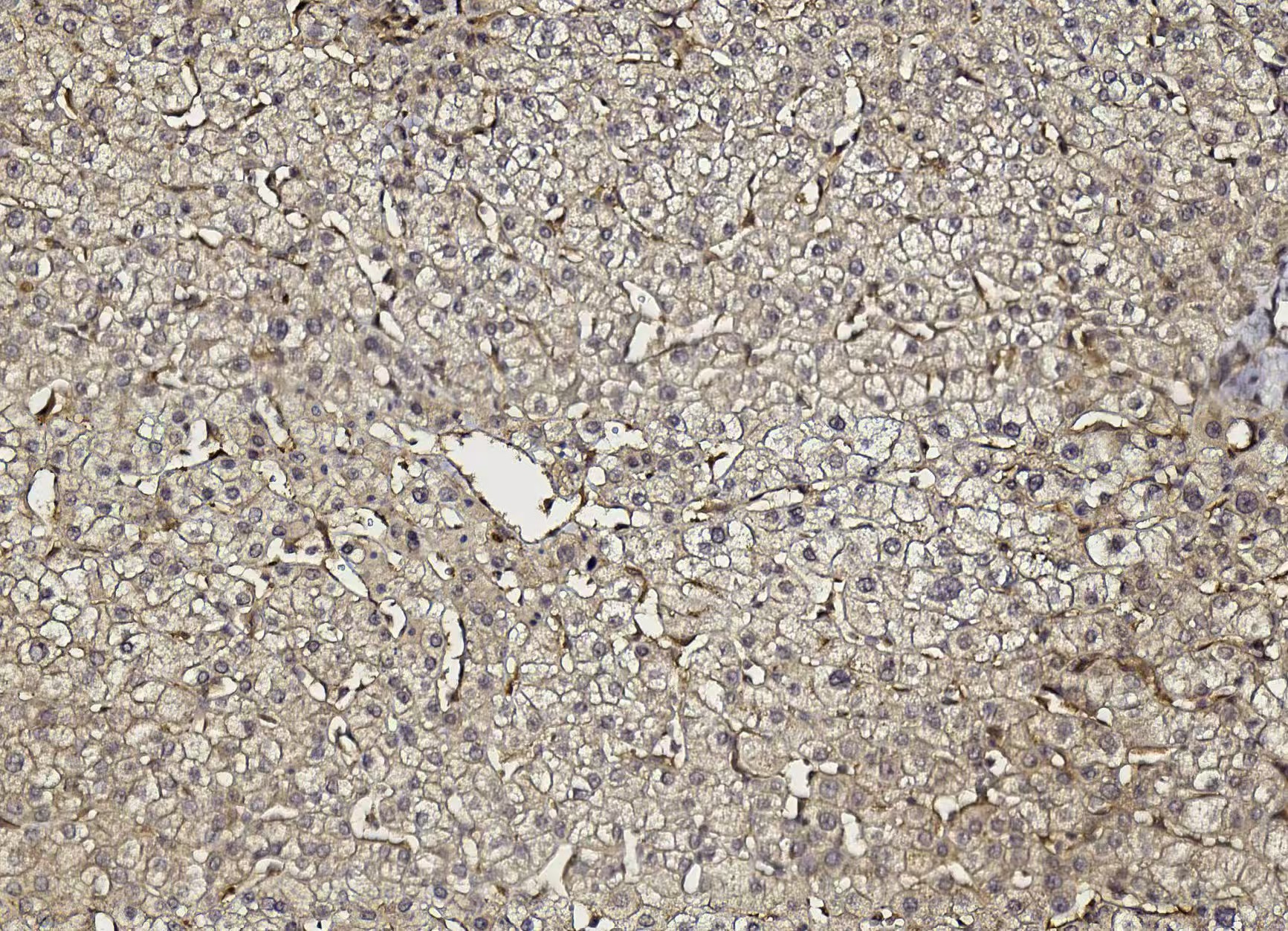

Supplement: Supplementary file 1 [file DataSheet1.zip › Raw data file/Paracancerous tissues 2.jpg]

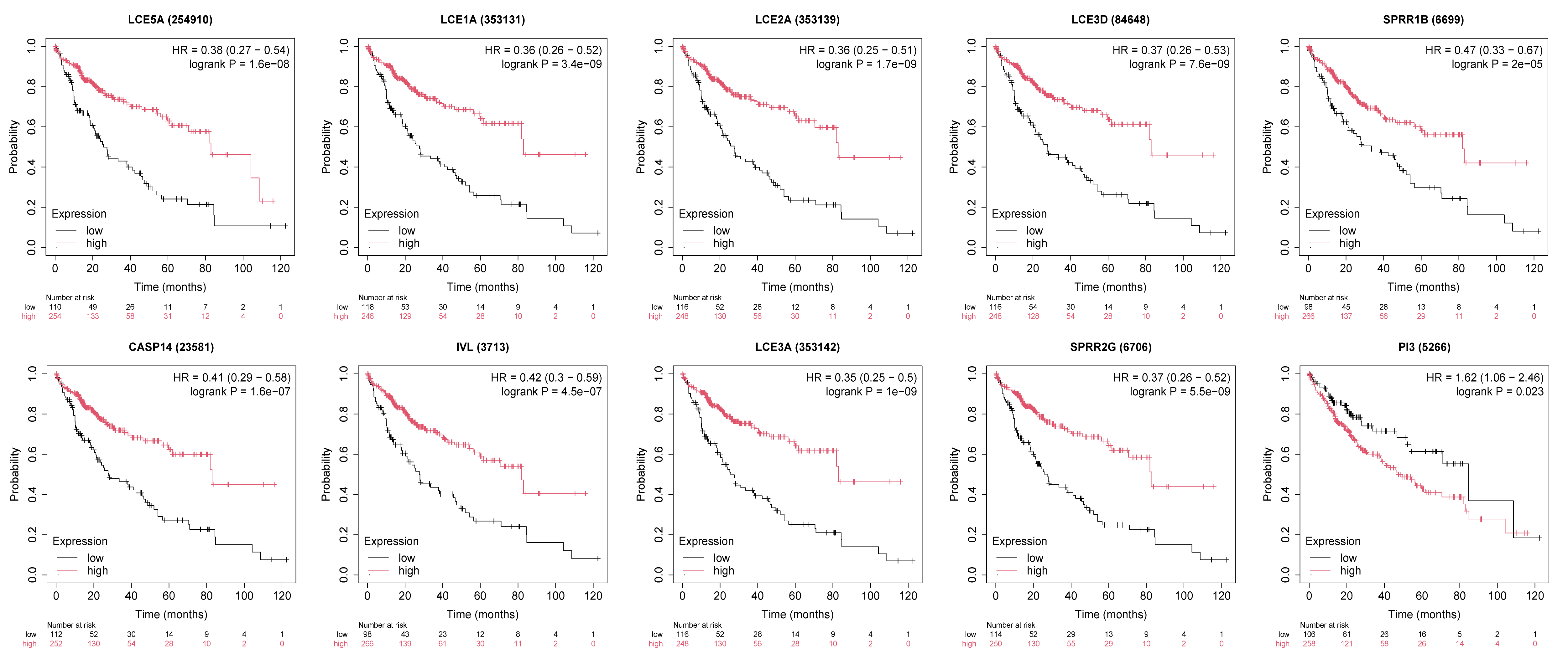

Supplement: Supplementary file 1 [file DataSheet1.zip › Raw data file/Supplementary Figure 1..tif]

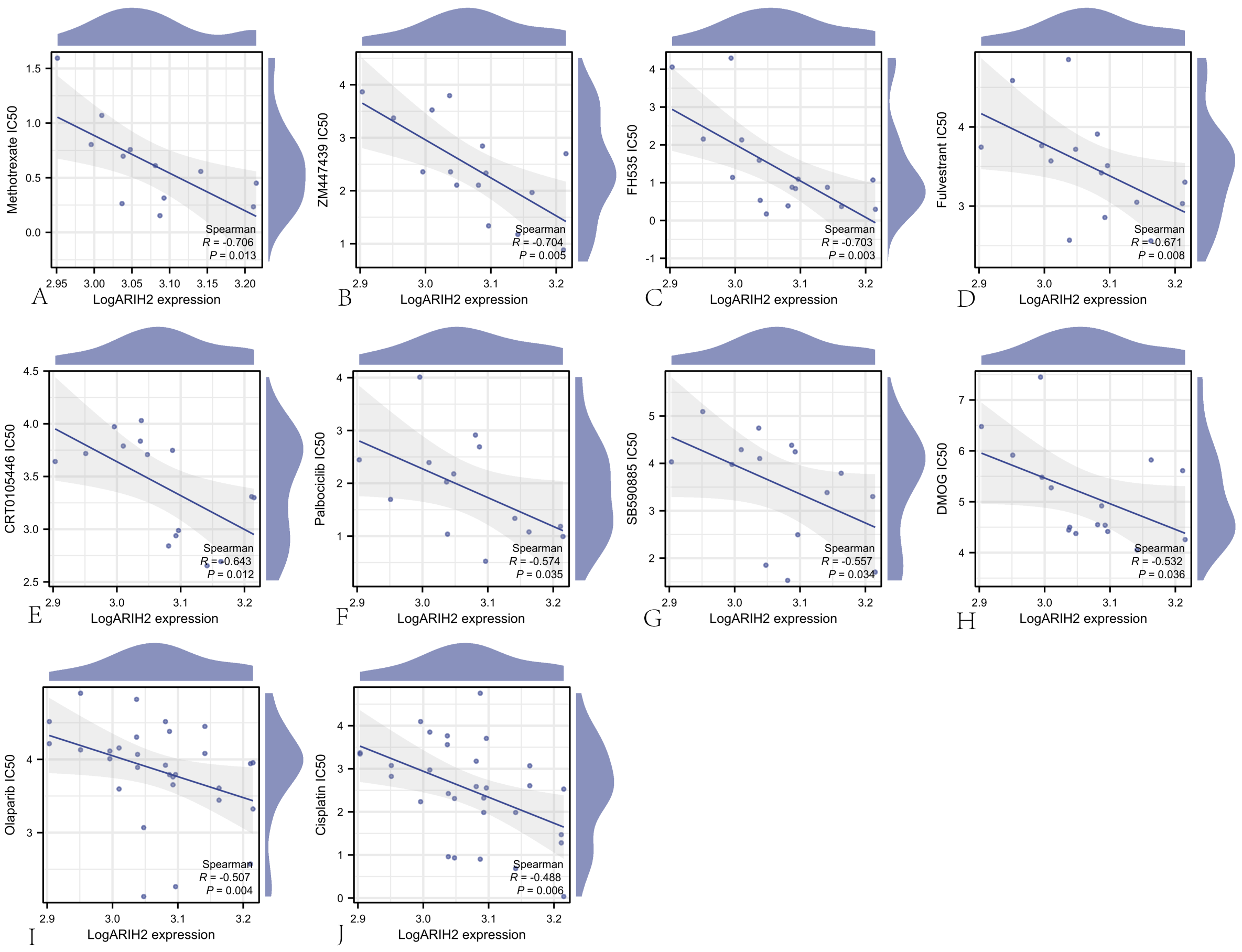

Supplement: Supplementary file 1 [file DataSheet1.zip › Raw data file/Supplementary Figure 2.tif]

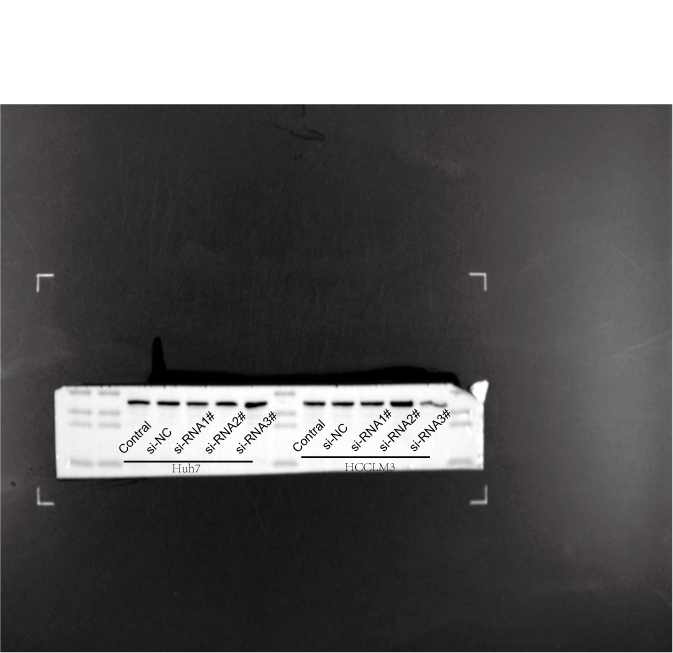

Supplement: Supplementary file 2 [file DataSheet2.zip › WB/ARIH2 ,.tif]

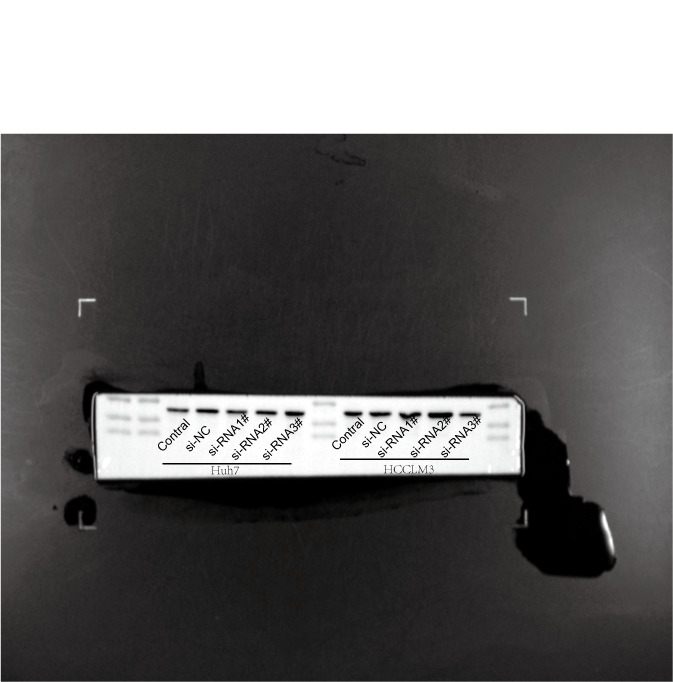

Supplement: Supplementary file 2 [file DataSheet2.zip › WB/ARIH2-1,.tif]

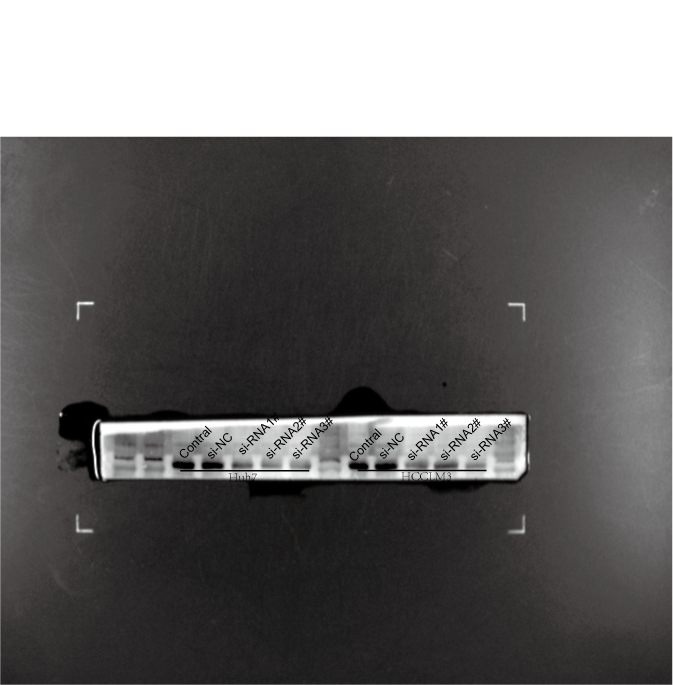

Supplement: Supplementary file 2 [file DataSheet2.zip › WB/ARIH2-1.tif]

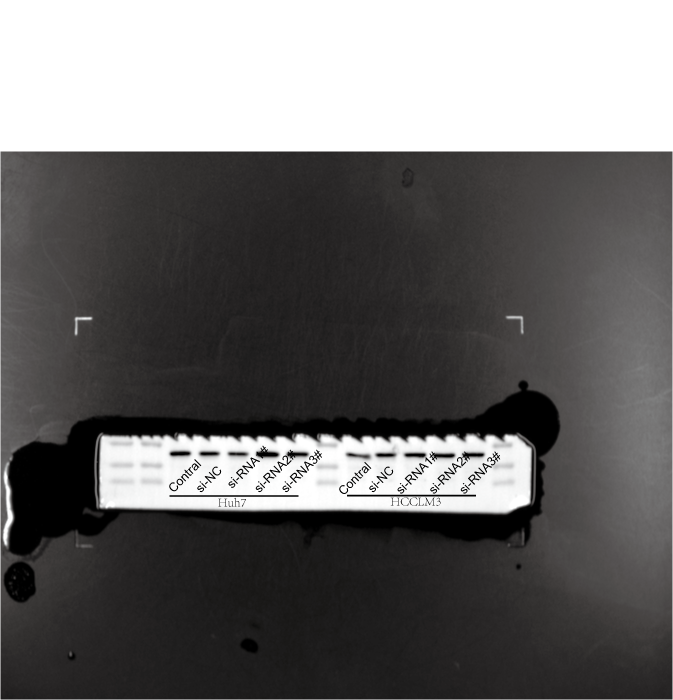

Supplement: Supplementary file 2 [file DataSheet2.zip › WB/ARIH2-2,.tif]

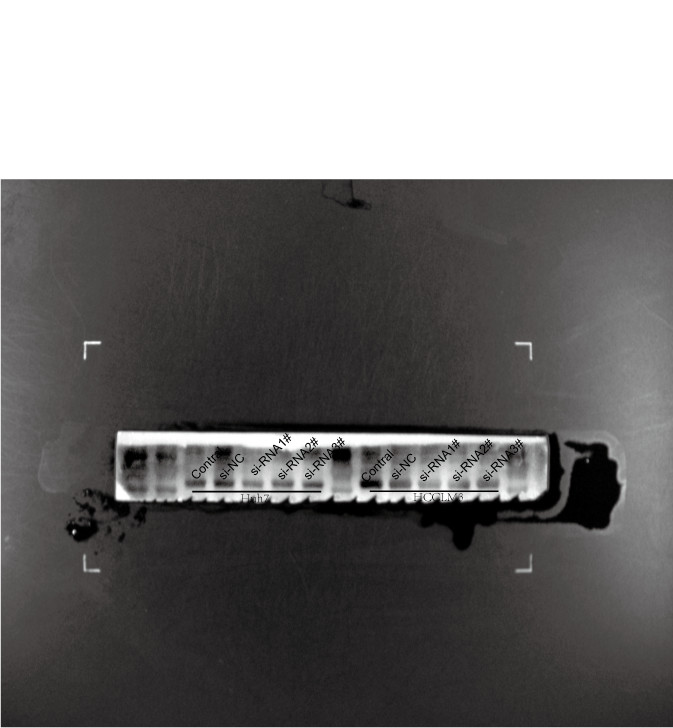

Supplement: Supplementary file 2 [file DataSheet2.zip › WB/ARIH2-2.tif]

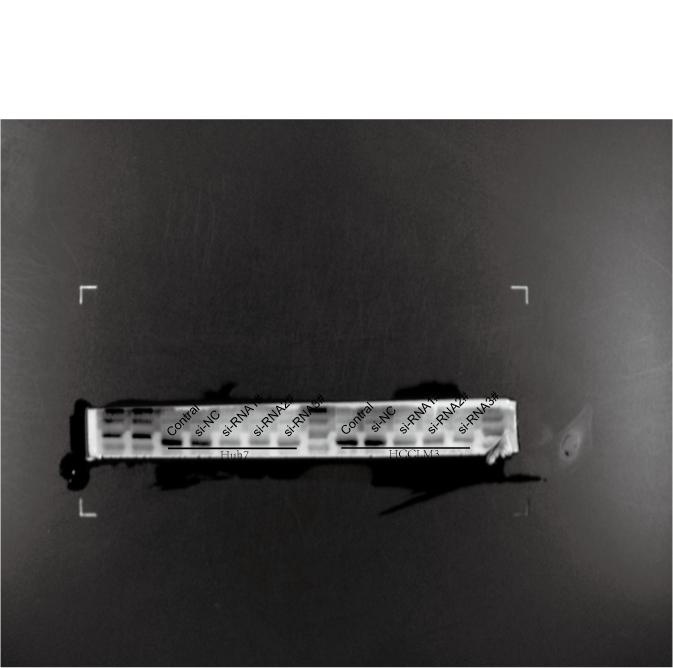

Supplement: Supplementary file 2 [file DataSheet2.zip › WB/ARIH2.tif]

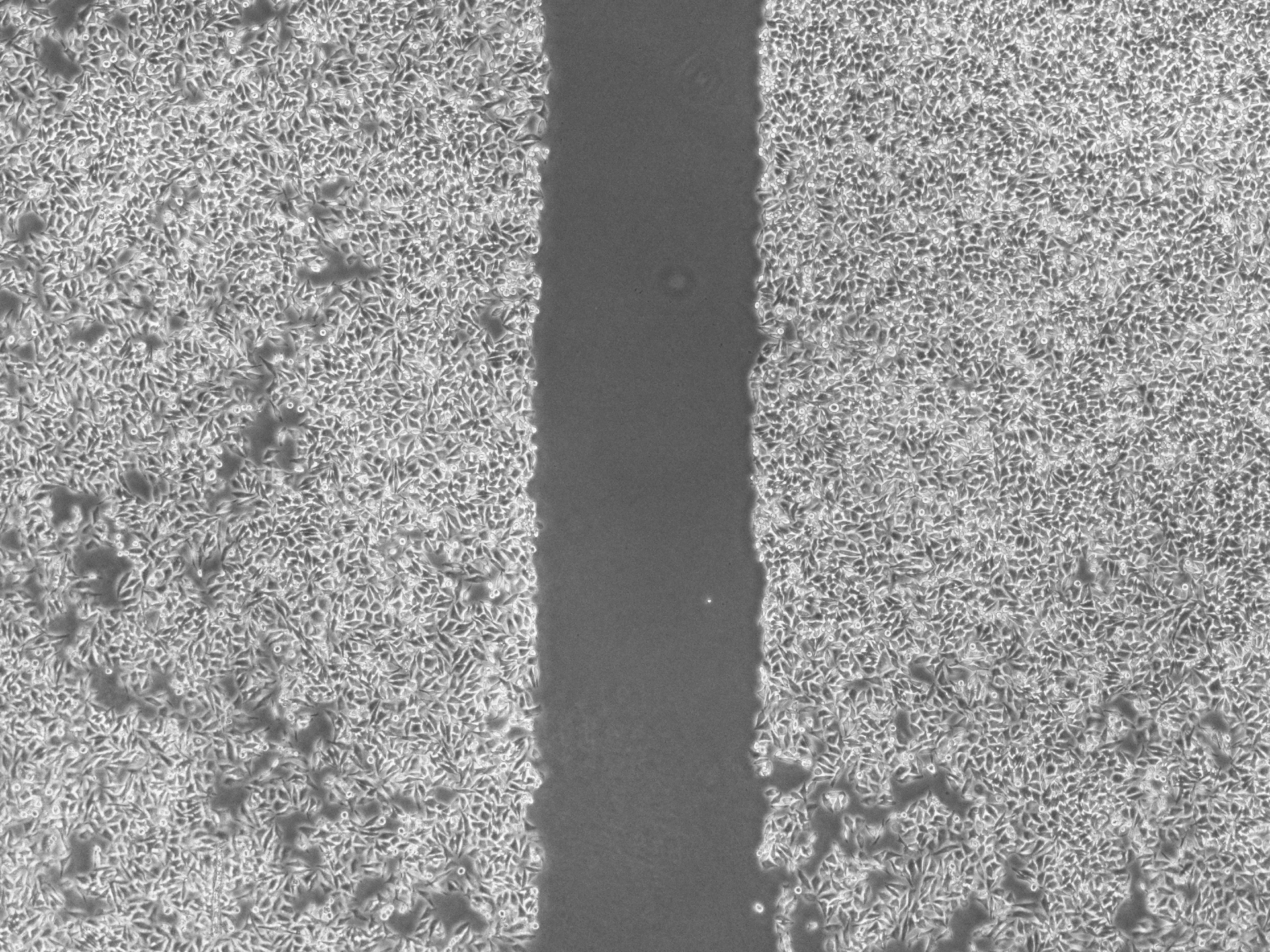

Supplement: Supplementary file 3 [file DataSheet3.zip › HCCLM3 wound healing assay/LM3nc1 0h.tif]

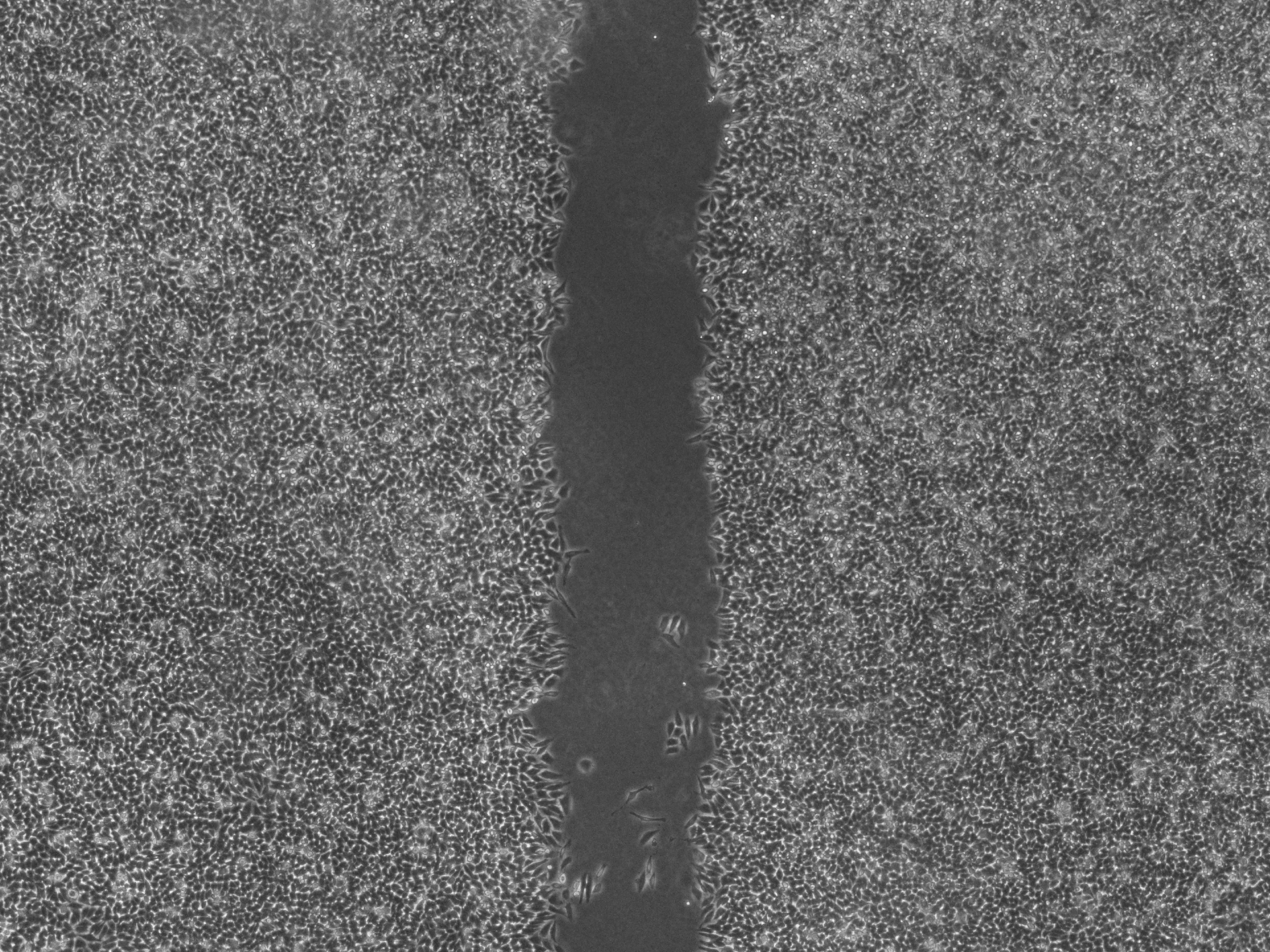

Supplement: Supplementary file 3 [file DataSheet3.zip › HCCLM3 wound healing assay/LM3nc1 48h.tif]

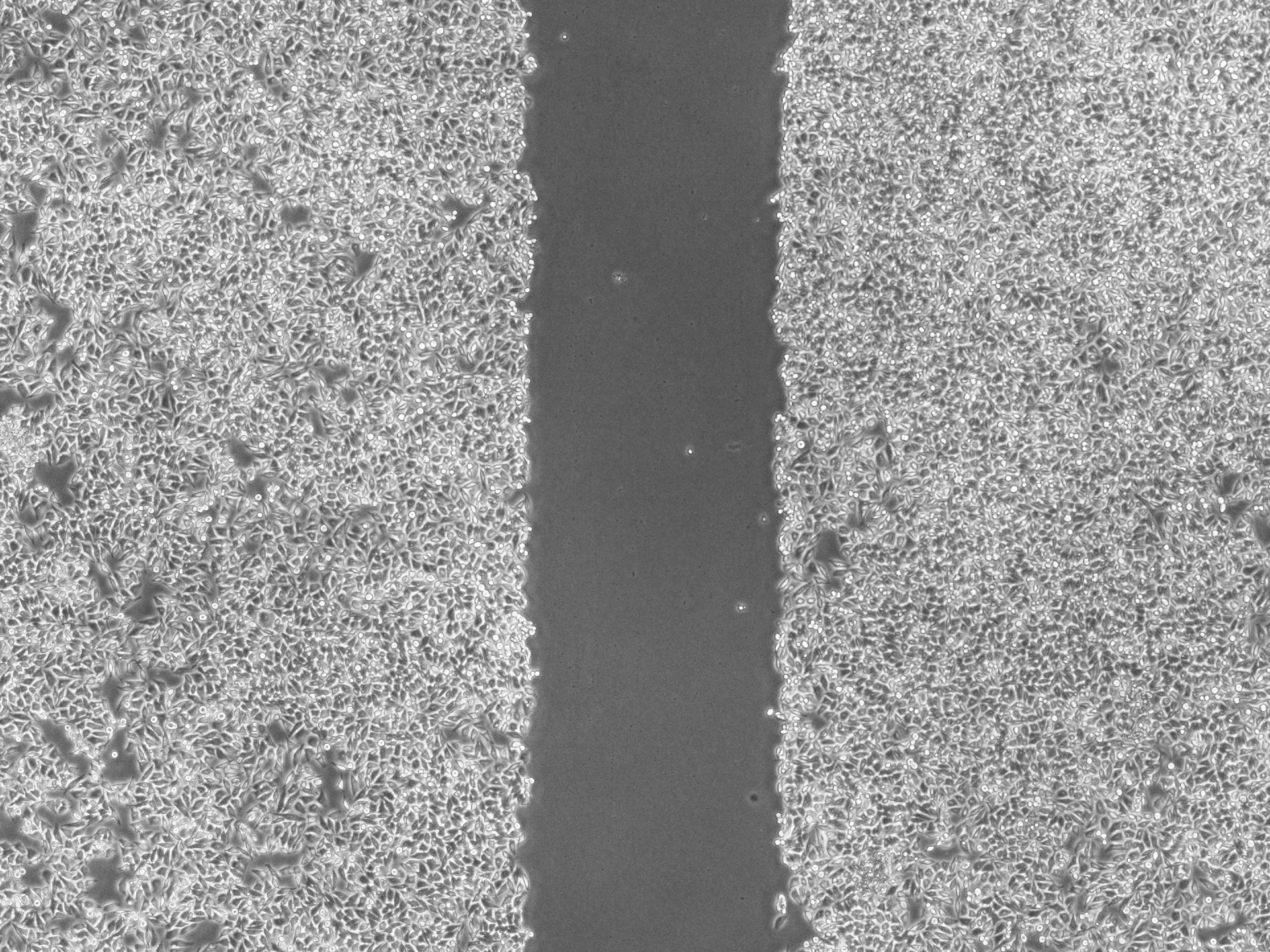

Supplement: Supplementary file 3 [file DataSheet3.zip › HCCLM3 wound healing assay/LM3nc2 0h.tif]

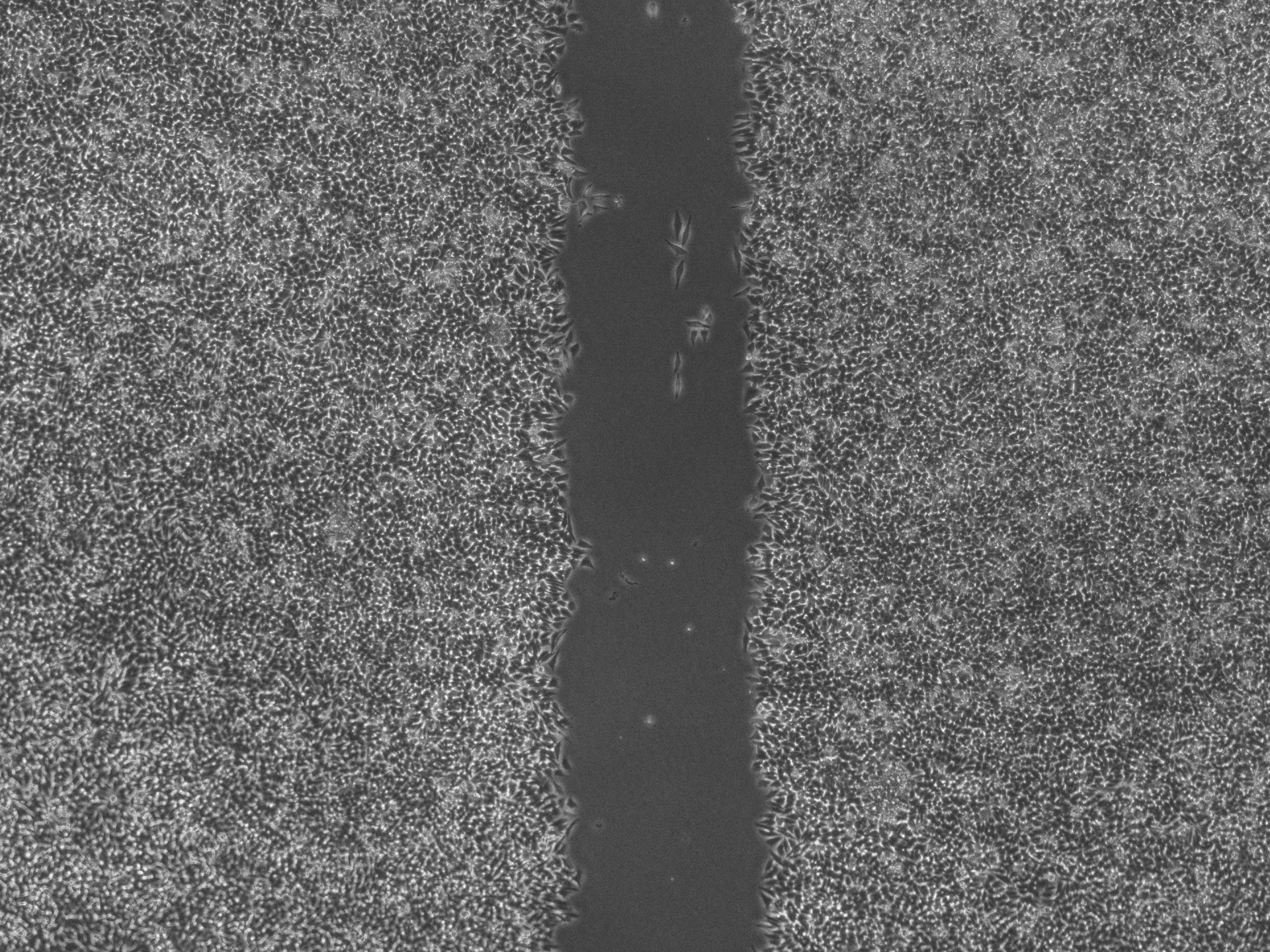

Supplement: Supplementary file 3 [file DataSheet3.zip › HCCLM3 wound healing assay/LM3nc2 48h.tif]

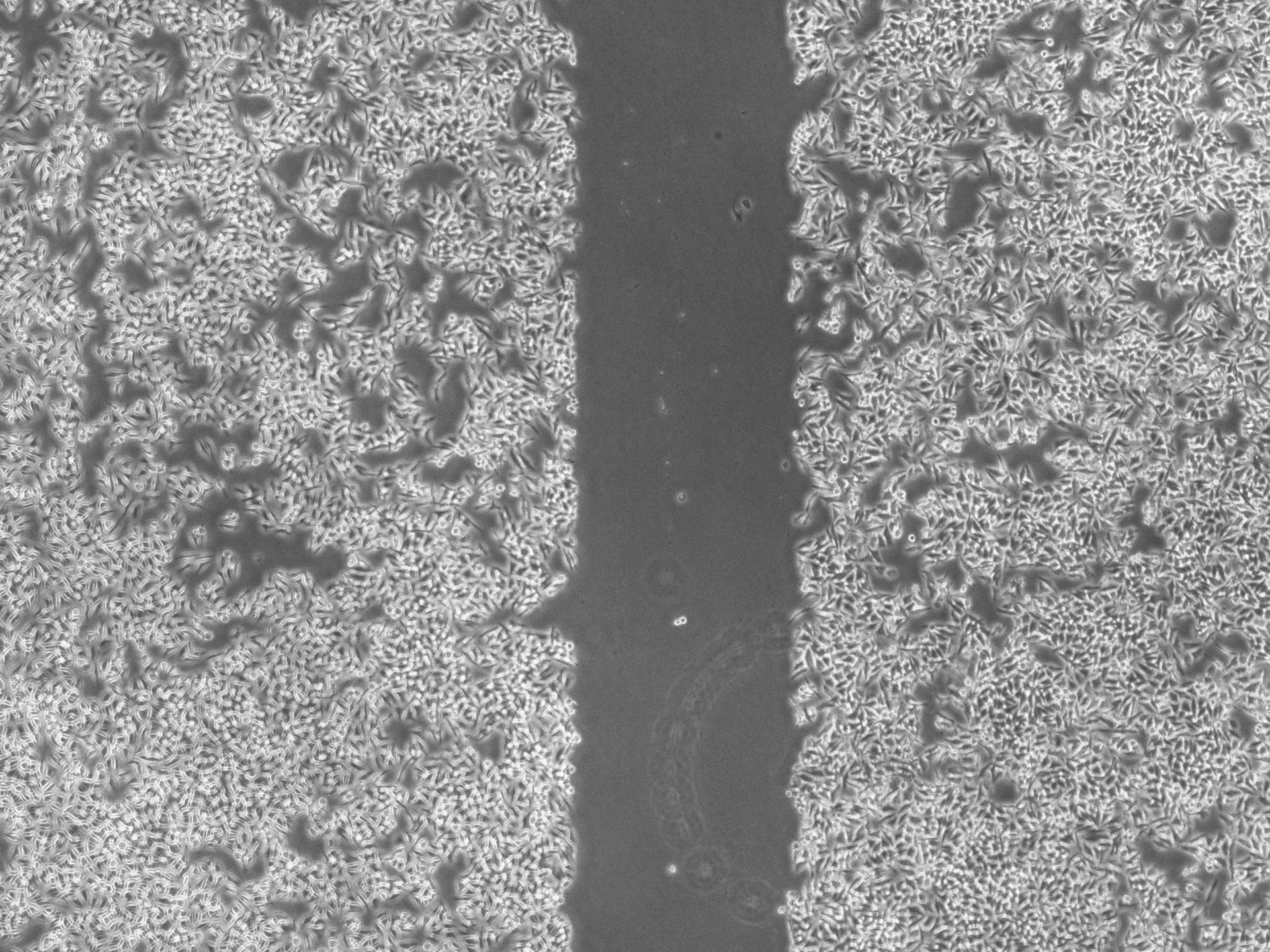

Supplement: Supplementary file 3 [file DataSheet3.zip › HCCLM3 wound healing assay/LM3nc3 0h.tif]

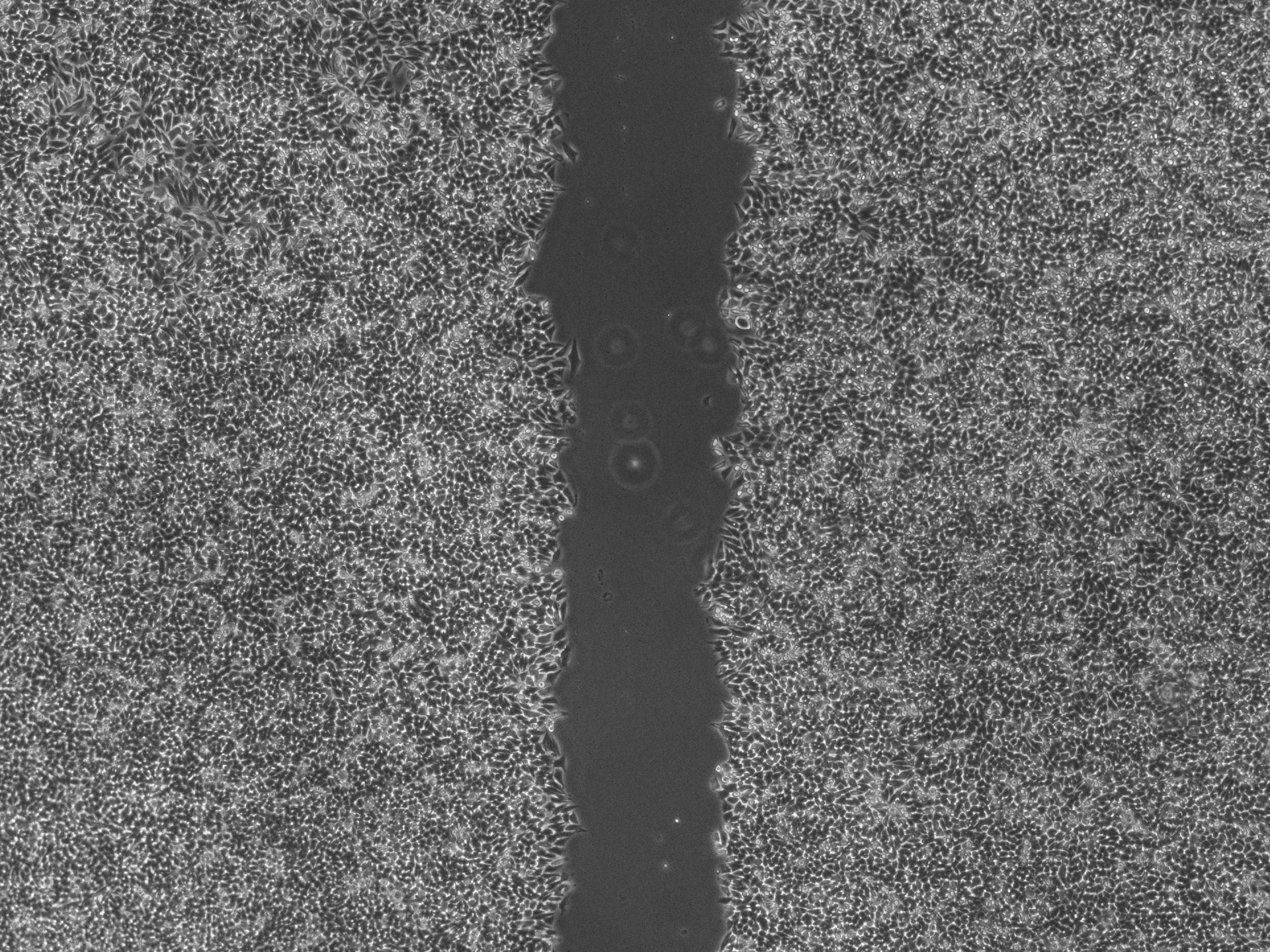

Supplement: Supplementary file 3 [file DataSheet3.zip › HCCLM3 wound healing assay/LM3nc3 48h.tif]

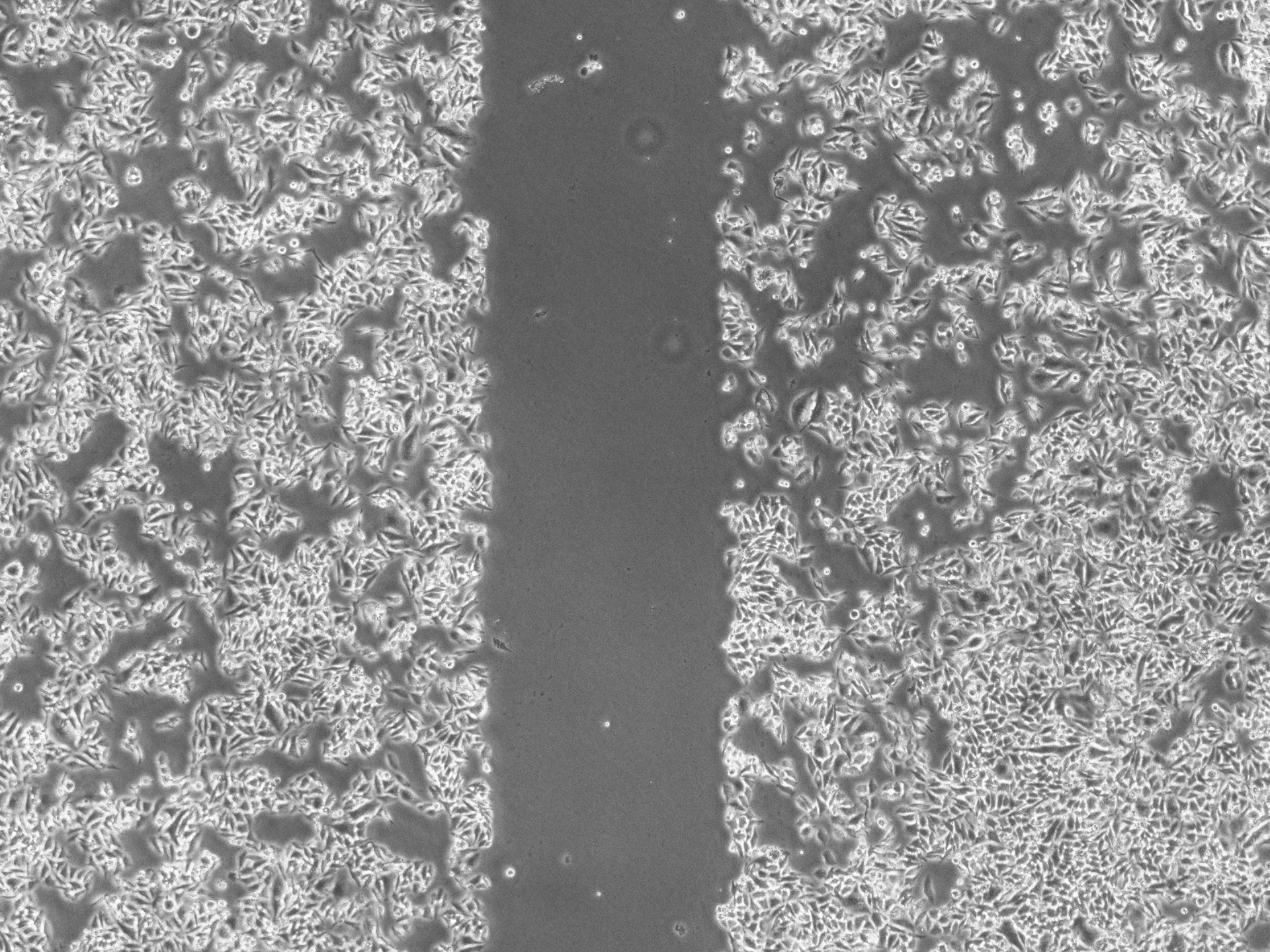

Supplement: Supplementary file 3 [file DataSheet3.zip › HCCLM3 wound healing assay/LM3si1 0h.tif]

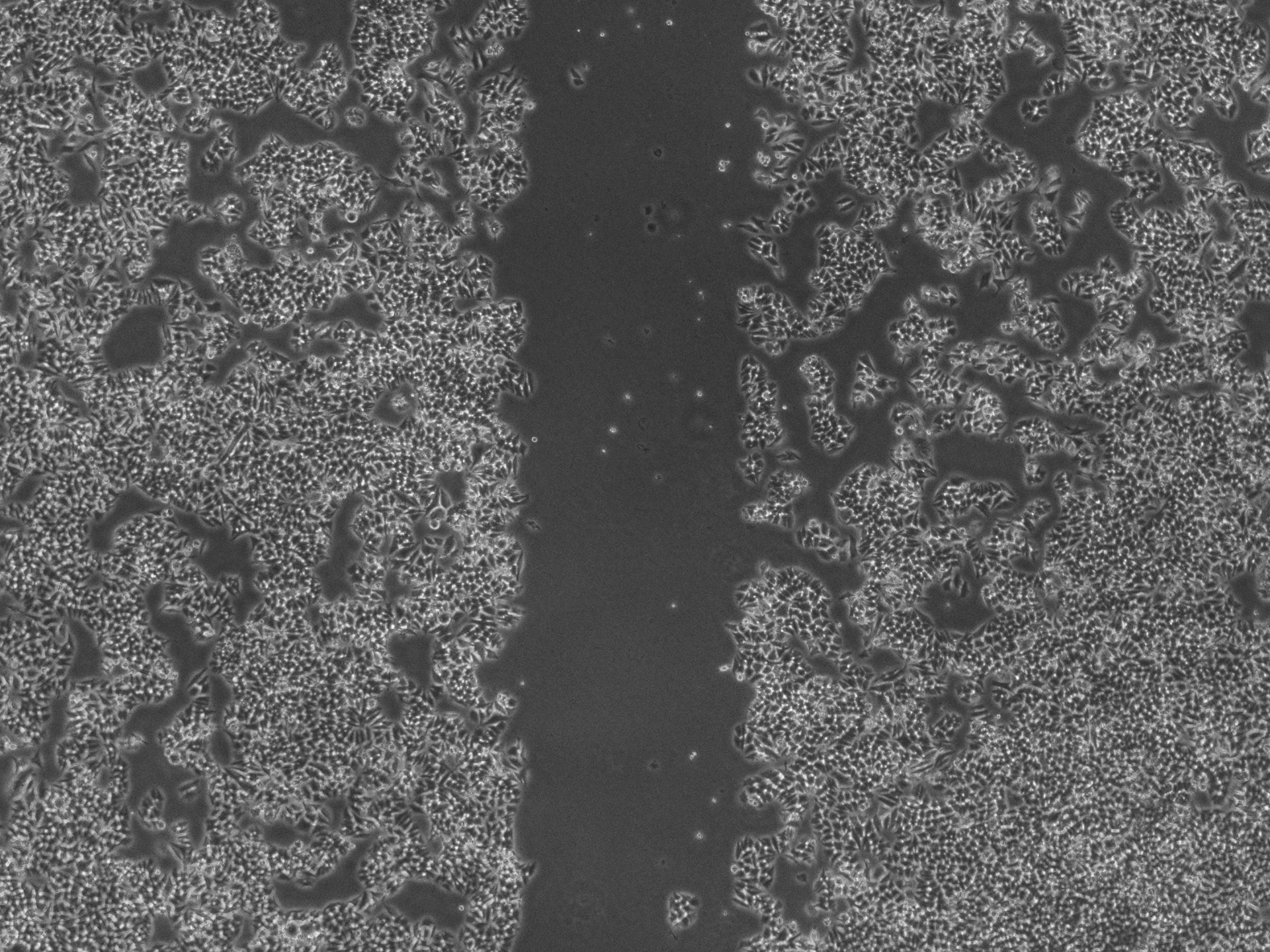

Supplement: Supplementary file 3 [file DataSheet3.zip › HCCLM3 wound healing assay/LM3si1 48h.tif]

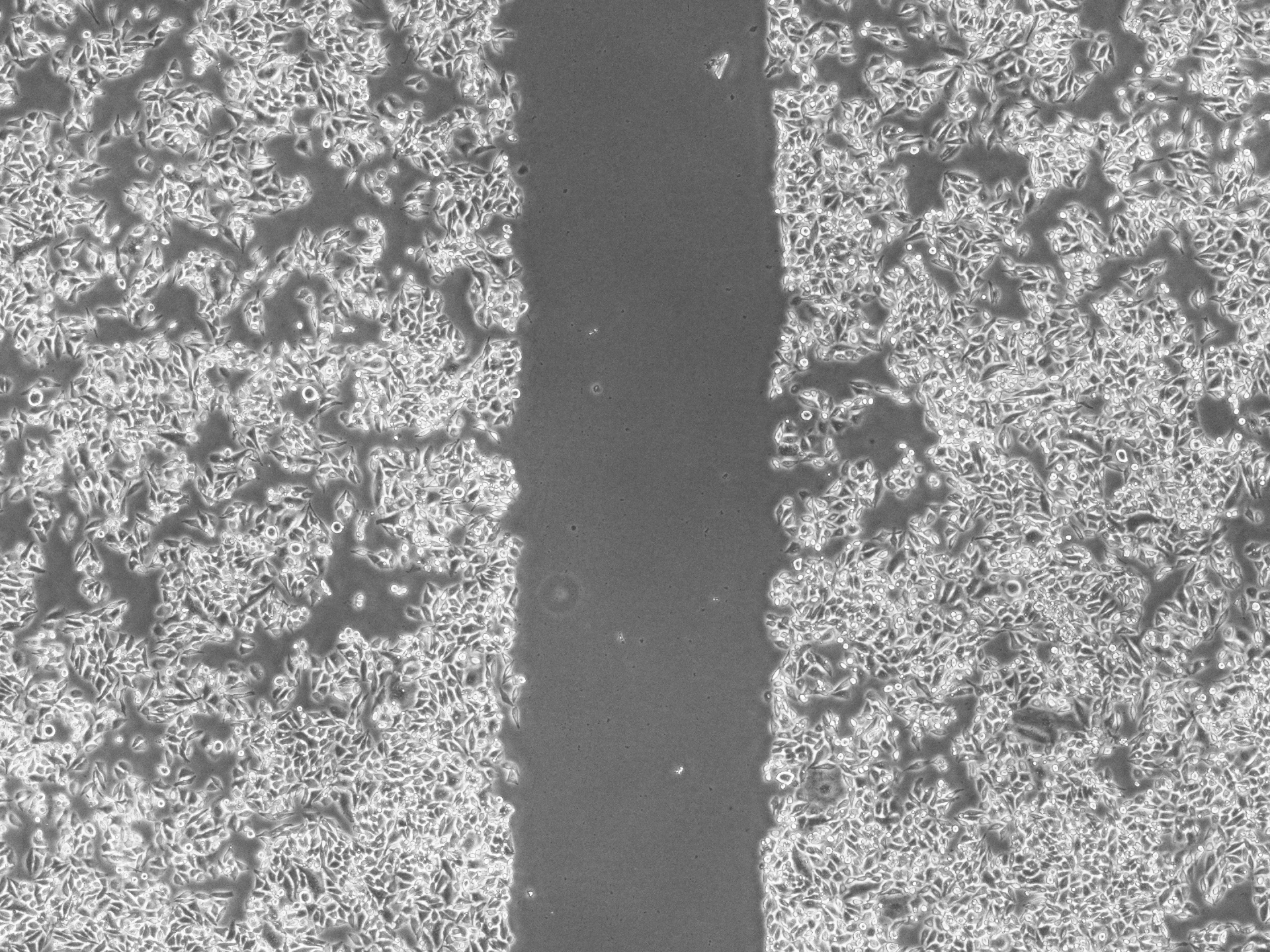

Supplement: Supplementary file 3 [file DataSheet3.zip › HCCLM3 wound healing assay/LM3si1-1 0h.tif]

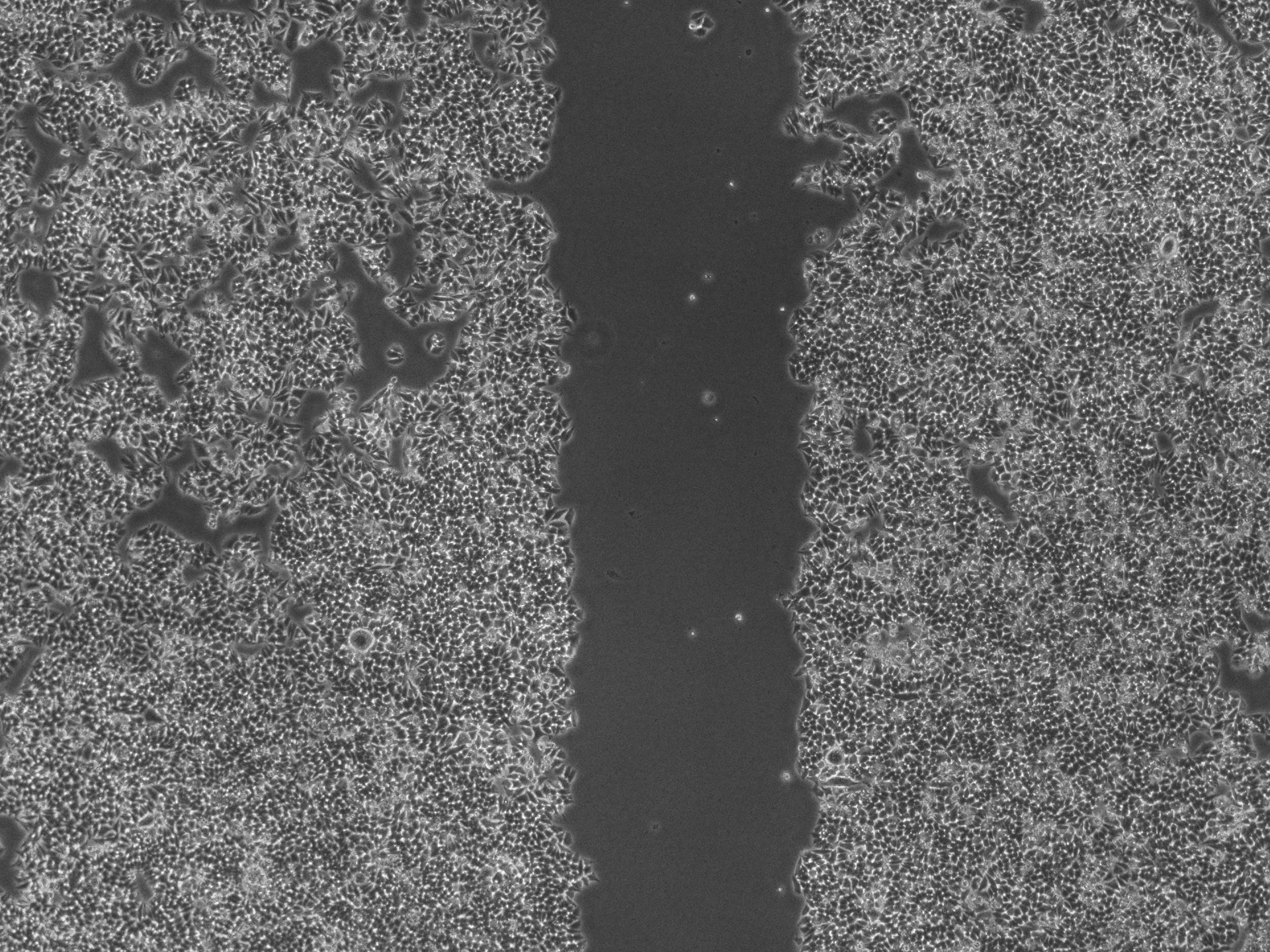

Supplement: Supplementary file 3 [file DataSheet3.zip › HCCLM3 wound healing assay/LM3si1-1 48h.tif]

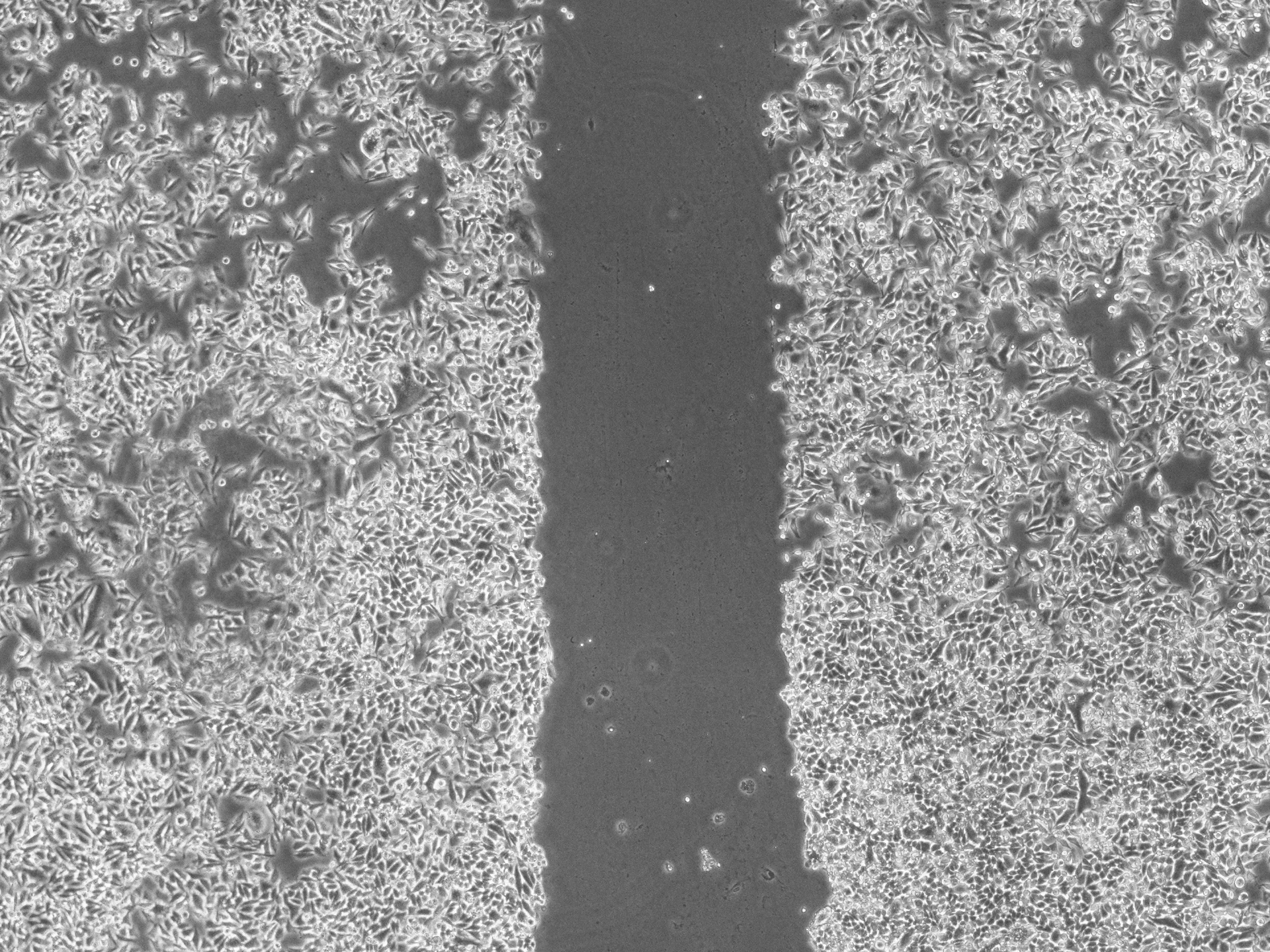

Supplement: Supplementary file 3 [file DataSheet3.zip › HCCLM3 wound healing assay/LM3si1-2 0h.tif]

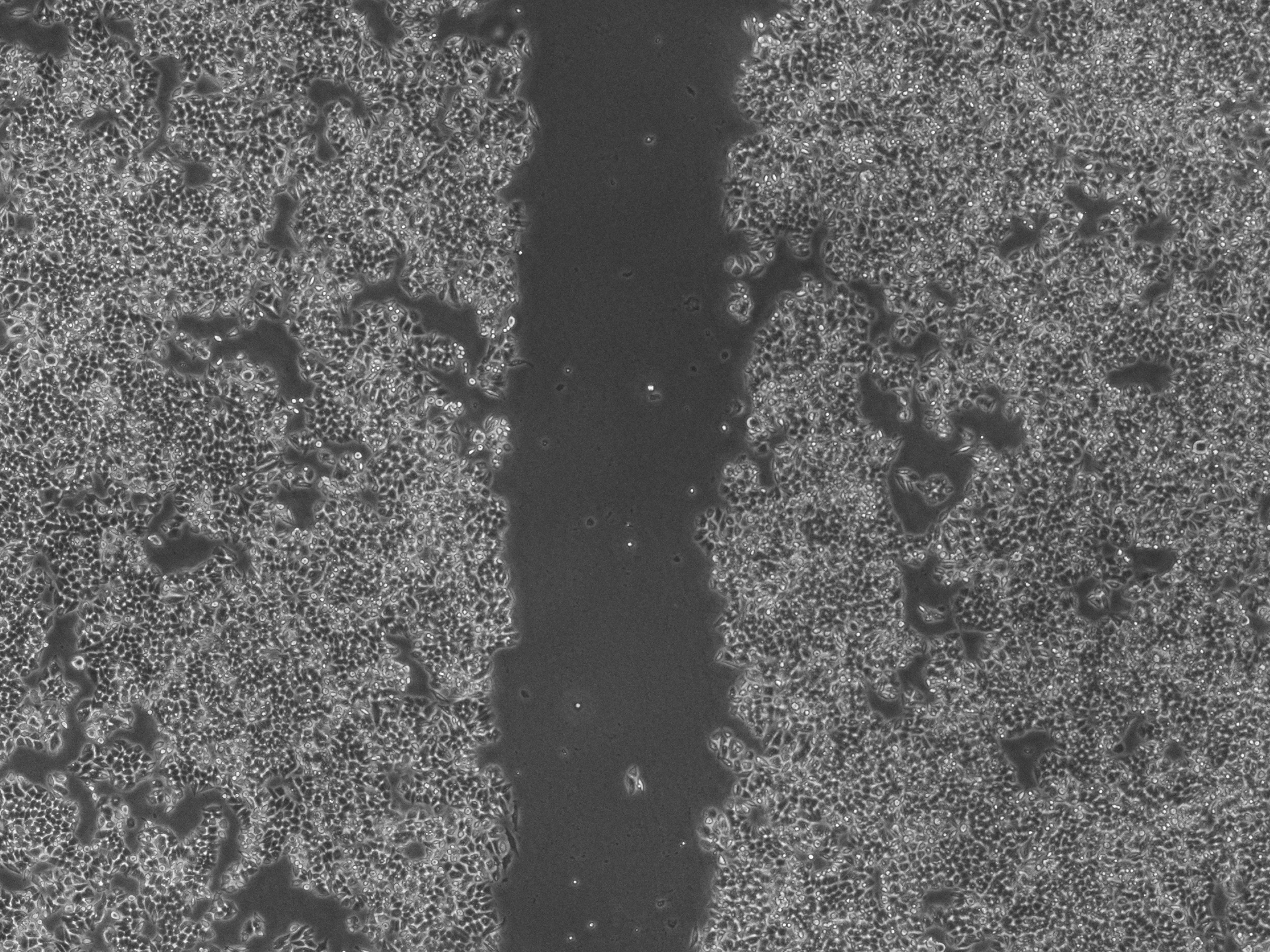

Supplement: Supplementary file 3 [file DataSheet3.zip › HCCLM3 wound healing assay/LM3si1-2 48h.tif]

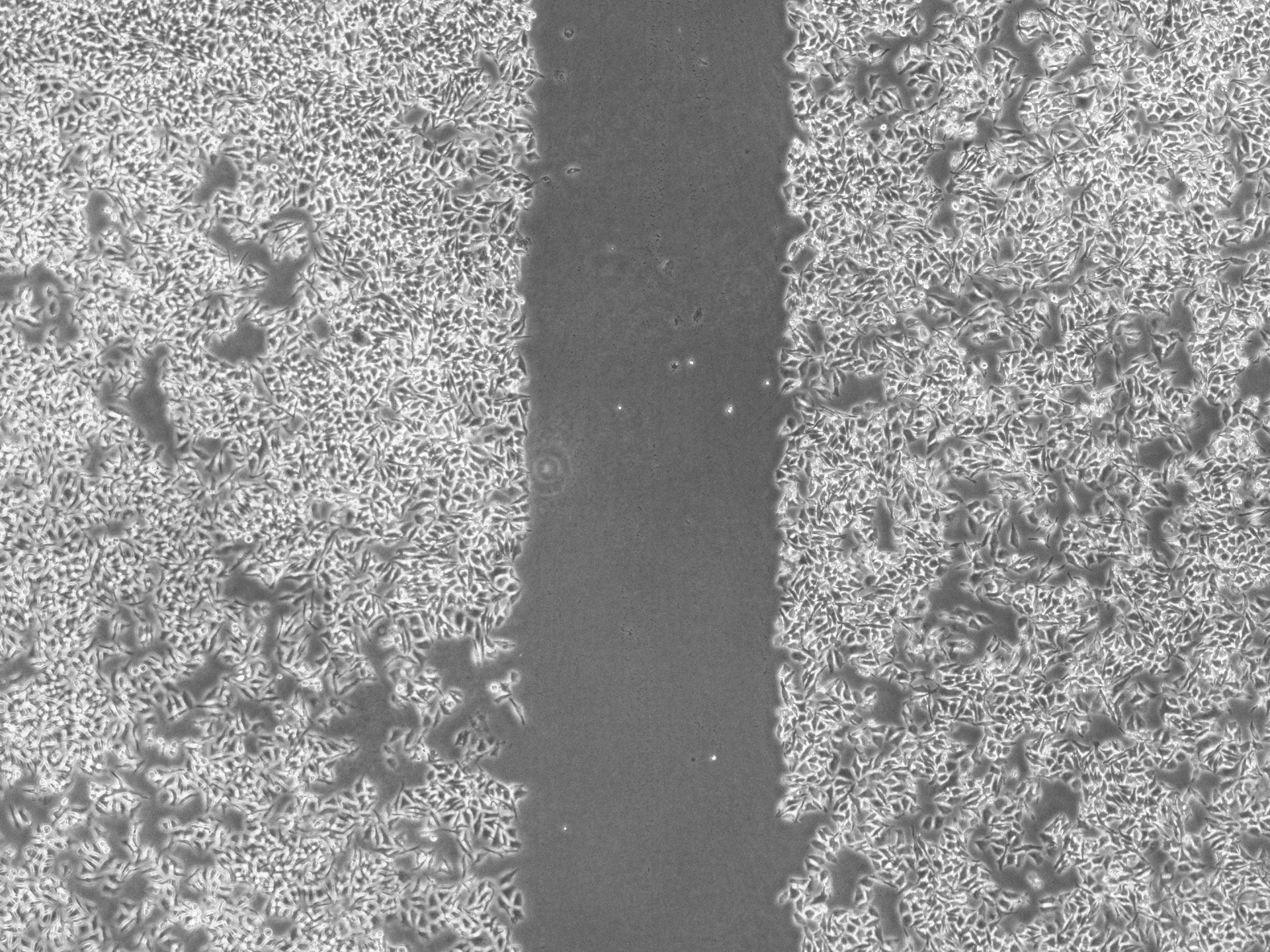

Supplement: Supplementary file 4 [file DataSheet4.zip › Huh7 wound healing assay/H7nc1 0h.tif]

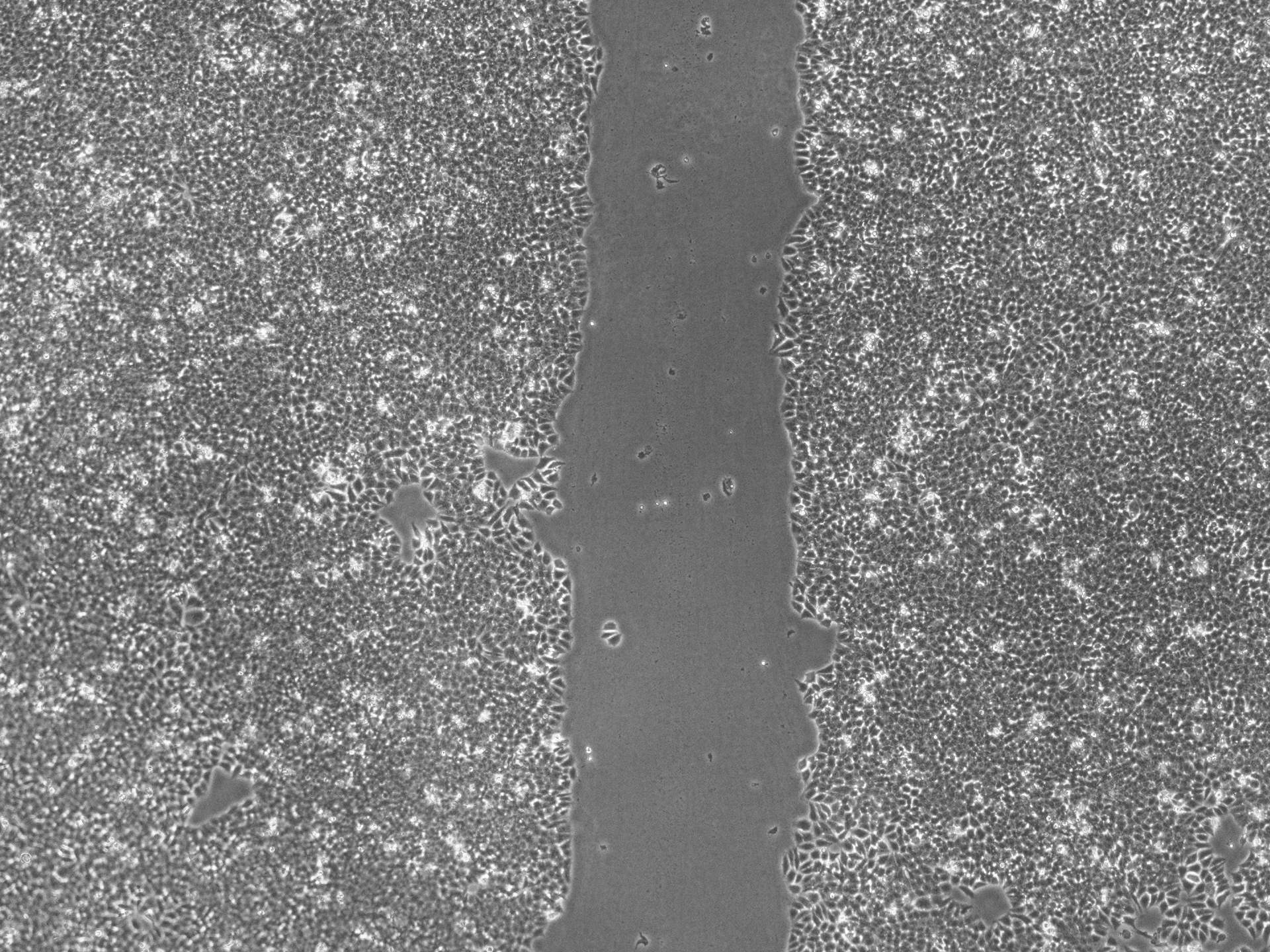

Supplement: Supplementary file 4 [file DataSheet4.zip › Huh7 wound healing assay/H7nc1 48h.tif]

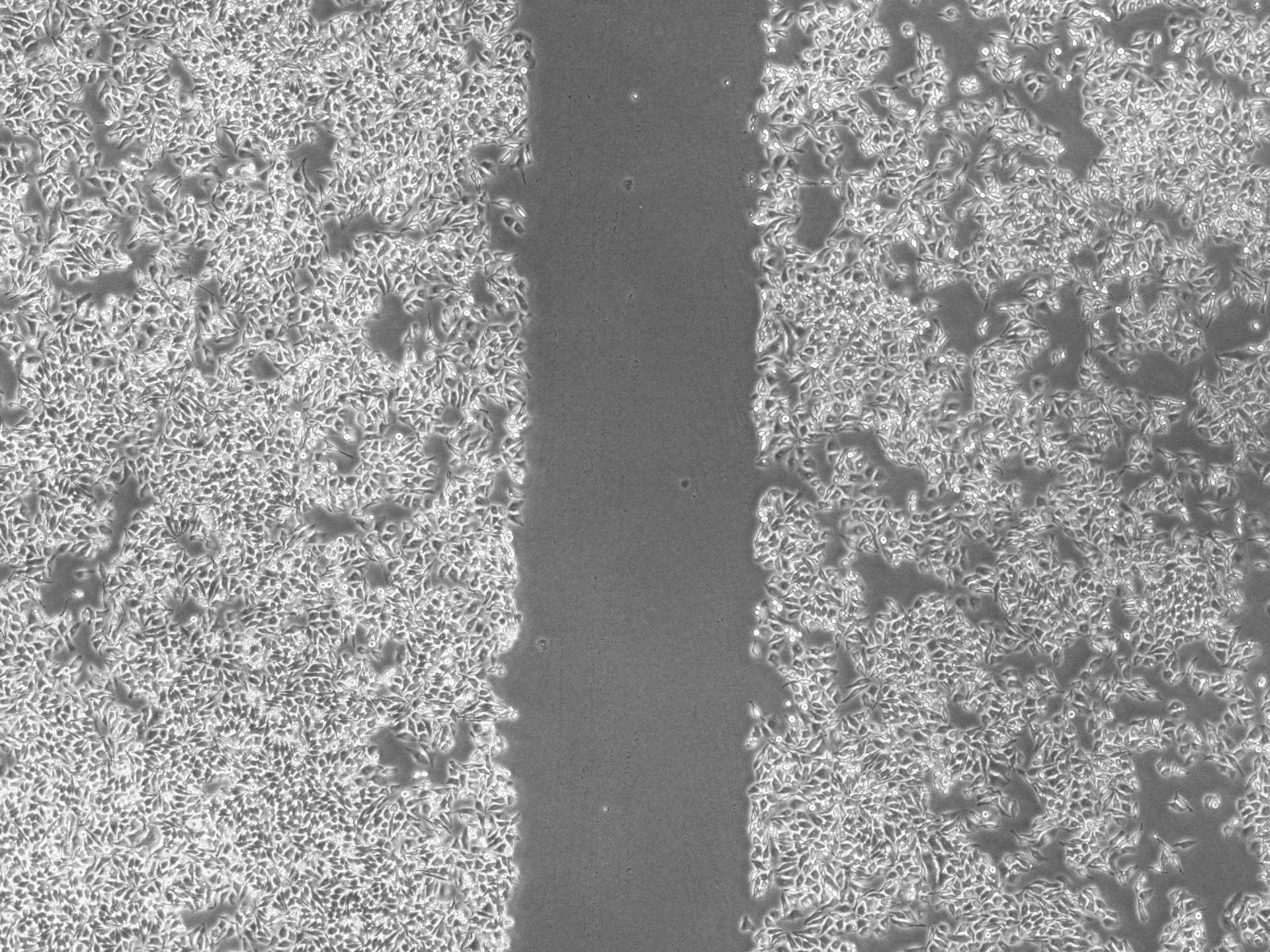

Supplement: Supplementary file 4 [file DataSheet4.zip › Huh7 wound healing assay/H7nc2 0h.tif]

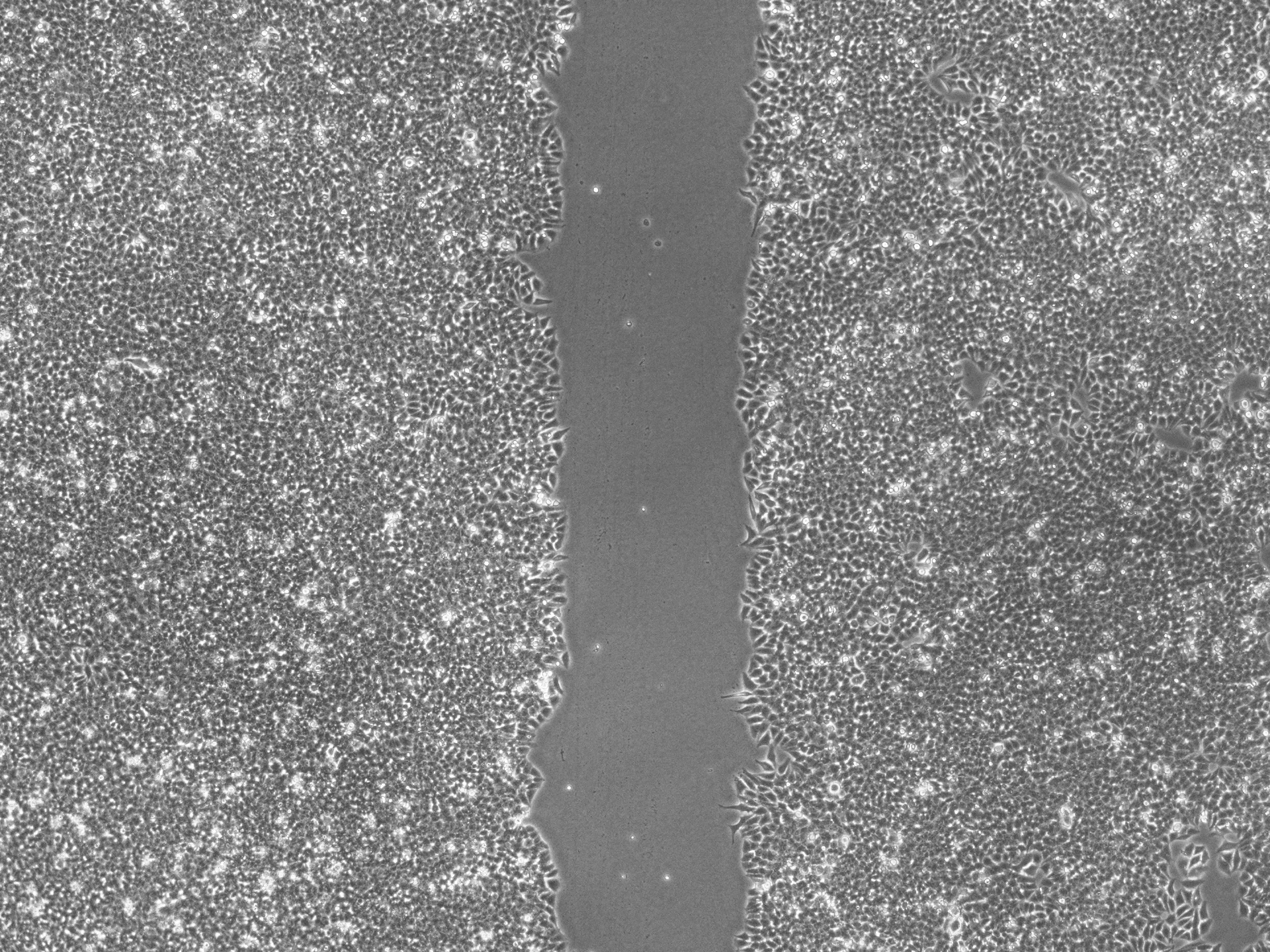

Supplement: Supplementary file 4 [file DataSheet4.zip › Huh7 wound healing assay/H7nc2 48h.tif]

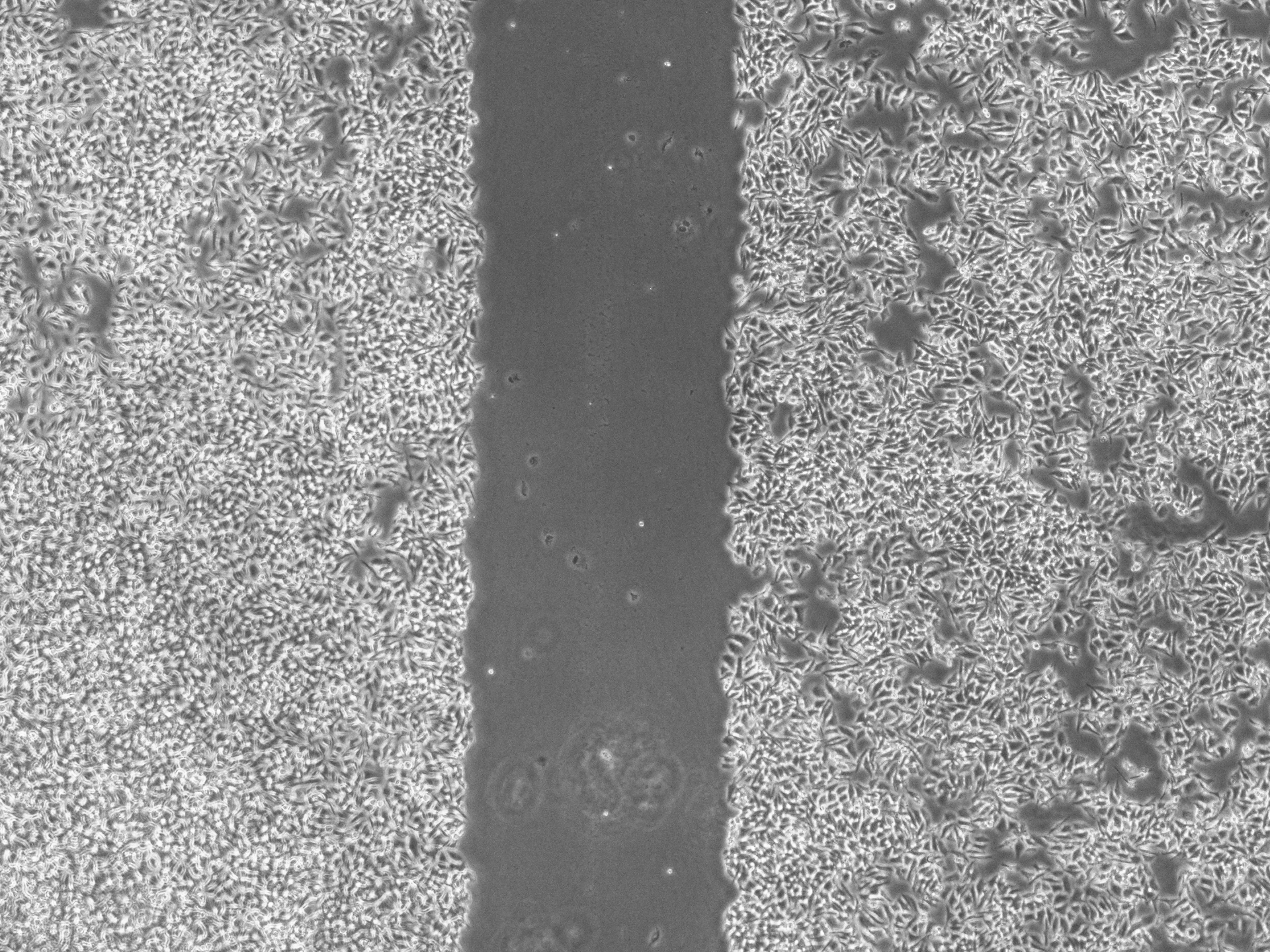

Supplement: Supplementary file 4 [file DataSheet4.zip › Huh7 wound healing assay/H7nc3 0h.tif]

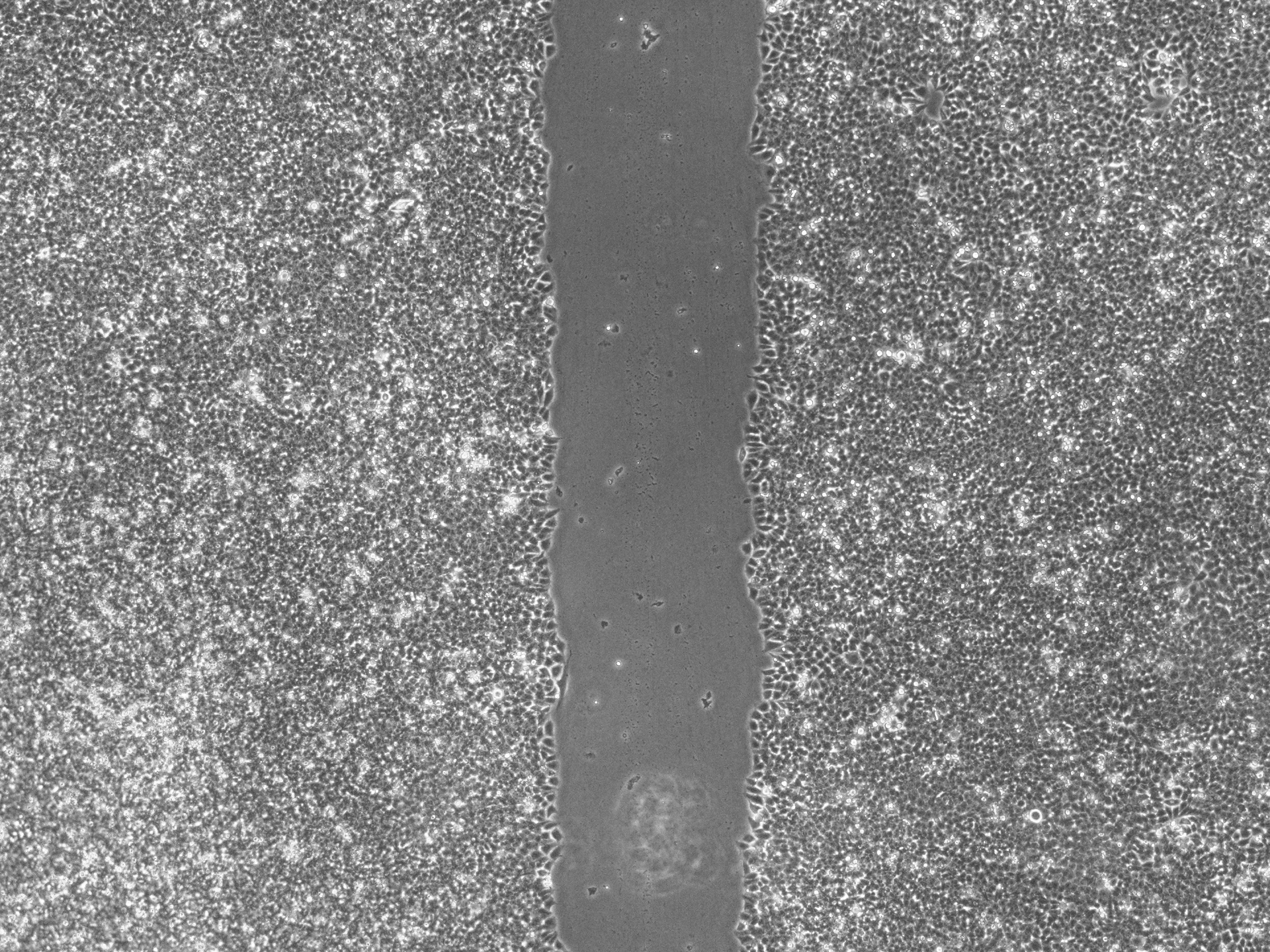

Supplement: Supplementary file 4 [file DataSheet4.zip › Huh7 wound healing assay/H7nc3 48h.tif]

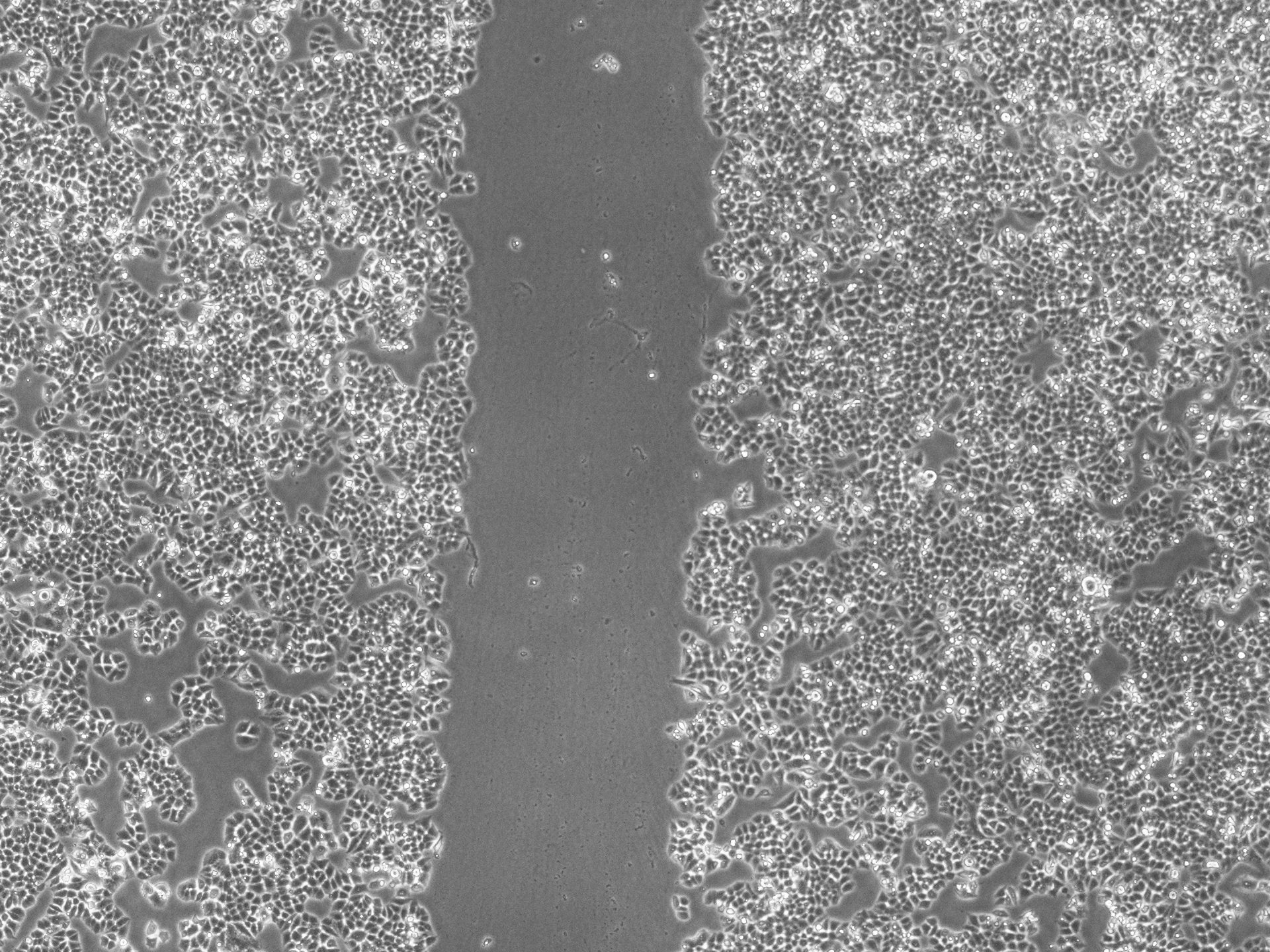

Supplement: Supplementary file 4 [file DataSheet4.zip › Huh7 wound healing assay/H7si-2 48h.tif]

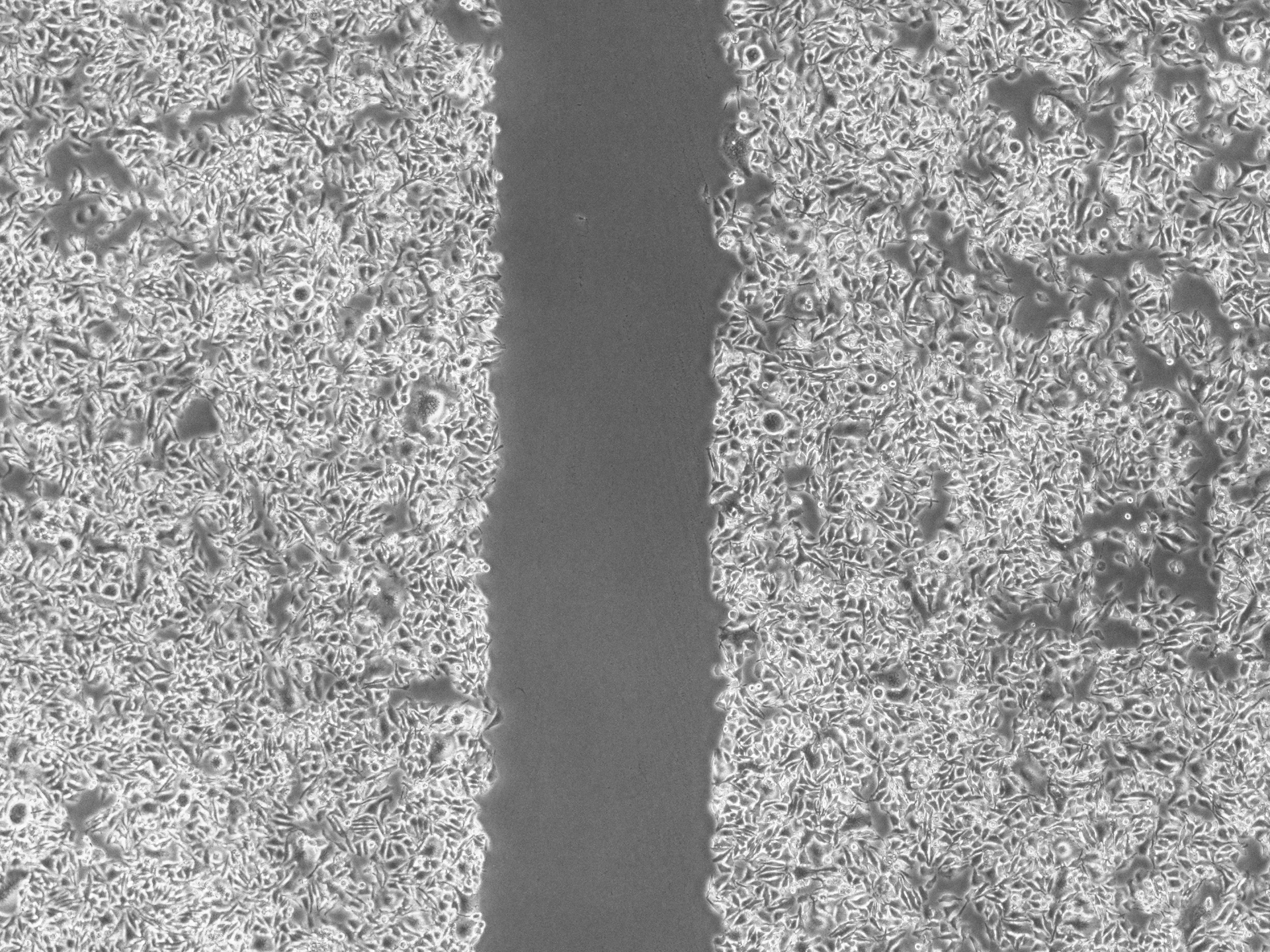

Supplement: Supplementary file 4 [file DataSheet4.zip › Huh7 wound healing assay/H7si1 0h.tif]

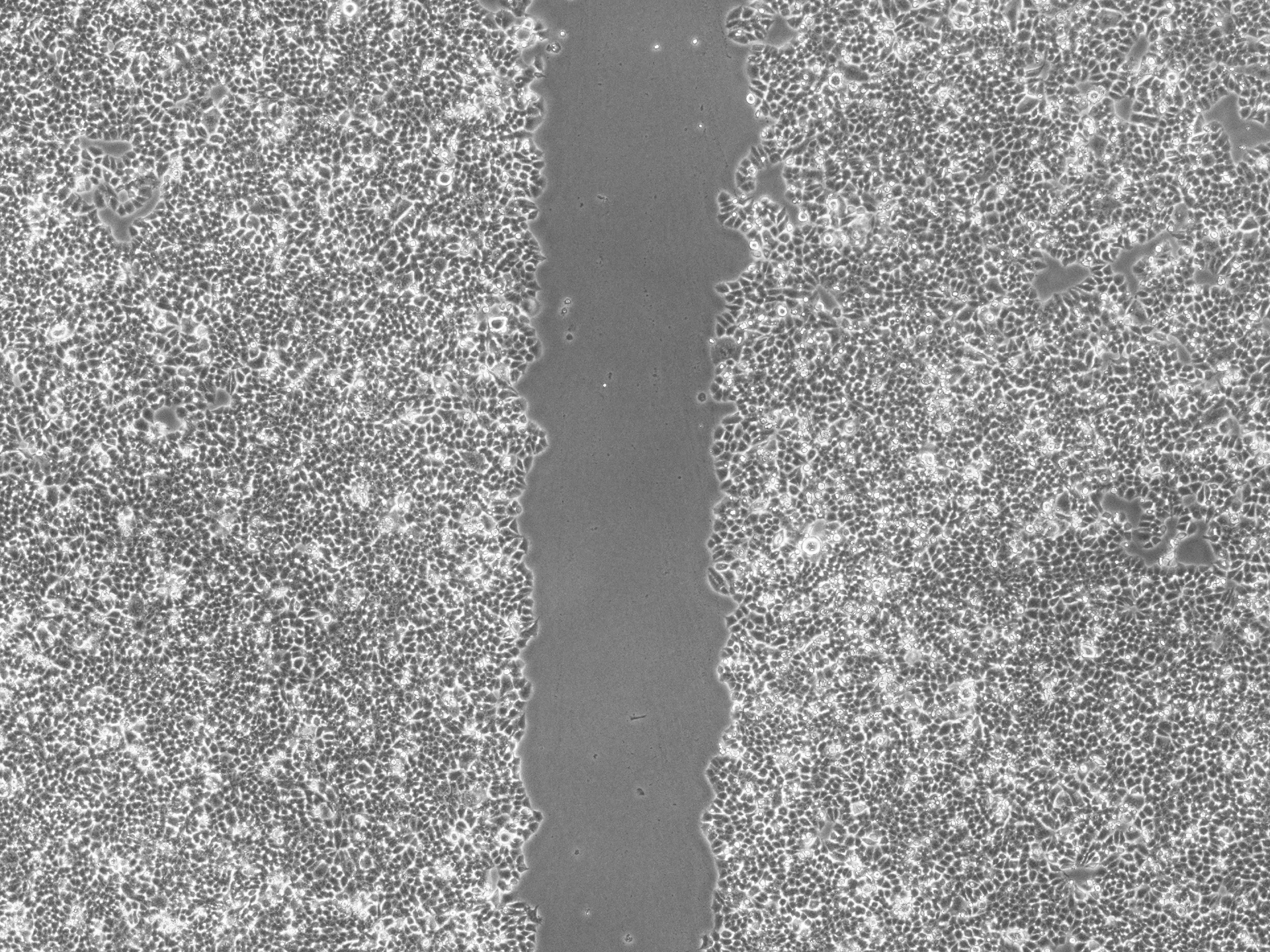

Supplement: Supplementary file 4 [file DataSheet4.zip › Huh7 wound healing assay/H7si1 48h.tif]

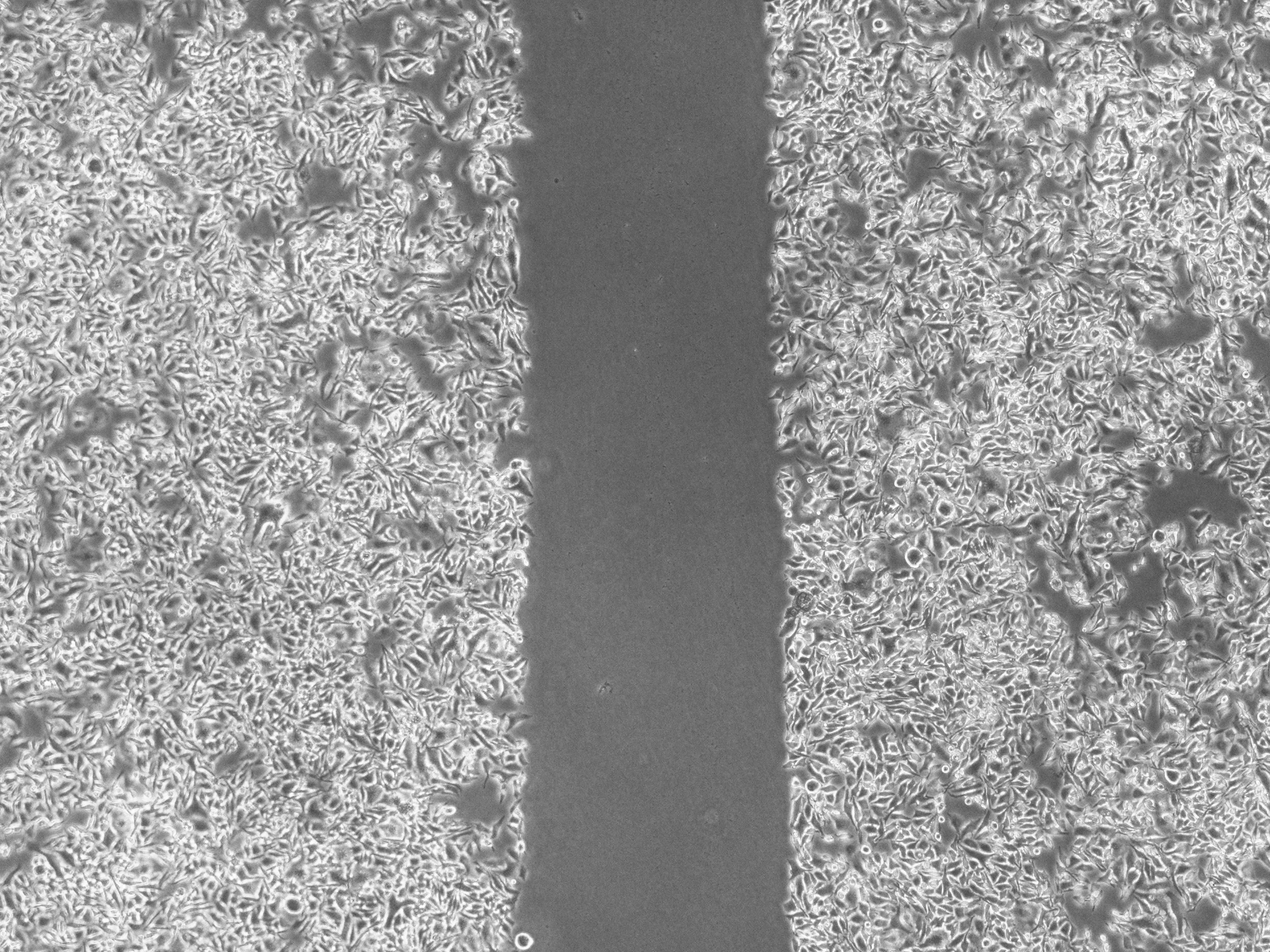

Supplement: Supplementary file 4 [file DataSheet4.zip › Huh7 wound healing assay/H7si1-1 0h.tif]

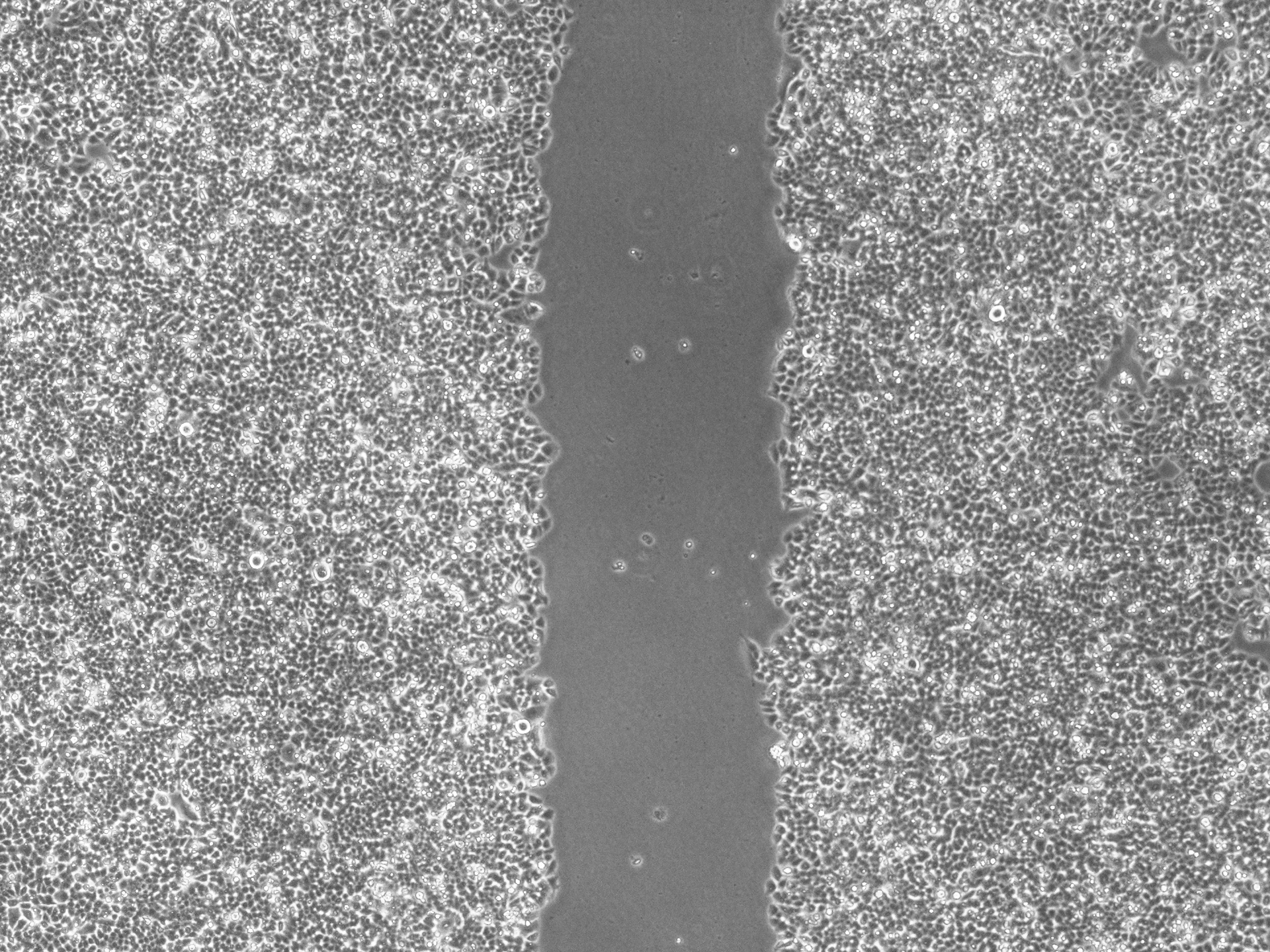

Supplement: Supplementary file 4 [file DataSheet4.zip › Huh7 wound healing assay/H7si1-1 48h.tif]

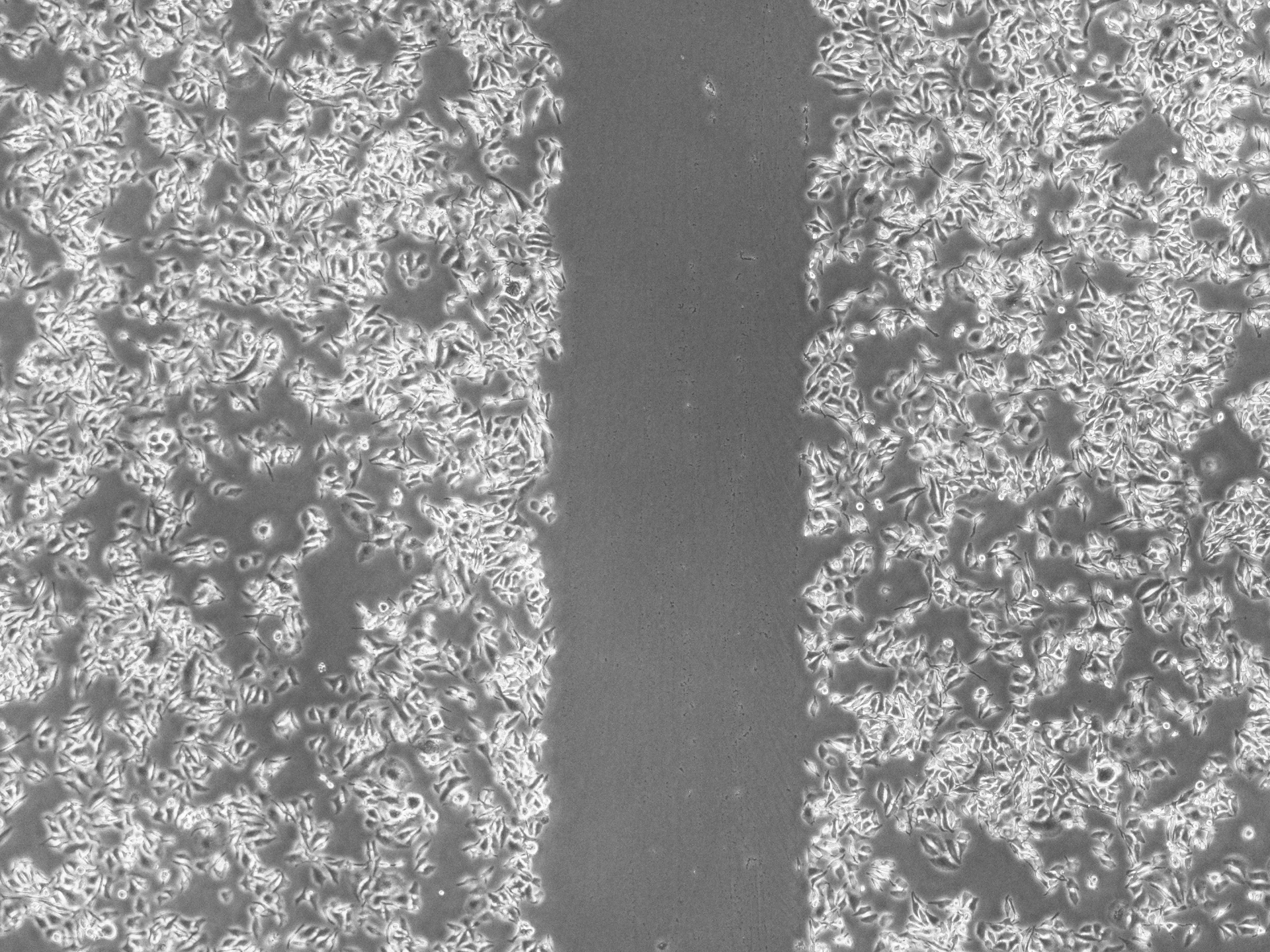

Supplement: Supplementary file 4 [file DataSheet4.zip › Huh7 wound healing assay/H7si1-2 0h.tif]
